# Synthesis of pyrrolidine-based hamamelitannin analogues as quorum sensing inhibitors in *Staphylococcus aureus*

**DOI:** 10.3762/bjoc.14.260

**Published:** 2018-11-12

**Authors:** Jakob Bouton, Kristof Van Hecke, Reuven Rasooly, Serge Van Calenbergh

**Affiliations:** 1 Laboratory for Medicinal Chemistry, Ghent University, Ottergemsesteenweg 460, 9000 Ghent, Belgium; 2 XStruct, Department of Chemistry, Ghent University, Krijgslaan 281 S3, 9000 Ghent, Belgium; 3 Western Regional Research Center, Foodborne Toxin Detection & Prevention Research Unit, Agricultural Research Service, United States Department of Agriculture, Albany, CA 94710, USA

**Keywords:** hamamelitannin, iminosugar, pyrrolidine, quorum sensing, *Staphylococcus aureus*

## Abstract

Interfering with bacterial cell-to-cell communication is a promising strategy to combat antimicrobial resistance. The natural product hamamelitannin and several of its analogues have been identified as quorum sensing inhibitors. In this paper the synthesis of pyrrolidine-based analogues of a more lead-like hamamelitannin analogue is reported. A convergent synthetic route based on a key ring-closing metathesis reaction was developed and delivered the pyrrolidine analogue in 17 steps in high yield. Chemoselective derivatization of the pyrrolidine nitrogen atom resulted in 6 more compounds. The synthesized compounds were evaluated in a biofilm model, but were all inactive.

## Introduction

Antimicrobial resistance is rapidly becoming a global threat [1–2]. It is estimated that worldwide, at least 700 000 people die every year from drug-resistant strains of common bacterial infections. Strategies to deal with the global antimicrobial resistance problem need to be multifactorial. Next to disease prevention and the development of new antibiotics, it is essential to investigate innovative strategies to combat bacterial infections [3–4]. Recently, targeting bacterial virulence has gained a lot of attention [5–7]. It has been hypothesized that by “disarming” the pathogen, rather than inhibiting its growth, selective pressure for resistance development will be much lower. Furthermore, reduction of bacterial virulence directly protects the host, and at the same time renders the bacteria more susceptible towards the host defense system and antibiotics.

**Figure 1:**
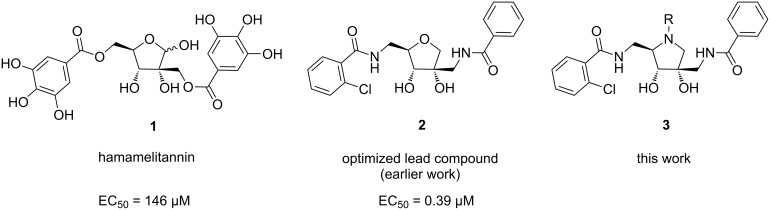
Structures of hamamelitannin (**1**), lead compound **2** and target compounds **3**.

The Centers for Disease Control and Prevention (CDC) have listed a number of bacteria that present serious, urgent and concerning threats [8]. One of these problematic bacteria is methicillin-resistant *Staphylococcus aureus* (MRSA), a human pathogen that causes a wide range of clinical infections. In *S. aureus*, virulence is mainly mediated by quorum sensing, a bacterial cell-to-cell communication system based on the secretion of signal molecules [9–11]. The natural product hamamelitannin (**1**) has been identified as a non-peptide analogue of RIP (RNAIII-inhibiting protein), an inhibitor of the RAP/TRAP (RNAIII-activating protein/target of RAP) quorum sensing system in *S. aureus* (Figure 1) [12–14]. Furthermore, hamamelitannin has been shown to inhibit biofilm formation and to potentiate the activity of antibiotics against staphylococcal biofilms in vitro and in vivo [12,15]. Structural optimization of hamamelitannin by our group resulted in several more potent and more druglike analogues of which compound **2** emerged as a promising starting point for further optimization and subsequent development (Figure 1) [16–19]. Our earlier work revealed that the optimal side chain substituents are an *o*-chlorobenzamide on the 5-position and a non-substituted benzamide on the 2’-position. In absence of any structural information of the inhibitor–target interaction, we were interested in replacing the core tetrahydrofuran scaffold by a pyrrolidine ring in order to further elucidate the structure–activity relationship. The pyrrolidine nitrogen atom provides an extra point of diversification, allowing further elaboration of the scaffold. Substituents on the ring nitrogen might lead to additional interactions with the target and therefore provide more potent analogues. Moreover, the O-to-N replacement is expected to increase solubility and possible polar interactions with the target. In this work we report the design, synthesis and biological evaluation of a number of pyrrolidine-based hamamelitannin analogues.

**Scheme 1:**
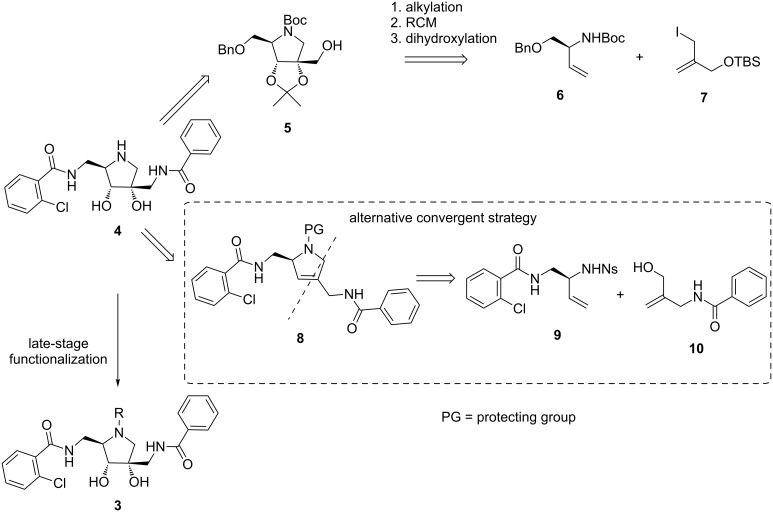
Proposed strategy for the synthesis of the target analogues.

The envisioned strategy for the synthesis of the target pyrrolidine-based hamamelitannin analogues is depicted in Scheme 1. The synthesis of **4** as a key intermediate allows to gain access to a diverse set of analogues by chemoselective late-stage derivatization of the pyrrolidine nitrogen. Previously, we used the iminosugar **5** to prepare a series of 2’-homoazanucleosides. This possible precursor was synthesized convergently in 12 steps [20]. The pyrrolidine ring was constructed via alkylation of **6** and **7**, followed by ring-closing metathesis. Stereoselective dihydroxylation of the resulting alkene then furnished the protected iminosugar **5**. However, using intermediate **5** as a starting point for the hamamelitannin analogues would render the synthetic route very linear and impractical to produce sufficient amounts required to prepare a series of analogues.

Therefore, we envisioned a modification of the previously developed synthetic route, where the two fully functionalized side chains are introduced prior to coupling and ring closure to afford **8**. This new synthetic route would be more convergent and efficient for the preparation of **3**. Retrosynthetic analysis led to **9** and **10** as two key synthons, which would be assembled via a Mitsunobu–Fukuyama reaction, and subsequently the secondary amine converted to the pyrrolidine ring via a ring-closing metathesis reaction [21].

## Results and Discussion

### Chemistry

**Scheme 2:**
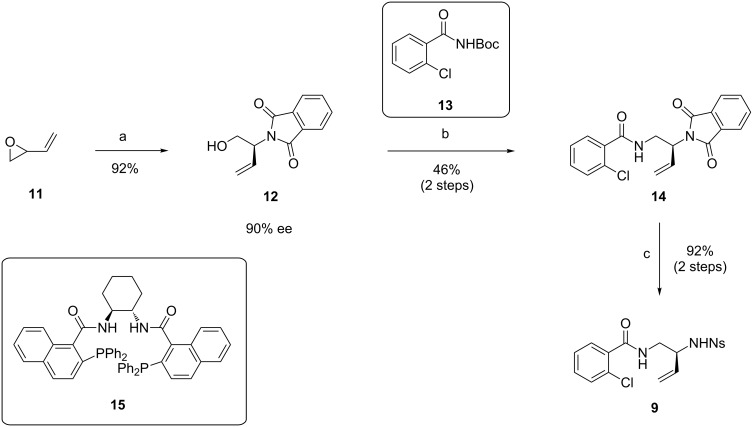
Synthesis of fragment **9**. Reagents and conditions: a) phthalimide, Pd_2_(allyl)_2_Cl_2_, ligand **15**, Na_2_CO_3_, CH_2_Cl_2_, rt; b) i) **13**, DEAD, PPh_3_, toluene, 0 °C to rt; ii) TFA, CH_2_Cl_2_, H_2_O, rt; c) i) ethylenediamine, EtOH/THF 7:3, reflux; ii) *p*-Ns-Cl, Et_3_N, THF, 0 °C.

The synthesis of fragment **9** is depicted in Scheme 2 and proved to be challenging. It was essential to introduce the nosyl group only in the last step, since several previous attempts to synthesize **9** failed due to side reactions caused by the strongly electron-withdrawing properties of the nosyl group. The successful synthesis starts with a Pd-catalyzed dynamic kinetic asymmetric transformation of racemic butadiene monoepoxide to **12**, employing phthalimide as nucleophile [22–23]. Attempts to substitute the alcohol functionality of **12** via displacement of the derived mesylate with NaN_3_ failed, similar to previously reported difficulties by Trost et al. [24]. The benzamide substituent was therefore introduced via Mitsunobu reaction with N-Boc-protected *ortho*-chlorobenzamide **13**. Removal of the Boc-group with TFA resulted in **14**. Next, the phthalimide was removed via refluxing with ethylenediamine and the resulting amine protected as *para*-nitrobenzenesulfonamide to obtain the desired fragment **9**.

**Scheme 3:**
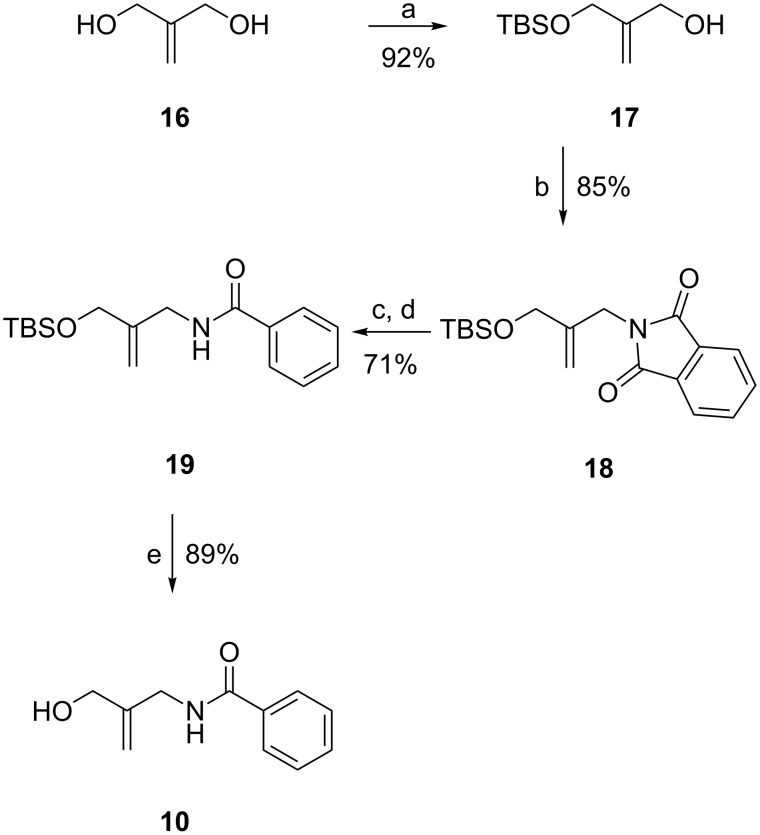
Synthesis of fragment **10**. Reagents and conditions: a) TBSCl, NaH, THF, rt; b) phthalimide, PPh_3_, DEAD, THF, 0 °C to rt; c) H_2_NNH_2_·H_2_O, MeOH, reflux; d) BzCl, Et_3_N, CH_2_Cl_2_, 0 °C; e) TBAF, THF, rt.

The synthesis of **10** starts from commercially available 2-methylene-1,3-propanediol (**16**), which was selectively monoprotected in high yield as TBS ether (Scheme 3) [25]. The remaining alcohol was then substituted for a phthalimide via Mitsunobu reaction. Phthalimide deprotection, acylation with benzoic acid, and removal of the silyl protecting group furnished **10**.

**Scheme 4:**
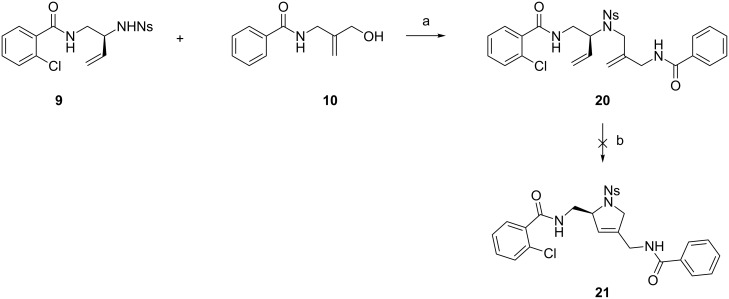
Coupling of **9** and **10** and attempted ring-closing metathesis. Reagents and conditions: a) PPh_3_, DEAD, THF/DMF 2:1, 0 °C to rt; b) 5 mol % Grubbs–Hoveyda II, 1,2-DCE, 50 °C.

Fragments **9** and **10** were coupled under Mitsunobu conditions (Scheme 4), affording **20** contaminated with Mitsunobu byproducts. Unfortunately, attempted ring-closing metathesis of **20** using the Grubbs–Hoveyda II catalyst failed to produce any product, probably due to the insolubility of **20** in solvents suitable for metathesis reactions (1,2-DCE, toluene) and/or the coordinating ability of the three (sulfon)amide functionalities [26–27].

**Scheme 5:**
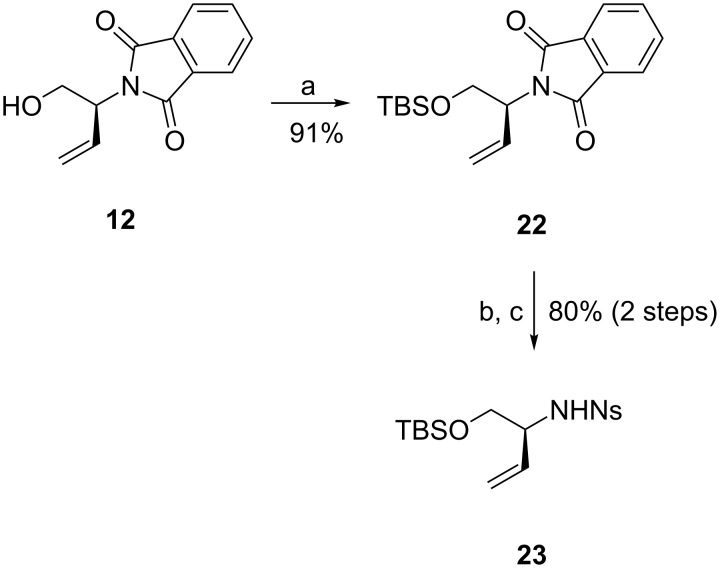
Synthesis of alternative eastern fragment **23**. Reagents and conditions: a) TBSCl, imidazole, CH_2_Cl_2_, rt; b) H_2_NNH_2_·H_2_O, MeOH, reflux; c) *p*-Ns-Cl, Et_3_N, CH_2_Cl_2_, 0 °C.

To circumvent this problem, we were forced to alter the initial synthetic strategy and used a different eastern fragment for the ring-closing metathesis reaction. The *ortho*-chlorobenzamide substituent would then be introduced in a later stage. We chose to protect alcohol **12** as TBS ether and used the derived nosyl-protected fragment **23** as the coupling partner (Scheme 5).

**Scheme 6:**
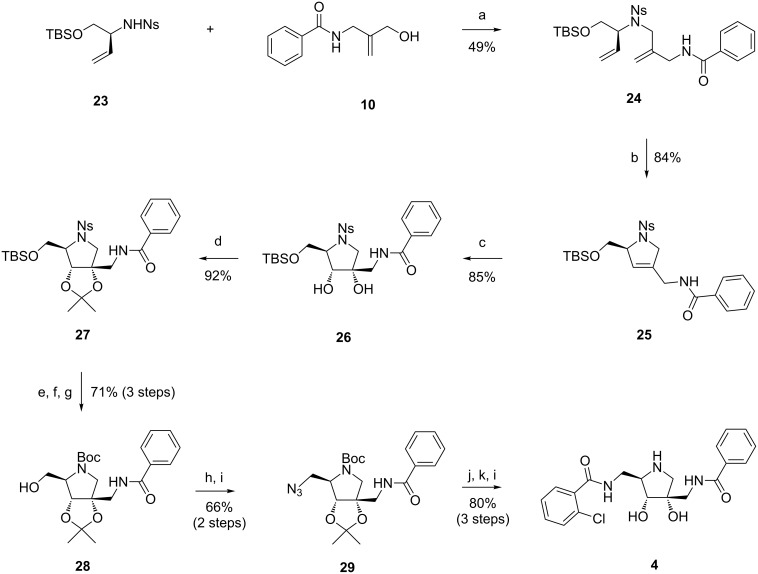
Synthesis of **4**. Reagents and conditions: a) PPh_3_, DEAD, THF, 0 °C to rt; b) Grubbs–Hoveyda II (5 mol %), 1,2-DCE, 50 °C; c) K_2_OsO_4_·2H_2_O, NMO, acetone/H_2_O 3:1, rt; d) 2-methoxypropene, CSA (cat.), THF, rt; e) PhSH, K_2_CO_3_, MeCN, 50 °C; f) Boc_2_O, Et_3_N, CH_2_Cl_2_, rt; g) TBAF, THF, rt; h) Ms-Cl, Et_3_N, CH_2_Cl_2_, 0 °C; i) NaN_3_, DMF, 60 °C; j) PMe_3_, H_2_O, THF, rt; k) 2-chlorobenzoyl chloride, Et_3_N, CH_2_Cl_2_, 0 °C; l) conc. HCl, MeOH/H_2_O 1:1, reflux.

Fragments **23** and **10** were coupled via Mitsunobu reaction, yielding **24** in 49% yield (Scheme 6). Fortunately, ring-closing metathesis of **24** now smoothly afforded **25** in high yield. Dihydroxylation selectively yielded the desired stereoisomer of diol **26**, which was subsequently protected as isopropylidene acetal. In the next step, after removal of the TBS group and mesylation, attempted substitution with NaN_3_ resulted only in an elimination product. This led us to replace the electron-withdrawing nosyl protecting group with a Boc group. After removal of the TBS ether and mesylation of the resulting alcohol, substitution with NaN_3_ now smoothly provided azide **29**. The azide was then reduced under classical Staudinger conditions, followed by amide formation with *ortho*-chlorobenzoic acid and a final acidic deprotection step to provide the desired analogue **4**. Despite the change of the initial strategy and resulting elongation of the synthetic route, the overall synthesis still proved to be very efficient, delivering **4** in 6% yield in 17 steps from **16**.

**Scheme 7:**
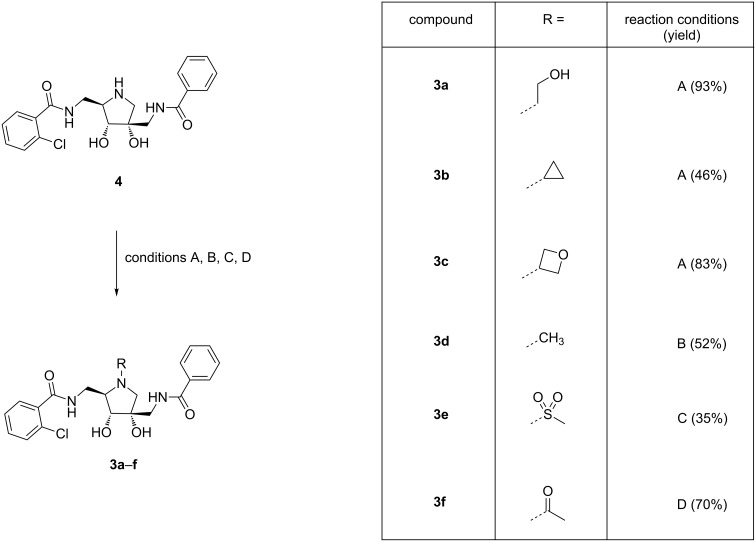
Late stage functionalization of the pyrrolidine nitrogen. Reagents and conditions: A) (masked) aldehyde, NaBH_3_CN, AcOH, MeOH, 60 °C; B) MeI, DIPEA, THF, 0 °C; C) Ms-Cl, Et_3_N, THF, 0 °C; D) AcOH, DIPEA, HATU, DMF, rt.

The pyrrolidine nitrogen was then further derivatized with several small substituents (Scheme 7). Reductive amination with several aldehydes resulted in **3a**–**c**. The N-methyl analogue **3d** was synthesized via methylation with MeI. The methanesulfonamide **3e** and acetamide **3f**, in which the cationic character of the pyrrolidine nitrogen is removed, were also synthesized.

**Figure 2:**
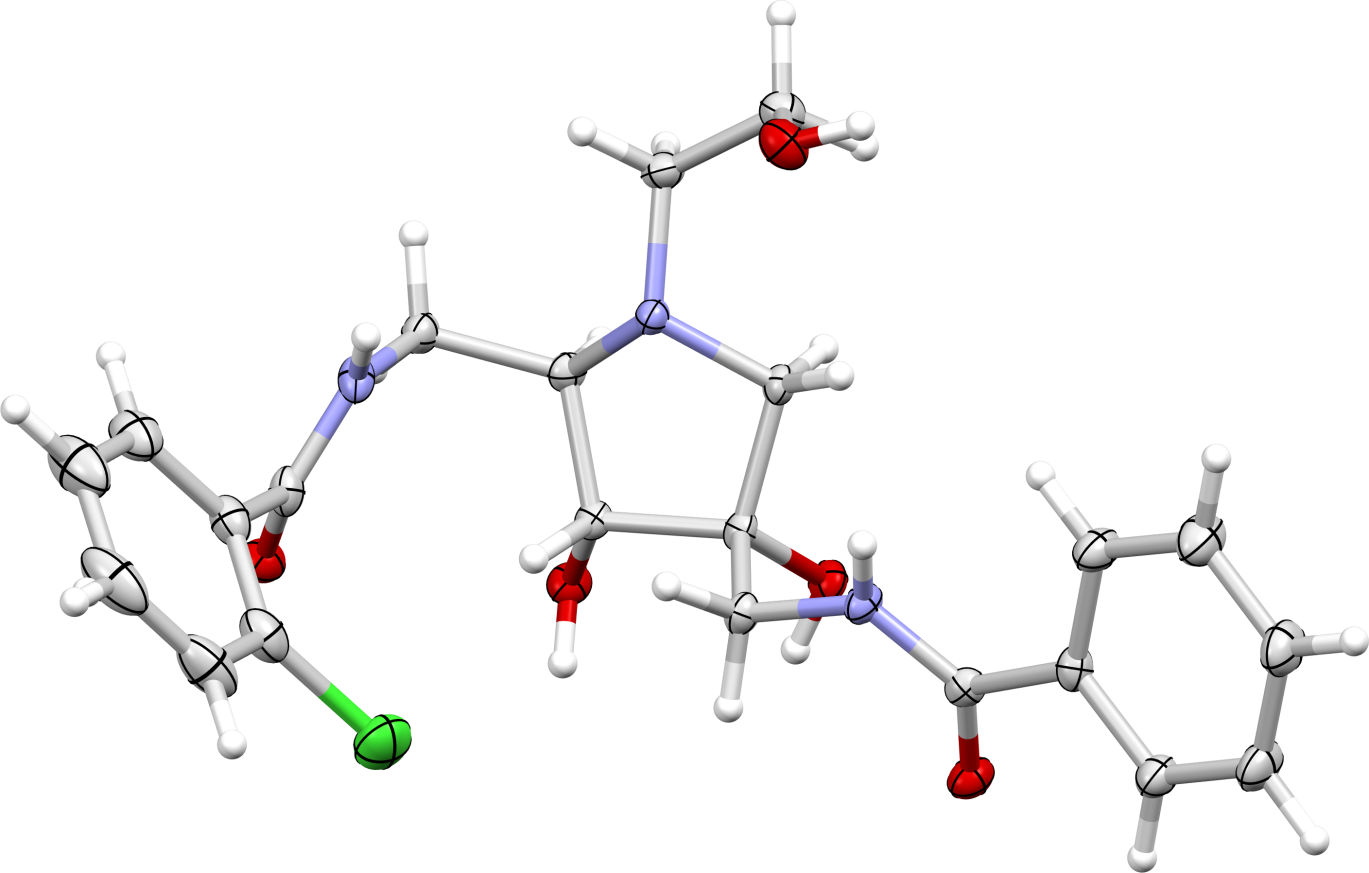
Molecular X-ray structure of **3a**, showing thermal displacement ellipsoids at the 50% probability level. Positional disorder of the chlorophenyl ring and the water solvent molecule are not shown.

The correct stereochemistry of the synthesized analogues was unequivocally proven via X-ray structural analysis of compound **3a** (Figure 2).

### Biological evaluation

The synthesized analogues were tested in a *S. aureus* biofilm model, but were all inactive (see Supporting Information File 1).

## Conclusion

A convergent synthetic route for the synthesis of pyrrolidine-based hamamelitannin analogues was developed. The originally envisioned strategy failed due to difficulties in the ring-closing metathesis reaction, but modification of one of the coupling partners solved this issue. The desired pyrrolidine-based analogue was synthesized in 17 steps and chemoselective modification of the nitrogen atom provided 6 analogues. Unfortunately, these analogues were inactive in inhibiting *S. aureus* biofilm formation.

## Supporting Information

File 1 Experimental details.
