# Supplementary material for: Synthesis of pyrrolidine-based hamamelitannin analogues as quorum sensing inhibitors in Staphylococcus aureus
Source: Beilstein J Org Chem. 2018 Nov 12;14:2822–8. doi: 10.3762/bjoc.14.260 (PMC6244240; doi:10.3762/bjoc.14.260)

Supporting Information
for
Synthesis of pyrrolidine-based hamamelitannin analogues as quorum sensing inhibitors in *Staphylococcus aureus*

Jakob Bouton[1], Kristof Van Hecke[2], Reuven Rasooly[3] and Serge Van Calenbergh*[1]

Address: [1]Laboratory for Medicinal Chemistry, Ghent University, Ottergemsesteenweg 460, 9000 Ghent, Belgium, [2]XStruct, Department of Chemistry, Ghent University, Krijgslaan 281 S3, 9000 Ghent, Belgium and [3]Western Regional Research Center, Foodborne Toxin Detection & Prevention Research Unit, Agricultural Research Service, United States Department of Agriculture, Albany, CA 94710, USA

Email: Serge Van Calenbergh - serge.vancalenbergh@ugent.be

* Corresponding author

Experimental details

Contents

1. General S3
2. Synthetic procedures and compound characterization S4
3. Single crystal X-ray diffraction data S29
4. References S32
5. Biological evaluation S33
6. NMR spectra S34

1. General

All reactions described were performed under argon atmosphere and at ambient temperature unless stated otherwise. All reagents and solvents were purchased from Sigma Aldrich (Diegem, Belgium), Acros Organics (Geel Belgium), TCI Europe (Zwijndrecht, Belgium) or Carbosynth Ltd (Compton Berkshire, United Kingdom) and used as received. NMR solvents were purchased from Eurisotop (Saint-Aubin, France). Reactions were monitored by TLC analysis using TLC aluminium sheets (Macherey-Nagel, Alugram Sil G/UV_{254}) with detection by UV or by spraying with a solution of $(NH_4)_6Mo_7O_{24}\cdot4H_2O$ (25 g/L) and $(NH_4)_4Ce(SO_4)_4.2H_2O$ (10 g/L) in H_2SO_4 (10%) followed by charring or an aqueous solution of $KMnO_7$ (20 g/L) and K_2CO_3 (10 g/L) followed by charring. Silica gel column chromatography was performed manually using Grace Davisil 60 Å silica gel (40–63 µm) or automated using a Grace Reveleris X2 system and the corresponding flash cartridges. High-resolution spectra were recorded with a Waters LCT Premier XE Mass spectrometer. ^{1}H and ^{13}C NMR spectra were recorded with a Varian Mercury-300BB (300/75 MHz) spectrometer. Chemical shifts are given in ppm (δ) relative to tetramethylsilane as an internal standard (^{1}H NMR) or the NMR solvent (^{13}C NMR). Coupling constants are given in Hz. Preparative HPLC purifications were carried out at 22 °C on a Waters AutoPurification System equipped with PDA and ESI-MS detection and using a Phenomenex Kinetex EVO C18 column (21.2 × 250 mm, 5µm) and a water/formic acid (0.2% v/v) to acetonitrile linear gradient system at a flow rate of 20 mL min^{-1}.

2. Synthetic procedures and compound characterization

Synthesis of (*S*)-2-(1-Hydroxybut-3-en-2-yl)isoindoline-1,3-dione (12) (Scheme 2)

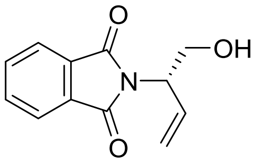

9

A 500 mL flask containing solid Na_2CO_3 (0.291 g, 2.75 mmol, 0.055 equiv) was placed under vacuum and dried with a heat gun. The flask was backfilled with N_2 gas and allylpalladium chloride dimer (0.082 g, 0.225 mmol, 0.0045 equiv), (1*R*,2*R*)-(+)-1,2-diaminocyclohexane-*N*,*N*'-bis(2-diphenylphosphino-1-naphthoyl) (CAS 174810-09-4) (0.494 g, 0.625 mmol, 0.0125 equiv) and phthalimide (7.36 g, 50.0 mmol) were added. The flask was then flushed with N_2 gas. The solids were suspended in dry degassed CH_2Cl_2 (200 mL) after which butadiene-1,3-monoepoxide (4.07 mL, 50.5 mmol, 1.01 equiv) was added. The mixture was stirred for 48 h, filtered over celite and the filtrate concentrated in vacuo. The residue was dissolved in CH_2Cl_2, absorbed onto celite and purified by flash column chromatography (10 to 40% EtOAc in hexanes).

Spectral data are in accordance with those reported in literature.[1]

Synthesis of *tert*-butyl (2-chlorobenzoyl)carbamate (13) (Scheme 2)

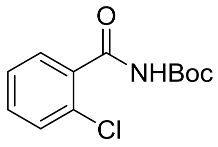

13

2-Chlorobenzamide (1.88 g, 12.0 mmol, 1.0 equiv) was dissolved in 1,2-dichloroethane (30 mL) and cooled to 0 °C. Oxalyl chloride (1.23 mL, 14.5 mmol, 1.2 equiv) was added dropwise and the temperature raised to 60°C. After 1 hour, the reaction mixture was cooled to 0 °C and *t*-BuOH (5.74 mmol mL, 60.0 mmol, 5.0 equiv) in 1,2-dichlorethane (10 mL) was added. After 3 hours, the mixture was transferred to a separation funnel. A Saturated $NaHCO_3$ solution (15 mL) was added and the mixture was extracted with 3 × 50 mL CH_2Cl_2. The combined organic layers were dried over anhydrous Na_2SO_4 and concentrated in vacuo. The residue was dissolved in CH_2Cl_2, absorbed onto celite and purified by flash column chromatography (petroleum ether/EtOAC 9:1 and 8:2). Compound **13** was obtained as a white powder (2.32 g, 9.1 mmol, 76% yield).

^{1}H NMR (300 MHz, DMSO-d_6): δ ppm 1.35 (9 H, s), 7.25 - 7.54 (5 H, m), 10.90 (1 H, s).

^{13}C NMR (75 MHz, DMSO-d_6): δ ppm 27.5, 81.1, 127.0, 128.3, 129.2, 130.9, 136.2, 150.1, 167.0. (1 quaternary carbon not found)

HRMS (ESI-TOF) *m/z*: calculated for $C_{12}H_{14}ClNNaO_3$ [M+Na] 278.0560, found 278.0558.

Synthesis of (*S*)-2-chloro-*N*-(2-(1,3-dioxoisoindolin-2-yl)but-3-en-1-yl)benzamide (14) (Scheme 2)

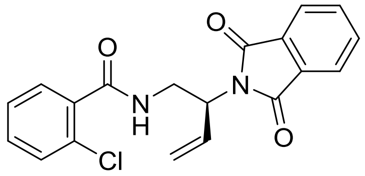

14

A solution of **12** (0.157 g, 0.72 mmol, 1.0 equiv), **13** (0.313 g, 1.22 mmol, 1.7 equiv) and PPh_3 (0.320g, 1.22 mmol, 1.7 equiv) in dry toluene (8 mL) was cooled to 0 °C. DIAD (0.240 mL, 1.22 mmol, 1.7 equiv) was added dropwise and the reaction mixture was warmed to room temperature. After 18 hours, TLC analysis (petroleum ether/EtOAc 1:1) indicated disappearance of the starting material. The mixture was concentrated in vacuo and the residue dissolved in CH_2Cl_2, absorbed onto celite and purified by flash column chromatography (0–35% EtOAc in petroleum ether) to remove the lower running impurities. The fractions containing product were isolated and concentrated in vacuo. The residue was dissolved in CH_2Cl_2 (7 mL) and TFA (3 mL) was added. After 4 hours the reaction mixture was concentrated in vacuo and co-evaporated three times with toluene to remove all TFA. The residue was redissolved in CH_2Cl_2, absorbed onto celite and purified by flash column chromatography (10–50% EtOAc in petroleum ether) to give pure **14** as a colourless oil (144 mg, 0.405 mmol, 56% yield over 2 steps).

^{1}H NMR (300 MHz, $CDCl_3$) δ ppm 3.86 (1 H, dt, *J*=14.1, 5.1 Hz), 4.26 (1 H, ddd, *J*=14.0, 9.0, 6.9 Hz), 5.07 - 5.24 (1 H, m), 5.26 - 5.45 (2 H, m), 6.22 (1 H, ddd, *J*=17.3, 10.4, 7.2 Hz), 6.62 (1 H, br. s.), 7.27 - 7.37 (3 H, m), 7.50 - 7.64 (1 H, m), 7.66 - 7.78 (2 H, m), 7.78 - 7.91 (2 H, m).

^{13}C NMR (75 MHz, $CDCl_3$) δ ppm 41.23 (1 C, s), 52.84 (1 C, s), 118.98 (1 C, s), 123.18 (1 C, s), 126.84 (1 C, s), 129.91 (1 C, s), 131.07 (1 C, s), 131.57 (1 C, s), 132.29 (1 C, s), 133.95 (1 C, s), 166.45 (1 C, s) (5 quaternary carbons missing).

HRMS (ESI-TOF) *m/z*: calculated for $C_{19}H_{16}ClN_2O_3$ [M+H] 355.0850, found 355.0855.

Synthesis of (*S*)-2-chloro-*N*-(2-((4-nitrophenyl)sulfonamido)but-3-en-1-yl)benzamide (9) (Scheme 2)

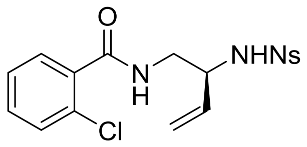

9

Compound **14** (0.256 g, 0.63 mmol) was dissolved in EtOH (7 mL) and THF (3 mL). Ethylenediamine (0.167 mL, 2.50 mmol, 4.0 equiv) was added and the reaction was heated to reflux. After 4 hours, the reaction mixture was cooled to room temperature, and filtered over celite. The filtrate was concentrated in vacuo and the residue was suspended in THF (7 mL). The suspension was cooled to 0 °C and Et_3N (0.131 mL, 0.939 mmol, 1.5 equiv) was added, followed by *p*-Ns-Cl (0.139 g, 0.626 mmol, 1.0 equiv). After 1 hour, TLC analysis (CH_2Cl_2/MeOH 9/1) indicated completion of the reaction. The reaction mixture was transferred to a separation funnel containing saturated $NaHCO_3$ (20 mL) solution and extracted with 3 × 30 mL EtOAc. The combined organic fractions were dried over anhydrous Na_2SO_4 and concentrated in vacuo. The residue was dissolved in CH_2Cl_2, absorbed onto celite and purified by flash column chromatography (10-50% EtOAc in petroleum ether) to yield **9** (115 mg, 0.280 mmol, 45% yield (2 steps)) as an orange solid.

^{1}H NMR (300 MHz, DMSO-d_6) δ ppm 3.07 - 3.37 (2 H, m), 3.91 - 4.14 (1 H, m), 4.84 - 5.10 (2 H, m), 5.54 (1 H, ddd, *J*=17.2, 10.3, 7.0 Hz), 7.25 - 7.62 (4 H, m), 7.94 - 8.13 (2 H, m), 8.30 - 8.41 (3 H, m), 8.44 (1 H, t, *J*=5.9 Hz).

^{13}C NMR (75 MHz, DMSO-d_6) δ ppm 43.1, 55.6, 117.2, 124.4, 126.9, 128.1, 128.9, 129.6, 129.9, 130.8, 135.3, 136.5, 147.3, 149.3, 166.3. (1 quaternary carbon missing)

HRMS (ESI-TOF) *m/z*: calculated for $C_{17}H_{17}ClN_3O_5S$ [M+H] 410.0577, found 410.0585.

Synthesis of 2-(((*tert*-butyldimethylsilyl)oxy)methyl)prop-2-en-1-ol (17) (Scheme 3)

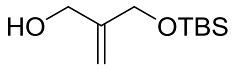

17

2-Methylene-1,3-propanediol (4.12 mL, 50.0 mmol, 1.0 equiv) in dry THF (150 mL) was added dropwise to a stirring suspension of sodium hydride (60% in mineral oil, 2.00 g, 50.0 mmol, 1.0 equiv) in dry THF (70 mL). After 45 minutes, when gas formation had ceased and a grey precipitate formed, TBSCl (3.014 g, 20.0 mmol, 1.0 equiv) was added portionwise. After 2 hours of stirring, 100 mL H_2O was added and the mixture was extracted with 3 × 100 mL EtOAc. The combined organic layers were washed with brine, dried over Na_2SO_4 and concentrated in vacuo. Purification by flash column chromatography (0 to 15% EtOAc in hexanes) yielded **17** as a colourless oil in 92% yield.

^{1}H NMR (300 MHz, $CDCl_3$) δ ppm 0.05 - 0.12 (6 H, m), 0.87 - 0.96 (9 H, m), 2.08 (1 H, br. s.), 4.17 (2 H, s), 4.21 - 4.27 (2 H, m), 5.05 - 5.15 (2 H, m).

^{13}C NMR (75 MHz, $CDCl_3$) δ ppm -5.5, 18.3, 25.6, 25.8, 64.6, 65.1, 111.1, 147.4.

HRMS (ESI-TOF) *m/z*: Calculated for $C_{11}H_{16}NO$ [M+H]: 203.1463; found 203.1462.

Synthesis of 2-(2-(((*tert*-butyldimethylsilyl)oxy)methyl)allyl)isoindoline-1,3-dione (18) (Scheme 3)

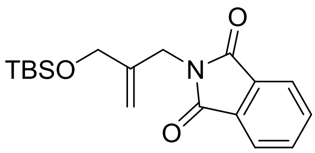

18

A solution of compound **17** (9.24 g, 45.7 mmol), PPh_3 (18.0 g, 68.5 mmol, 1.5 equiv) and phthalimide (13.4 g, 91.3 mmol, 2.0 eq) in dry THF (250 mL) was cooled to 0 °C. DEAD (40% in toluene, 25.3 mL, 68.5 mmol, 1.5 equiv) was added dropwise over 50 min. The reaction mixture was warmed to room temperature and stirred overnight. The mixture was concentrated in vacuo and the residue dissolved in CH_2Cl_2, absorbed onto celite and purified by flash column chromatography (4% and 10% Et_2O in hexanes) to yield **18** (12.9 g, 38.8 mmol, 85% yield) as a colourless oil.

1H NMR (300 MHz, $CDCl_3$) δ ppm 0.02 - 0.15 (6 H, m), 0.77 - 1.02 (9 H, m), 4.19 (2 H, s), 4.29 (2 H, s), 4.97 (1 H, d, J=1.2 Hz), 5.18 (1 H, d, J=1.2 Hz), 7.58 - 7.79 (2 H, m), 7.79 - 7.94 (2 H, m).

^{13}C NMR (75 MHz, $CDCl_3$) δ ppm 18.3, 25.8, 39.6, 64.6, 111.6, 123.3, 132.1, 133.9, 142.8, 167.9.

HRMS (ESI-TOF) *m/z*: Calculated for $C_{18}H_{26}NO_3Si$ [M+H]: 332.1682; found 332.1682.

Synthesis of 2-(((*tert*-butyldimethylsilyl)oxy)methyl)prop-2-en-1-amine (Intermediate S1) (Scheme 3)

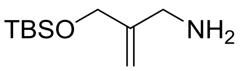

S1

Compound **18** (12.9 g, 38.8 mmol) was dissolved in MeOH (200 mL). Hydrazine hydrate (4.71 mL, 97.0 mmol, 2.5 equiv) was added and the reaction mixture was heated to reflux. When a white precipitate was formed (after ± 1 h), 50 mL of water was added and stirring was continued. After 4 hours, the mixture was concentrated in vacuo to remove most of the MeOH. The residue was dissolved in 100 mL H_2O, and transferred to a separation funnel. The water layer was extracted with 4 × 100 mL CH_2Cl_2. The combined organic phases were washed with saturated $NaHCO_3$ solution (150 mL), brine (150 mL), dried over anhydrous Na_2SO_4, and concentrated under slightly reduced pressure at 35°C, yielding pure intermediate **S1** (6.40 g, 31.8 mmol, 82% yield) as a yellow oil.

1H NMR (300 MHz, $CDCl_3$) δ ppm 0.06 (s, 6 H), 0.90 (br. s, 9 H), 1.38 (s, 2 H), 3.29 (s, 2 H), 4.16 (s, 2 H), 5.01 (d, J = 23.14 Hz, 2 H).

13C NMR (75 MHz, $CDCl_3$) δ ppm -5.4, 25.8, 44.3, 64.9, 108.3, 150.1.

HRMS (ESI-TOF) *m/z*: Calculated for $C_{10}H_{24}NOSi$ [M+H]: 202.1627; found 202.1618.

Synthesis of *N*-(2-(((*tert*-butyldimethylsilyl)oxy)methyl)allyl)benzamide (19) (Scheme 3)

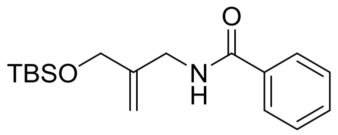

19

Intermediate **S1** (6.40 g, 31.8 mmol) was dissolved in CH_2Cl_2 (150 mL) and cooled to 0 °C. To the solution was added Et_3N (5.54 mL, 39.8 mmol, 1.25 equiv), followed by dropwise addition of Bz-Cl (3.77 mL, 32.4 mmol, 1.02 equiv). After 90 minutes the reaction was quenched by the addition of saturated $NaHCO_3$ solution (50 mL) and the biphasic mixture was transferred to a separation funnel. The phases were separated and the aequous layer was extracted once more with CH_2Cl_2 (100 mL). The combined organic phases were washed with brine, dried over anhydrous Na_2SO_4 and concentrated in vacuo. The residue was dissolved in CH_2Cl_2, absorbed onto celite and purified by flash column chromatography (petroleum ether/EtOAc 95/5 and 8/2) to give **19** (8.38g, 27.4 mmol, 86% yield) as a white solid.

^{1}H NMR (300 MHz, $CDCl_3$) δ ppm -0.01 - 0.16 (m, 6 H), 0.79 - 1.02 (m, 9 H), 4.11 (d, *J* = 5.57 Hz, 2 H), 4.22 (s, 2 H), 5.13 (d, *J* = 24.90 Hz, 1 H), 6.70 (br. s, 1 H), 7.33 - 7.56 (m, 3 H), 7.72 - 7.83 (m, 2 H).

13C NMR (75 MHz, $CDCl_3$) δ ppm -5.4, 18.3, 25.8, 42.7, 65.4, 112.1, 126.9, 128.4, 131.3, 134.5, 144.5, 167.2.

HRMS (ESI-TOF) *m/z*: Calculated for $C_{17}H_{27}NNaO_2Si$ [M+Na]: 328.1709; found 328.1714.

Synthesis of *N*-(2-(hydroxymethyl)allyl)benzamide (10) (Scheme 3)

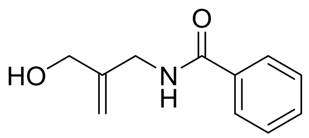

10

TBAF (1.0 M in THF, 30.0 ml, 30.0 mmol, 1.1 equiv) was added to a solution of **19** (8.32 g, 27.2 mmol) in THF (200 mL). After 2 hours, most of the solvent was removed by concentration under reduced pressure. The residue was dissolved in EtOAC (200 mL), transferred to a separation funnel and washed with saturated NH_4Cl solution (60 mL), H_2O (60 mL), and brine (60 mL). The organic layer was dried over Na_2SO_4, and concentrated in vacuo. The residue was dissolved in CH_2Cl_2, absorbed onto celite and purified by flash column chromatography (petroleum ether/EtOAc 9:1 and 8:2). Compound **10** (4.66 g, 24.4 mmol, 89% yield) was obtained as a white solid.

^{1}H NMR (300 MHz, $CDCl_3$) δ ppm 3.65 (br. s., 1 H), 3.99 - 4.22 (m, 4 H), 5.06 (d, *J* = 13.20 Hz, 2 H), 6.94 (br. s., 1 H), 7.32 - 7.63 (m, 3 H), 7.70 - 7.92 (m, 2 H).

^{13}C NMR (75 MHz, $CDCl_3$) δ ppm 42.5, 64.2, 113.3, 127.0, 128.6, 131.7, 133.9, 145.3, 168.3.

HRMS (ESI-TOF) *m/z*: Calculated for $C_{11}H_{14}NO_2$ [M+H]: 192.1025; found 192.1015.

(*S*)-*N*-(2-((*N*-(2-(Benzamidomethyl)allyl)-4-nitrophenyl)sulfonamido)but-3-en-1-yl)-2-chlorobenzamide (20) (Scheme 4)

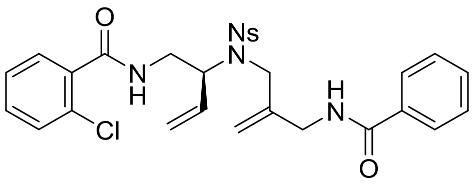

20

Compound **9** (0.093 mg, 0.227 mmol, 1.2 equiv) ,**10** (0.036 mg, 0.189 mmol) and PPh_3 (0.069 mg, 0.265 mmol, 1.4 equiv) were dissolved in a mixture of THF (4 mL) and DMF (2 mL). The solution was cooled to 0 °C and DEAD (40% wt in toluene, 0.097 mL, 0.256 mmol, 1.4 equiv) was added dropwise. The reaction was warmed to room temperature and stirred for 4 hours after which it was concentrated in vacuo. The residue was suspended in CH_2Cl_2, absorbed onto celite and purified by flash column chromatography (10–45% ethyl acetate in toluene). After chromatography, the product was still slightly contaminated with Mitsunobu sideproducts and was therefore used without further characterization in the next reaction.

HRMS (ESI-TOF) *m/z*: Calculated for $C_{28}H_{28}ClN_4O_6S$ [M+H]: 583.1418; found 583.1389.

Synthesis of (*S*)-2-(1-((*tert*-butyldimethylsilyl)oxy)but-3-en-2-yl)isoindoline-1,3-dione (22) (Scheme 5)

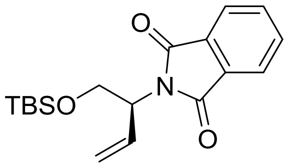

22

Compound **12** (6.67 g, 30.7 mmol) was dissolved in CH_2Cl_2 (100 mL). Imidazole (2.51 g, 36.85 mmol, 1.2 equiv) was added, followed by TBS-Cl (5.09 g, 33.8 mmol, 1.1 equiv). The solution was stirred at room temperature for 90 minutes after which it was diluted with saturated $NaHCO_3$ solution (60 mL). The biphasic mixture was transferred to a separation funnel, the phases were separated and the aequous layer was extracted two more times with CH_2Cl_2 (100 mL). The combined organic phases were dried over anhydrous $MgSO_4$ and concentrated in vacuo. The residue was dissolved in CH_2Cl_2, absorbed onto celite and purified by flash column chromatography (petroleum ether/EtOAc 95/5) to obtain **22** (9.25 g, 27.89 mmol, 91% yield) as a white solid.

^{1}H NMR (300 MHz, $CDCl_3$) δ ppm -0.07 (3 H, s), 0.00 (3 H, s), 0.72 - 0.79 (9 H, m), 3.87 (1 H, dd, *J*=10.0, 6.2 Hz), 4.16 (1 H, t, *J*=9.7 Hz), 4.79 - 4.99 (1 H, m), 5.18 - 5.41 (2 H, m), 6.18 (1 H, ddd, *J*=17.4, 10.3, 7.5 Hz), 7.65 - 7.77 (2 H, m), 7.77 - 7.90 (2 H, m).

^{13}C NMR (75 MHz, $CDCl_3$) δ ppm -5.6, -5.5, 17.9, 25.6, 55.8, 62.2, 118.9, 123.1, 132.0, 132.3, 133.8, 168.2.

HRMS (ESI-TOF) *m/z*: Calculated for $C_{18}H_{26}NO_3Si$ [M+H]: 332.1682; found 332.1674.

Synthesis of (*S*)-*N*-(1-((*tert*-butyldimethylsilyl)oxy)but-3-en-2-yl)-4-nitrobenzenesulfonamide (23) (Scheme 5)

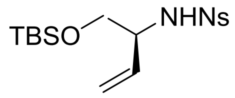

23

To a solution of compound **22** (9.25 g, 27.9 mmol) in MeOH was added hydrazine hydrate (4.75 mL, 97.7 mmol, 3.5 equiv). The solution was stirred for 3 hours at 65°C and concentrated in vacuo. The residue was dissolved in H_2O (70 mL), transferred to a separation funnel and extracted with CH_2Cl_2 (4 × 60 mL). The combined organic phases were washed with saturated $NaHCO_3$ solution (100 mL) and brine (100 mL), dried over anhydrous Na_2SO_4 and concentrated in vacuo. The residue was then redissolved in CH_2Cl_2 (100 mL) and cooled to 0 °C. Et_3N (4.73 mL, 30.7 mmol, 1.1 equiv) and *p*-Ns-Cl (6.18 g, 27.9 mmol, 1.0 equiv) were added, and after 90 minutes TLC analysis (petroleum ether/EtOAc 7:3) indicated completion of the reaction. The mixture was transferred to a separation funnel containing saturated $NaHCO_3$ solution (80 mL). The phases were separated and the aqueous layer was extracted two more times with CH_2Cl_2 (100 mL). The combined organic layers were dried over Na_2SO_4 and concentrated in vacuo. The residue was dissolved in CH_2Cl_2, absorbed onto celite and purified by flash column chromatography (petroleum ether/EtOAc 95:5) to yield **23** (8.23 g, 22.3 mmol, 80% over 2 steps) as a white powder.

^{1}H NMR (300 MHz, $CDCl_3$) δ ppm -0.08 - 0.06 (6 H, m), 0.79 - 0.88 (9 H, m), 3.56 (1 H, dd, *J*=10.3, 5.3 Hz), 3.60 (1 H, dd, *J*=10.3, 4.1 Hz), 3.80 - 3.95 (1 H, m), 5.06 - 5.18 (2 H, m), 5.21 (1 H, d, *J*=6.7 Hz), 5.62 (1 H, ddd, *J*=17.2, 10.3, 6.7 Hz), 7.97 - 8.17 (2 H, m), 8.27 - 8.45 (2 H, m).

^{13}C NMR (75 MHz, $CDCl_3$) δ ppm -5.6, -5.5, 18.2, 25.7, 58.0, 65.2, 118.0, 124.1, 128.4, 134.6, 146.7, 149.9.

HRMS (ESI-TOF) *m/z*: Calculated for $C_{16}H_{27}N_2O_5SSi$ [M+H]: 387.1410; found 387.1403.

Synthesis of (*S*)-*N*-(2-(((*N*-(1-((*tert*-butyldimethylsilyl)oxy)but-3-en-2-yl)-4-nitrophenyl)sulfonamido)methyl)allyl)benzamide (24) (Scheme 6)

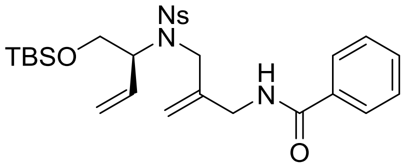

24

Compound **23** (4.06 g, 10.49 mmol), **10** (2.609 g, 13.6 mmol, 1.3 equiv) and PPh_3 (4.13 g, 15.8 mmol, 1.5 equiv) were dissolved in anhydrous THF (100 mL). The solution was cooled to 0°C and DEAD (40% wt. in toluene, 5.79 mL, 15.7 mmol, 1.5 equiv) was added dropwise. After 8 hours stirring at room temperature the reaction mixture was concentrated in vacuo, redissolved in CH_2Cl_2, absorbed onto celite and purified by flash column chromatography (petroleum ether/ethyl acetate 85:15 and 8:2). Compound **23** (2.90 g, 5.18 mmol, 49% yield) was obtained as a yellow oil.

^{1}H NMR (300 MHz, $CDCl_3$) δ ppm -0.01 (3 H, s), 0.01 (3 H, s), 0.81 (9 H, s), 3.71 (1 H, dd, *J*=10.8, 5.9 Hz), 3.85 (1 H, dd, *J*=10.8, 8.2 Hz), 3.94 (2 H, s), 4.19 (2 H, d, *J*=6.2 Hz), 4.32 - 4.47 (1 H, m), 5.10 - 5.26 (3 H, m), 5.33 (1 H, s), 5.55 - 5.84 (1 H, m), 7.15 (1 H, t, *J*=6.2 Hz), 7.39 - 7.57 (3 H, m), 7.82 - 7.91 (2 H, m), 7.97 - 8.07 (2 H, m), 8.26 - 8.39 (2 H, m).

^{13}C NMR (75 MHz, $CDCl_3$) δ ppm -5.5, -5.5, 0.0, 18.2, 25.7, 42.0, 48.5, 63.1, 117.4, 120.4, 124.2, 126.9, 127.0, 128.5, 128.6, 131.5, 132.7, 134.1, 141.8, 146.0, 149.9, 166.9.

HRMS (ESI-TOF) *m/z*: Calculated for C27H38N3O6SSi [M+H]: 560.2251; found 560.2246.

Synthesis of (*S*)-*N*-((5-(((*tert*-butyldimethylsilyl)oxy)methyl)-1-((4-nitrophenyl)sulfonyl)-2,5-dihydro-1*H*-pyrrol-3-yl)methyl)benzamide (25) (Scheme 6)

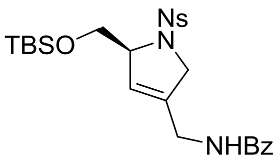

25

Compound **24** (2.90 g, 5.18 mmol, 1.0 equiv) was dissolved in dry degassed 1,2-dichloroethane (25 mL). The reaction was warmed to 50 °C and a solution of Grubbs–Hoveyda II catalyst (0.097 g, 0.155 mmol, 3 mol %) in 1,2-dichloroethane (1 mL) was added. After 4 hours a second portion of catalyst (0.065 g, 0.103 mmol, 2 mol %) in 1,2-dichloroethane (1 mL) was added and stirring was continued overnight. The reaction mixture was concentrated in vacuo, the residue redissolved in CH_2Cl_2, absorbed onto celite and purified by flash column chromatography (22% EtOAc in petroleum ether) to yield **25** (2.30 g, 4.33 mmol, 84% yield) as a brown foam.

^{1}H NMR (300 MHz, $CDCl_3$) δ ppm 0.05 (3 H, s), 0.06 (3 H, s), 0.85 (9 H, s), 3.72 (1 H, dd, *J*=10.0, 6.7 Hz), 3.91 (1 H, dd, *J*=10.0, 3.5 Hz), 4.04 - 4.24 (4 H, m), 4.35 - 4.59 (1 H, m), 5.60 (1 H, m, *J*=1.8 Hz), 6.24 (1 H, br. t, *J*=6.2, 6.2 Hz), 7.38 - 7.50 (2 H, m), 7.53 (1 H, d, *J*=7.3 Hz), 7.64 - 7.78 (2 H, m), 7.90 - 8.08 (2 H, m), 8.17 - 8.36 (2 H, m).

^{13}C NMR (75 MHz, $CDCl_3$) δ ppm -5.4, -5.4, 18.1, 25.7, 37.7, 56.2, 65.8, 68.7, 124.0, 124.4, 126.8, 128.4, 128.7, 131.9, 133.6, 137.1, 143.5, 150.1, 167.3.

HRMS (ESI-TOF) *m/z*: Calculated for $C_{25}H_{34}N_3O_6SSi$ [M+H]: 532.1938; found 532.1925.

Synthesis of *N*-(((3*S*,4*R*,5*R*)-5-(((*tert*-butyldimethylsilyl)oxy)methyl)-3,4-dihydroxy-1-((4-nitrophenyl)sulfonyl)pyrrolidin-3-yl)methyl)benzamide (26) (Scheme 6)

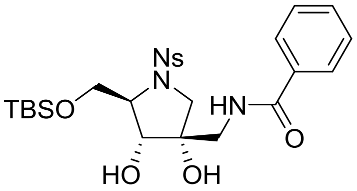

26

Compound **25** (2.51 g, 4.73 mmol, 1.0 equiv) was dissolved in a mixture of acetone (30 mL) and H_2O (10 mL). $K_2OsO_4 \cdot 2H_2O$ (0.032 g, 0.087 mmol, 2 mol %) was added, followed by NMO (0.761 g, 6.50 mmol, 1.5 equiv). The reaction was stirred overnight, and quenched by the addition of solid Na_2SO_3 (3.2 g). Stirring was continued for 1 hour, and the solvents removed by concentration under reduced pressure. The residue was dissolved in H_2O (25 mL), transferred to a separation funnel, and exracted with EtOAc (3 × 50 mL). The combined organic layers were washed with brine, dried over anhydrous Na_2SO_4 and concentrated in vacuo. The residue was dissolved in CH_2Cl_2, absorbed onto celite and purified by flash column chromatography (5–50% EtOAc in petroleum ether) to yield single diastereoisomer **26** (2.28 g, 4.03 mmol, 85% yield) as a white foam.

^{1}H NMR (300 MHz, $CDCl_3$) δ ppm 0.07 (3 H, s), 0.08 - 0.11 (3 H, m), 0.82 - 0.84 (9 H, m), 3.30 - 3.37 (1 H, m), 3.37 - 3.48 (5 H, m), 3.55 (1 H, d, *J*=12.0 Hz), 3.60 (1 H, dd, *J*=14.6, 7.0 Hz), 3.97 (1 H, dd, *J*=10.5, 2.6 Hz), 4.07 (1 H, m, *J*=4.1 Hz), 4.22 (1 H, dd, *J*=7.0, 5.3 Hz), 6.74 (1 H, t, *J*=5.9 Hz), 7.35 - 7.49 (2 H, m), 7.53 (1 H, d, *J*=7.3 Hz), 7.65 - 7.81 (2 H, m), 7.91 - 8.08 (2 H, m), 8.22 - 8.44 (2 H, m).

^{13}C NMR (75 MHz, $CDCl_3$) δ ppm -5.5, -5.4, 18.1, 25.8, 44.4, 56.2, 63.0, 65.1, 74.6, 76.9, 123.9, 127.0, 128.7, 129.0, 132.3, 132.8, 143.0, 150.1, 169.7.

HRMS (ESI-TOF) *m/z*: Calculated for $C_{25}H_{36}N_3O_8SSi$ [M+H]: 566.1992; found 566.2020.

Synthesis of *N*-(((3a*S*,6*R*,6a*R*)-6-(((*tert*-butyldimethylsilyl)oxy)methyl)-2,2-dimethyl-5-((4-nitrophenyl)sulfonyl)tetrahydro-3a*H*-[1,3]dioxolo[4,5-*c*]pyrrol-3a-yl)methyl)benzamide (27) (Scheme 6)

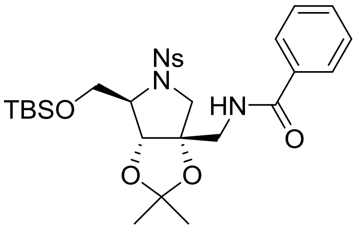

27

Compound 26 (2.201 g, 3.89 mmol, 1.0 eq) was dissolved in dry THF (120 mL). 2-Methoxypropene (3.60 mL, 38.9 mmol, 10.0 equiv) was added, followed by camphorsulphonic acid (0.018 g, 0.078 mmol, 2 mol %) The reaction was stirred overnight and neutralized by the addition of Et3N (100 µL). The mixture was concentrated in vacuo, the residue dissolved in CH_2Cl_2, absorbed onto celite and purified by flash column chromatography (0–35% EtOAc in petroleum ether). **27** was obtained as a white foam (2.17 g, 3.58 mmol, 92% yield).

^{1}H NMR (300 MHz, $CDCl_3$) δ ppm 0.11 (3 H, s), 0.12 - 0.15 (3 H, m), 0.86 - 0.93 (9 H, m), 1.03 (3 H, s), 1.32 (3 H, s), 3.54 - 3.71 (3 H, m), 3.89 (2 H, d, *J*=4.4 Hz), 4.00 - 4.13 (2 H, m), 4.52 (1 H, s), 6.24 - 6.50 (1 H, m), 7.34 - 7.63 (3 H, m), 7.71 - 7.86 (2 H, m), 7.96 - 8.14 (2 H, m), 8.25 - 8.45 (2 H, m).

^{13}C NMR (75 MHz, $CDCl_3$) δ ppm -5.4, 18.5, 26.0, 26.9, 27.3, 44.7, 58.4, 64.5, 68.1, 76.6, 77.4, 85.2, 90.6, 112.3, 124.3, 126.9, 128.5, 128.7, 131.8, 133.9, 145.1, 150.0, 167.4.

HRMS (ESI-TOF) *m/z*: Calculated for $C_{28}H_{40}N_3O_8SSi$ [M+H]: 606.2305; found 606.2301.

Synthesis of *tert*-butyl-(3a*S*,6*R*,6a*R*)-3a-(benzamidomethyl)-6-(hydroxymethyl)-2,2-dimethyltetrahydro-5*H*-[1,3]dioxolo[4,5-*c*]pyrrole-5-carboxylate (28) (Scheme 6)

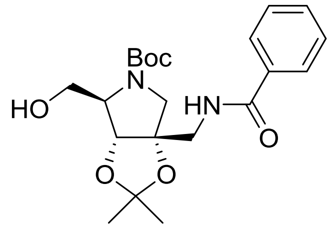

28

To a solution of **27** (2.17 g, 3.58 mmol) in MeCN (30 mL) were added K_2CO_3 (1.48 g, 10.74 mmol, 3.0 equiv) and thiophenol (0.551 mL, 5.37 mmol, 1.5 equiv). The mixture was warmed to 50 °C and stirred for 36 hours after which it was filtered over celite and conentrated in vacuo. The residue was dissolved in CH_2Cl_2, absorbed onto celite and purified by quick flash column chromatography (0–5% MeOH in CH_2Cl_2), to give a mixture of product and close-running impurities. This residue was dissolved in CH_2Cl_2 (30 mL). Et_3N (0.600 mL, 4.30 mmol, 1.2 equiv) and Boc_2O (0.987 mL, 4.30 mmol, 1.2 equiv) were added and the reaction was stirred overnight. A saturated $NaHCO_3$ solution (30 mL) was added and the biphasic mixture was transferred to a separation funnel. The aequous layer was extracted with CH_2Cl_2 (3 × 50 mL). The combined organic fractions were dried over Na_2SO_4 and concentrated in vacuo. The residue was dissolved in THF (30 mL) and TBAF (1.0 M in THF) was added. After 3 hours, the reaction mixture was concentrated in vacuo, the residue dissolved in CH_2Cl_2, absorbed onto celite and purified by flash column chromatography (petroleum ether/EtOAc 65:35 and 1:1) to give **28** (1.040 g, 2.56 mmol, 71% yield over 3 steps) as a colourless oil.

^{1}H NMR (300 MHz, DMSO-d_6, 80°C) δ ppm 1.34 (3 H, s), 1.35 (3 H, s), 1.40 - 1.43 (9 H, m), 3.38 - 3.47 (2 H, m), 3.51 (1 H, dd, *J*=10.5, 4.1 Hz), 3.61 - 3.73 (3 H, m), 3.76 - 3.99 (1 H, m), 4.65 (1 H, s), 7.40 - 7.58 (3 H, m), 7.82 - 7.91 (2 H, m), 8.27 - 8.43 (1 H, m).

^{13}C NMR (75 MHz, DMSO-d_6, 80°C) δ ppm 27.21 (1 C, s), 27.53 (1 C, s), 27.74 (1 C, s), 43.01 (1 C, s), 54.85 (1 C, s), 59.96 (1 C, s), 65.37 (1 C, s), 78.30 (1 C, s), 78.71 (1 C, s), 84.31 (1 C, s), 110.83 (1 C, s), 126.83 (1 C, s), 127.74 (1 C, s), 130.66 (1 C, s), 134.14 (1 C, s), 153.02 (1 C, s), 166.59 (1 C, s) .

HRMS (ESI-TOF) *m/z*: Calculated for $C_{21}H_{31}N_2O_6$ [M+H]: 407.2182; found 407.2200.

Synthesis of *tert*-butyl-(3a*S*,6*R*,6a*R*)-6-(azidomethyl)-3a-(benzamidomethyl)-2,2-dimethyltetrahydro-5*H*-[1,3]dioxolo[4,5-*c*]pyrrole-5-carboxylate (29) (Scheme 6)

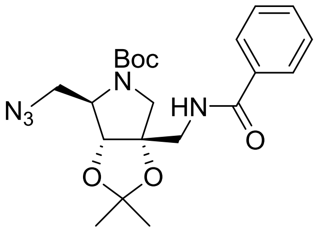

29

Compound **28** (0.950 g, 2.34 mmol) was dissolved in CH_2Cl_2 (15 mL). The reaction was cooled to 0 °C and Et_3N (0.489 mL, 3.51 mmol, 1.5 equiv) was added, followed by dropwise addition of Ms-Cl (0.226 mL, 2.92 mmol, 1.25 equiv). After 1 hour of stirring, the reaction mixture was diluted with 50 mL CH_2Cl_2 and transferred to a separation funnel. A saturated $NaHCO_3$ solution (30 mL) was added and the phases were separated. The organic layer was dried over anhydrous sodium sulfate and concentrated in vacuo. The residue was dissolved in DMF (20 mL), NaN_3 (0.770 g, 11.7 mmol, 5.0 equiv) was added and the reaction was stirred overnight at 60 °C. The reaction mixture was diluted with 30 mL H_2O, transferred to a separation funnel and extracted with EtOAc (3 × 50 mL). The combined organic fractions were dried over anhydrous Na_2SO_4 and concentrated in vacuo. The residue was dissolved in CH_2Cl_2, absorbed onto celite and purified by flash column chromatography (10–40% EtOAc in petroleum ether) to yield **29** (0.671 g, 1.56 mmol, 66% yield) as a white foam.

^{1}H NMR (300 MHz, DMSO-*d_6*, 80°C) δ ppm 1.35 (3 H, s), 1.37 (3 H, s), 1.43 (9 H, s), 3.41 - 3.51 (3 H, m), 3.59 - 3.77 (3 H, m), 4.04 (1 H, t, *J*=6.2 Hz), 4.57 (1 H, s), 7.40 - 7.58 (3 H, m), 7.83 - 7.94 (2 H, m), 8.42 (1 H, t, *J*=6.4 Hz).

^{13}C NMR (75 MHz, DMSO-d_6, 80°C) δ ppm 27.1, 27.5, 27.6, 42.7, 50.1, 54.2, 63.1, 79.0, 84.3, 90.4, 111.2, 126.8, 127.8, 130.8, 133.9, 152.9, 166.7.

HRMS (ESI-TOF) *m/z*: Calculated for $C_{21}H_{30}N_5O_5$ [M+H]: 432.2247; found 432.2250.

Synthesis of *N*-(((2*R*,3*R*,4*R*)-4-(benzamidomethyl)-3,4-dihydroxypyrrolidin-2-yl)methyl)-2-chlorobenzamide (4) (Scheme 6)

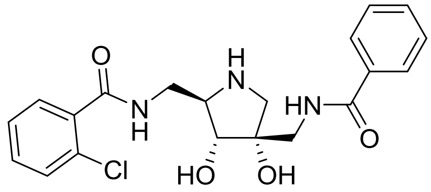

4

PMe_3 (1.0 M in THF, 3.06 mL, 3.06 mmol, 2.0 equiv) was added to a solution of **29** (0.671 g, 1.53 mmol) in THF (15 mL), and the reaction was stirred for 1 hour when H_2O (100 µL) was added. After 1 more hour, the reaction mixture was concentrated in vacuo and coevaporated three times with toluene. The residue was dissolved in in CH_2Cl_2 (30 mL) and the solution cooled to 0 °C. Et_3N (0.235 mL, 1.68 mmol, 1.1 equiv) was added, followed by dropwise addition of 2-chloro-benzoyl chloride (0.204 mL, 1.61 mmol, 1.05 equiv) over 30 minutes. After 1 more hour, TLC analysis (CH_2Cl_2/MeOH 9:1) indicated completion of the reaction and the presence of a single higher-running spot. A saturated $NaHCO_3$ solution (30 mL) was added and the biphasic mixture was transferred to a separation funnel and extracted with 3 × 50 mL CH_2Cl_2. The combined organic phases were dried over anhydrous Na_2SO_4 and concentrated in vacuo. The residue was dissolved in a mixture of MeOH (4 mL) and H_2O (4 mL), conc. HCl (2 mL) was added and the solution was heated to reflux. After 4 hours, TLC analysis (CH_2Cl_2/MeOH/NH_4OH 84:15:1) indicated completion of the reaction. The mixture was concentrated in vacuo, the residue dissolved in MeOH, absorbed onto celite and purified by flash column chromatography (CH_2Cl_2/MeOH/NH_4OH 99:0:1 to 79:20:1). **4** was obtained as a white solid (491 mg, 1.22 mmol, 80% yield over 3 steps).

^{1}H NMR (300 MHz, CD_3OD) δ ppm 2.91 (1 H, d, *J*=12.3 Hz), 3.15 (1 H, d, *J*=12.3 Hz), 3.23 - 3.30 (1 H, m), 3.48 - 3.71 (5 H, m), 7.29 - 7.57 (7 H, m), 7.81 - 7.87 (2 H, m).

^{13}C NMR (75 MHz, CD_3OD) δ ppm 42.8, 46.4, 54.7, 63.3, 77.4, 79.7, 128.0, 128.3, 129.4, 129.9, 130.8, 131.7, 132.1, 132.6, 135.3, 137.3, 170.4, 170.9.

HRMS (ESI-TOF) *m/z*: Calculated for $C_{20}H_{23}ClN_3O_4$ [M+H]: 404.1377; found 404.1364.

Synthesis of *N*-(((2*R*,3*R*,4*S*)-4-(benzamidomethyl)-3,4-dihydroxy-1-(2-hydroxyethyl)pyrrolidin-2-yl)methyl)-2-chlorobenzamide (3a) (Scheme 7)

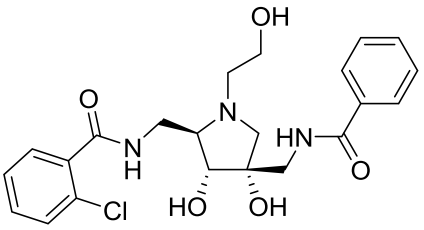

3a

To a solution of **4** (0.038 g, 0.094 mmol) in MeOH (5 mL) were added AcOH (0.108 mL, 1.88 mmol, 20.0 equiv), $NaBH_3CN$ (0.018 g, 0.282 mmol, 3.0 equiv) and glycolaldehyde dimer (0.017 g, 0.141 mmol, 1.5 equiv). The reaction was warmed to 60 °C and stirred for 2 hours, after which TLC analysis (CH_2Cl_2/MeOH/NH_4OH 84:15:1) showed full conversion of the starting material. The reaction mixture was concentrated in vacuo, the residue dissolved in MeOH, absorbed onto celite and purified by flash column chromatography (CH_2Cl_2/MeOH/NH_4OH 99:0:1 to 79:20:1) to afford **3a** (39 mg, 0.087 mmol, 93% yield) as a white solid.

^{1}H NMR (300 MHz, DMSO-d_6) δ ppm 2.29 - 2.43 (2 H, m), 2.66 (1 H, br. s.), 2.81 - 2.95 (1 H, m), 3.18 (1 H, d, *J*=10.3 Hz), 3.24 - 3.48 (5 H, m), 3.52 - 3.67 (2 H, m), 4.36 (1 H, t, *J*=5.1 Hz), 4.59 (1 H, s), 4.80 (1 H, d, *J*=6.4 Hz), 7.23 - 7.34 (1 H, m), 7.34 - 7.61 (6 H, m), 7.78 - 7.91 (2 H, m), 8.11 (1 H, br. s.), 8.25 (1 H, t, *J*=5.7 Hz).

^{13}C NMR (75 MHz, DMSO-d_6) δ ppm 38.7, 45.9, 54.2, 56.4, 59.4, 61.8, 62.8, 67.9, 74.1, 76.3, 126.9, 127.3, 128.2, 129.0, 129.5, 129.9, 130.6, 131.2, 134.4, 137.0, 166.4, 167.0.

HRMS (ESI-TOF) *m/z*: Calculated for $C_{22}H_{27}ClN_3O_5$ [M+H]: 448.1639; found 448.1626.

Synthesis of *N*-(((2*R*,3*R*,4*S*)-4-(benzamidomethyl)-1-cyclopropyl-3,4-dihydroxypyrrolidin-2-yl)methyl)-2-chlorobenzamide (3b) (Scheme 7)

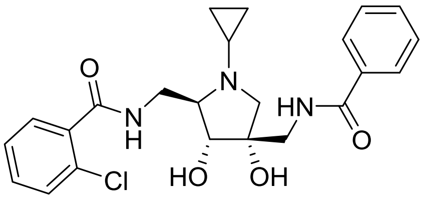

3b

To a solution of **4** (0.038 g, 0.094 mmol) in MeOH (5 mL) were added 3Å molecular sieves (500 mg), AcOH (0.108 mL, 1.88 mmol, 20.0 equiv), $NaBH_3CN$ (0.018 g, 0.282 mmol, 3.0 equiv) and (1-ethoxycyclopropoxy)trimethylsilane (0.076 mL, 0.376 mmol, 4.0 equiv). The reaction was warmed to 60 °C and stirred for 4 hours, after which TLC analysis (CH_2Cl_2/MeOH/NH_4OH 84:15:1) showed full conversion of the starting material. The reaction mixture was concentrated in vacuo, the residue dissolved in MeOH, absorbed onto celite and purified by flash column chromatography (CH_2Cl_2/MeOH/NH_4OH 99:0:1 to 79:20:1). The residue was then purified again by preparative reversed phase HPLC (MeCN/H_2O 5–100%) to afford **3b** (19 mg, 0.043 mmol, 46% yield) as a white solid.

^{1}H NMR (300 MHz, DMSO-d_6) δ ppm 0.14 - 0.66 (4 H, m), 2.53 - 2.65 (1 H, m), 2.81 (1 H, br. s.), 3.15 (1 H, d, *J*=10.5 Hz), 3.21 - 3.50 (3 H, m), 3.60 (1 H, br. s.), 3.64 - 3.87 (1 H, m), 4.69 (1 H, br. s.), 4.91 (1 H, br. s.), 7.28 - 7.58 (7 H, m), 7.78 - 7.89 (2 H, m), 8.01 (1 H, br. s.), 8.28 (1 H, t, *J*=5.4 Hz).

^{13}C NMR (75 MHz, DMSO-d_6) δ ppm 3.3, 7.0, 35.4, 46.0, 62.7, 68.7, 75.0, 75.8, 126.9, 127.3, 128.2, 129.1, 129.5, 129.9, 130.6, 131.2, 134.4, 136.9, 166.3, 167.0.

HRMS (ESI-TOF) *m/z*: Calculated for $C_{23}H_{27}ClN_3O_4$ [M+H]: 444.1690; found 444.1702.

Synthesis of *N*-(((2*R*,3*R*,4*S*)-4-(benzamidomethyl)-3,4-dihydroxy-1-(oxetan-3-yl)pyrrolidin-2-yl)methyl)-2-chlorobenzamide (3c) (Scheme 7)

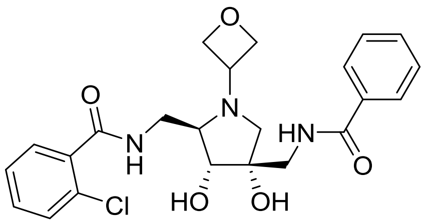

3c

To a solution of **4** (0.033 g, 0.082 mmol) in MeOH (5 mL) were added AcOH (0.094 mL, 1.64 mmol, 20.0 equiv), $NaBH_3CN$ (0.015 g, 0.246 mmol, 3.0 equiv) and 3-oxetanone (0.020 mL, 0.327 mmol, 4.0 equiv). The reaction was warmed to 60°C and stirred for 2 hours, after which TLC analysis (CH_2Cl_2/MeOH/NH_4OH 84:15:1) showed full conversion of the starting material. The reaction mixture was concentrated in vacuo, the residue dissolved in MeOH, absorbed onto celite and purified by flash column chromatography (CH_2Cl_2/MeOH/NH_4OH 99:0:1 to 79:20:1) to afford **3c** (31 mg, 0.067 mmol, 83% yield) as a white solid.

^{1}H NMR (300 MHz, DMSO-d_6) δ ppm 2.58 - 2.67 (1 H, m), 2.71 (1 H, d, *J*=10.8 Hz), 3.12 (1 H, d, *J*=10.5 Hz), 3.22 (1 H, dt, *J*=13.9, 4.8 Hz), 3.36 - 3.55 (3 H, m), 3.63 (1 H, t, *J*=7.2 Hz), 4.07 (1 H, dt, *J*=13.5, 6.4 Hz), 4.42 - 4.69 (5 H, m), 4.84 (1 H, d, *J*=6.7 Hz), 7.22 - 7.59 (7 H, m), 7.75 - 7.93 (2 H, m), 8.18 (1 H, t, *J*=5.1 Hz), 8.30 (1 H, t, *J*=5.7 Hz).

^{13}C NMR (75 MHz, DMSO-d_6) δ ppm 39.8, 45.5, 55.2, 56.1, 65.9, 74.0, 74.7, 75.7, 76.2, 126.9, 127.3, 128.2, 129.0, 129.5, 129.8, 130.6, 131.2, 134.4, 136.9, 166.4, 167.0.

HRMS (ESI-TOF) *m/z*: Calculated for $C_{23}H_{27}ClN_3O_5$ [M+H]: 460.1639; found 460.1634.

Synthesis of *N*-(((2*R*,3*R*,4*S*)-4-(benzamidomethyl)-3,4-dihydroxy-1-methylpyrrolidin-2-yl)methyl)-2-chlorobenzamide hydrochloride (3d) (Scheme 7)

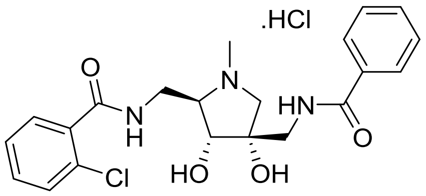

3d

A solution of **4** (0.062 g, 0.153 mmol) in THF (5 mL) was cooled to 0 °C. DIPEA (0.028 mL, 0.161 mmol, 1.05 equiv) was added, followed by dropwise addition of a solution of MeI (0.022 g, 0.158 mmol, 1.0 equiv) in THF (2 mL). The reaction mixture was stirred overnight, and concentrated in vacuo. The residue was dissolved in MeOH, absorbed onto celite and purified by flash column chromatography (CH_2Cl_2/MeOH/NH_4OH 99:0:1 to 79:20:1). The resulting product was redissolved in MeOH (10 mL), 1 M HCl (1 mL) was added and lyophilization overnight afforded the hydrochloride salt **3d** (36 mg, 0.079 mmol, 52% yield).as a white solid.

^{1}H NMR (300 MHz, DMSO-d_6) δ ppm 2.95 (3 H, d, *J*=4.7 Hz), 3.01 - 3.11 (1 H, m), 3.38 - 3.57 (3 H, m), 3.66 - 4.00 (4 H, m), 5.60 (1 H, br. s.), 5.79 (1 H, br. s.), 7.30 - 7.39 (1 H, m), 7.39 - 7.62 (6 H, m), 7.88 - 7.98 (2 H, m), 8.73 (1 H, t, *J*=6.2 Hz), 8.84 (1 H, t, *J*=5.6 Hz), 10.40 (1 H, br. s.).

^{13}C NMR (75 MHz, DMSO-d_6) δ ppm 37.4, 42.3, 44.0, 61.7, 69.5, 72.8, 76.9, 127.1, 127.4, 128.2, 129.1, 129.7, 129.9, 131.2, 131.4, 134.0, 135.9, 167.0, 167.1.

HRMS (ESI-TOF) *m/z*: Calculated for $C_{21}H_{25}ClN_3O_4$ [M+H]: 418.1534; found 418.1537.

Synthesis of *N*-(((2*R*,3*R*,4*S*)-4-(benzamidomethyl)-3,4-dihydroxy-1-(methylsulfonyl)pyrrolidin-2-yl)methyl)-2-chlorobenzamide (3e) (Scheme 7)

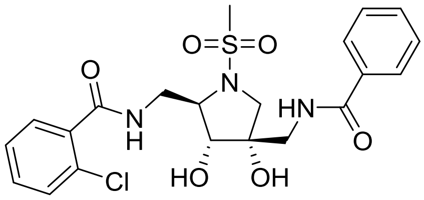

3e

A solution of **4** (0.067 g, 0.167 mmol) in THF (5 mL) was cooled to 0 °C. Et_3N (0.024 mL, 0.175 mmol, 1.05 equiv) was added, followed by dropwise addition of a solution of Ms-Cl (0.019 g, 0.167 mmol, 1.0 equiv) in THF (2 mL). The reaction mixture was stirred overnight, and concentrated in vacuo. The residue was dissolved in MeOH, absorbed onto celite and purified by flash column chromatography (0–10 % MeOH in CH_2Cl_2) to afford **3e** (28 mg, 0.058 mmol, 35% yield) as a white solid.

^{1}H NMR (300 MHz, CD_3OD) δ ppm 2.97 (3 H, s), 3.42 - 3.71 (6 H, m), 4.00 - 4.12 (2 H, m), 7.21 - 7.28 (1 H, m), 7.33 - 7.57 (6 H, m), 7.80 - 7.86 (2 H, m)

^{13}C NMR (75 MHz, CD_3OD) δ ppm 35.0, 42.1, 44.7, 57.9, 64.9, 76.3, 78.7, 128.2, 128.6, 129.7, 130.2, 131.1, 131.9, 132.3, 133.0, 135.3, 137.6, 170.7, 171.1

HRMS (ESI-TOF) *m/z*: Calculated for $C_{21}H_{25}ClN_3O_6S$ [M+H]: 482.1153; found 482.1150.

Synthesis of *N*-(((2*R*,3*R*,4*S*)-1-acetyl-4-(benzamidomethyl)-3,4-dihydroxypyrrolidin-2-yl)methyl)-2-chlorobenzamide (3f) (Scheme 7)

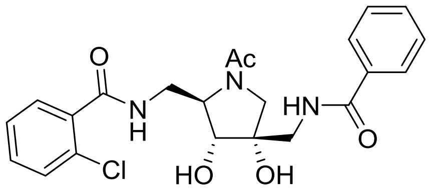

3f

To a solution of AcOH (0.063 mL, 0.11 mmol, 1.1 equiv) in DMF (2 mL) was added DIPEA (0.052 mL, 0.30 mmol, 3.0 equiv) followed by HATU (0.057 g, 0.15 mmol, 1.5 equiv). After 5 minutes of stirring, **4** (0.040 g, 0.10 mmol) was added. The yellow mixture was stirred overnight and concentrated in vacuo. The residue was dissolved in MeOH, absorbed onto celite and purified by flash column chromatography (0–10% MeOH in CH_2Cl_2 to afford **3f** (31 mg, 0.070 mmol, 70% yield) as a white powder.

^{1}H NMR (300 MHz, DMSO-d6, 80°C) δ ppm 1.91 (3 H, br. s.), 3.41 - 4.11 (6 H, m), 4.64 (1 H, br. s.), 4.89 (1 H, d, J=7.0 Hz), 7.23 - 7.61 (5 H, m), 7.80 - 7.96 (2 H, m), 8.02 - 8.39 (2 H, m).

^{13}C NMR (75 MHz, DMSO-d6, 80°C) δ ppm 21.8, 43.5, 55.7, 61.3, 74.3, 76.2, 126.5, 126.9, 127.8, 128.5, 129.2, 129.5, 130.2, 130.7, 134.2, 136.7, 166.1, 166.9, 169.5.

HRMS (ESI-TOF) *m/z*: Calculated for $C_{22}H_{25}ClN_3O_5$ [M+H]: 446.1483; found 446.1476.

3. Single crystal X-ray diffraction data

For the structure of **3a**, X-ray intensity data were collected at 100 K on a Rigaku Oxford Diffraction Supernova Dual Source (Cu at zero) diffractometer equipped with an Atlas CCD detector using ω scans and Cu Kα (λ = 1.54184 Å) radiation. The images were interpreted and integrated with the program CrysAlisPro [2]. Using Olex2 [3], the structure was solved by direct methods using the ShelXS structure solution program and refined by full-matrix least-squares on F^2 using the ShelXL program package [4,5]. Non-hydrogen atoms were anisotropically refined and the hydrogen atoms in the riding mode and isotropic temperature factors fixed at 1.2 times U(eq) of the parent atoms (1.5 times for hydroxyl groups). For **3a**, the absolute configuration was established, with chirality at C9 (*R*), C10 (*R*) and C11 (*S*), showing a refined Flack parameter of 0.015(7). Positional disorder of the chloro-phenyl ring was observed and modeled in two parts with occupancy factors of 0.8818(19) and 0.1182(19), respectively. An additional water solvent molecule was observed in the crystal packing. An extended hydrogen bond network is formed between the water solvent molecules and N-H, -N, =O and O-H functional groups.

*Crystal data for compound **3a***. $C_{22}H_{28}ClN_3O_6$, M = 465.92, orthorhombic, space group $P2_12_12_1$ (No. 19), a = 10.08079(14) Å, b = 14.04949(16) Å, c = 15.64296(17) Å, V = 2215.51(5) Å^3, Z = 4, T = 100 K, ρ_{calc} = 1.397 g cm^{-3}, μ(Cu-Kα) = 1.910 mm^{-1}, F(000) = 984, 31219 reflections measured, 4519 unique (R_{int} = 0.0579) which were used in all calculations. The final $R1$ was 0.0337 ($I > 2\sigma(I)$) and $wR2$ was 0.0883 (all data).

CCDC 1858528 contains the supplementary crystallographic data for this paper and can be obtained free of charge via www.ccdc.cam.ac.uk/conts/retrieving.html (or from the Cambridge Crystallographic Data Centre, 12, Union Road, Cambridge CB2 1EZ, UK; fax: +44-1223-336033; or deposit@ccdc.cam.ac.uk).

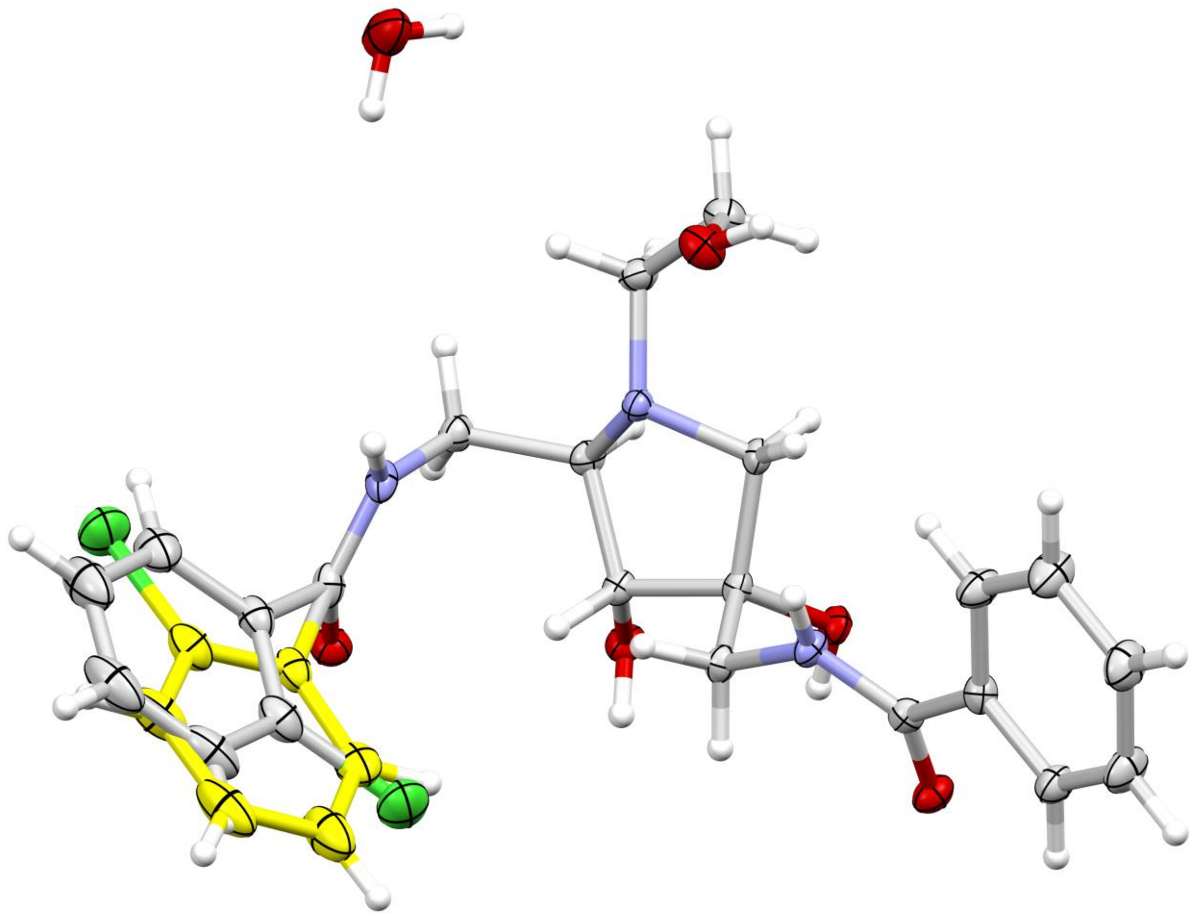

Figure S1: Asymmetric unit of the crystal structure of **3a**, showing thermal displacement ellipsoids at the 50% probability level. The positional disorder of the chlorophenyl ring is shown in yellow.

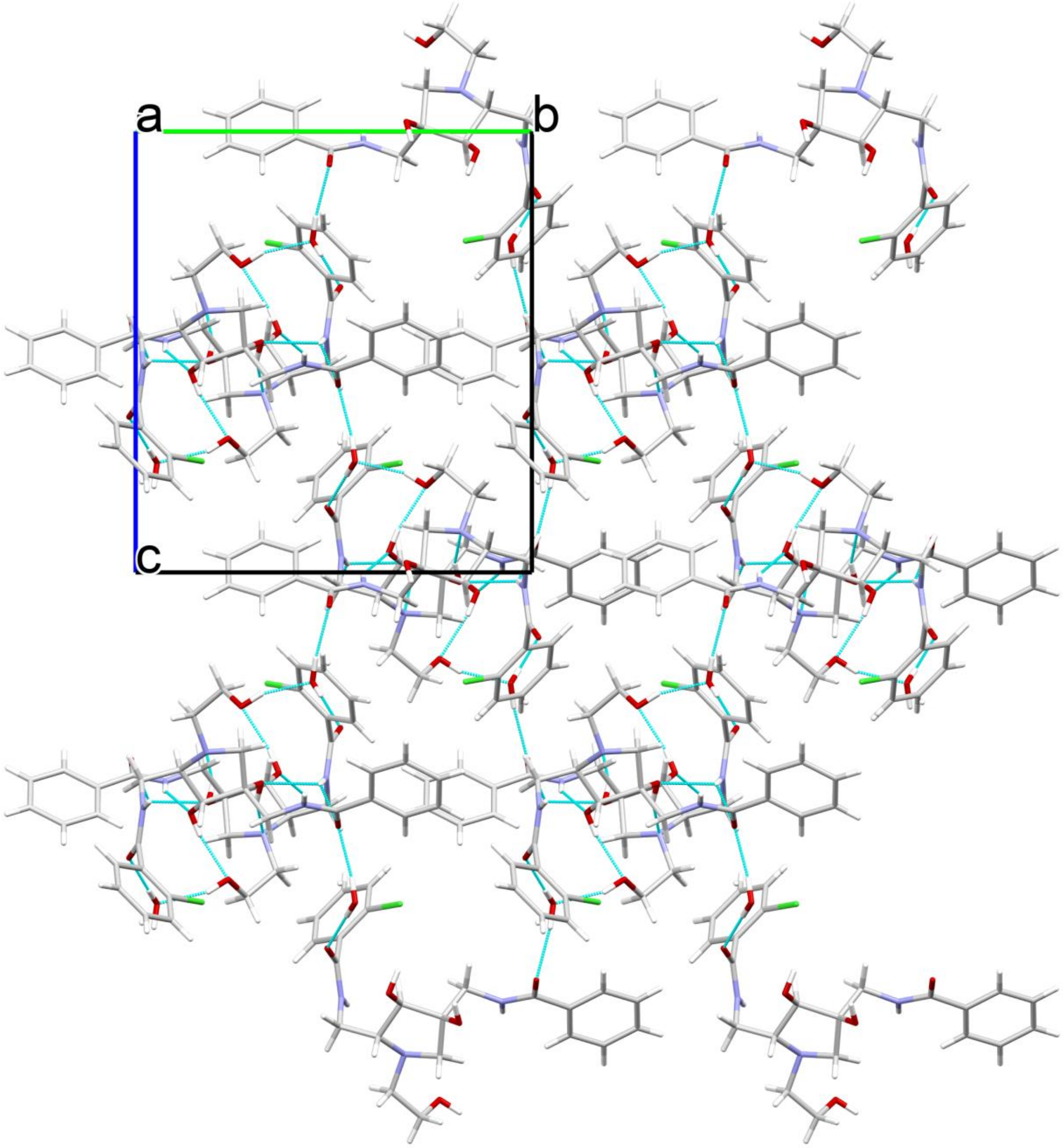

Figure S2: Packing in the crystal structure of **3a**, viewed down the a-axis, showing an extended hydrogen bond network between the water solvent molecules and N–H, –N, =O and O–H functional groups.

4. References

1. Xiong, H.; Chen, B.; Durand-Reville, T. F.; Joubran, C.; Alelyunas, Y. W.; Wu, D.; Huynh, H. *ACS Med Chem Lett* **2014,** *5*, 1143-7.

2. Rigaku Oxford Diffraction (2015). CrysAlis Pro; Rigaku Oxford Diffraction, Yarnton, England.

3. O.V. Dolomanov, L.J. Bourhis, R.J. Gildea, J.A.K. Howard, H. Puschmann, J. *Appl. Crystallogr*. **2004**, *42*, 339-341.

4. G.M. Sheldrick, *Acta Crystallogr. Sect. A64* **2008**, 112-122.

5. G.M. Sheldrick, *Acta Crystallogr. Sect. C71* **2015**, 3-8.

5. <u>Biological evaluation</u>

S. aureus (5 × 105 CFU) in 0.2 mL of LB was added to each well of a tissue culture-treated polystyrene 96-well plate. Hamamelitannin and its analogs were added to the bacteria at a final concentration of 170 µg/mL. After a 3.5 hour incubation at 37 °C, cell density was read at OD600nm. The growth medium was discarded and each well was gently washed three times with PBS to eliminate unbound bacteria. To evaluate the formation of biofilm, the remaining attached bacteria were fixed with methanol for 15 minutes. Plates were emptied and left to dry. Cells were stained with 0.8% crystal violet for 10 minutes. Excess stain was rinsed off by placing the plate under running tap water. The plates were air-dried and the dye bound to adherent cells was solubilized with 0.2 mL 2% sodium dodecyl sulfate. The OD of each well was determined at 570 nm.

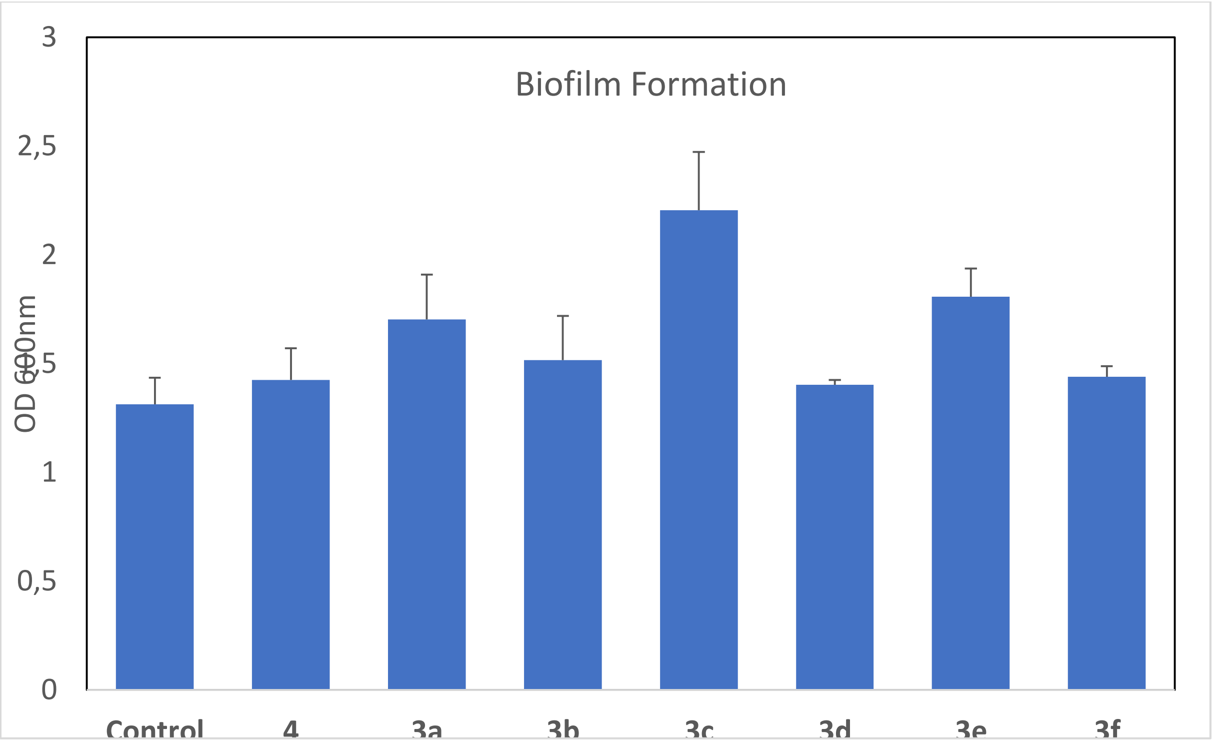

6. NMR spectra

^{1}H NMR, DMSO-*d6*, 300 MHz

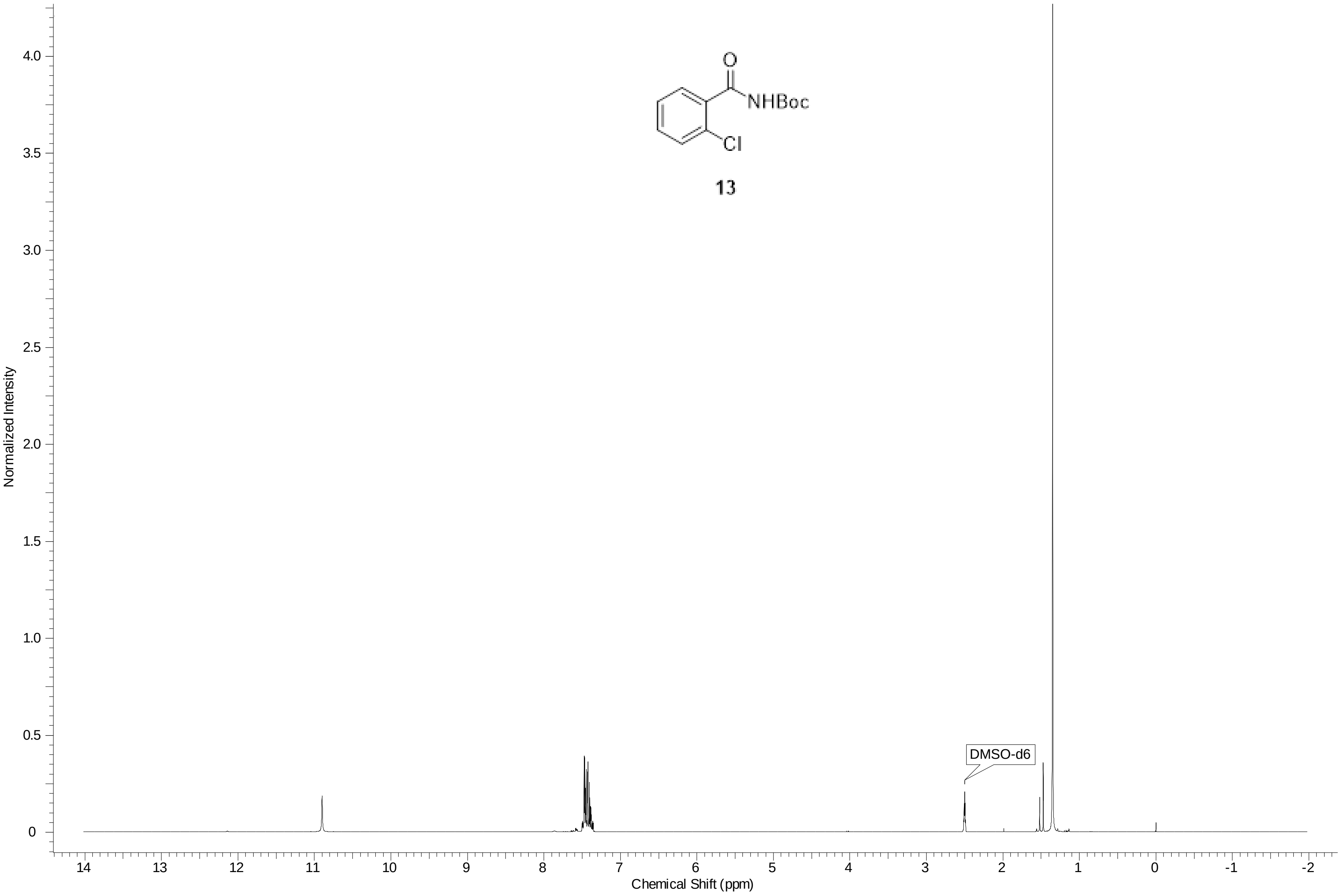

^{13}C NMR, DMSO-*d6*, 75 MHz

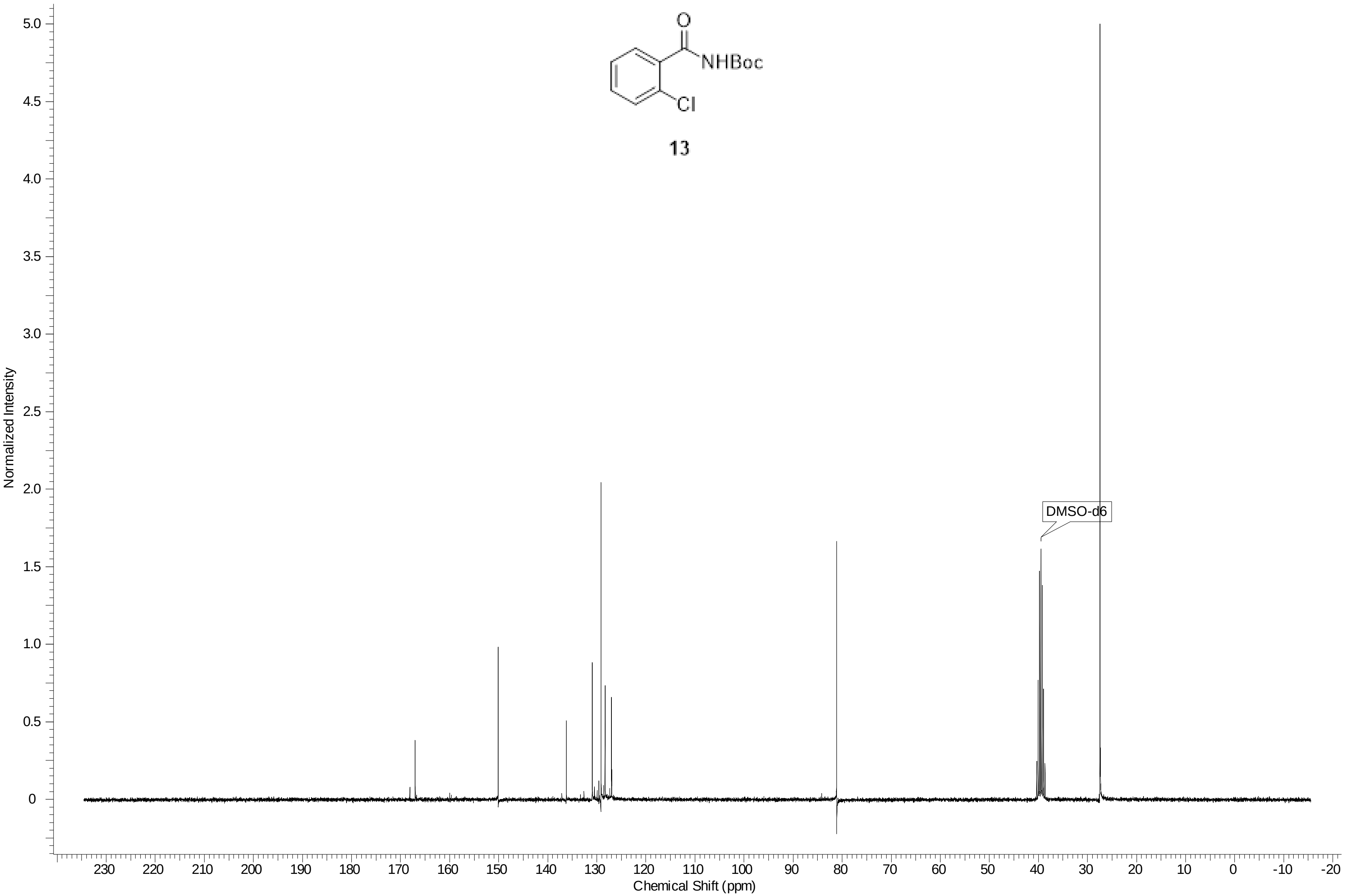

^{1}H NMR, $CDCl_3$, 300 MHz

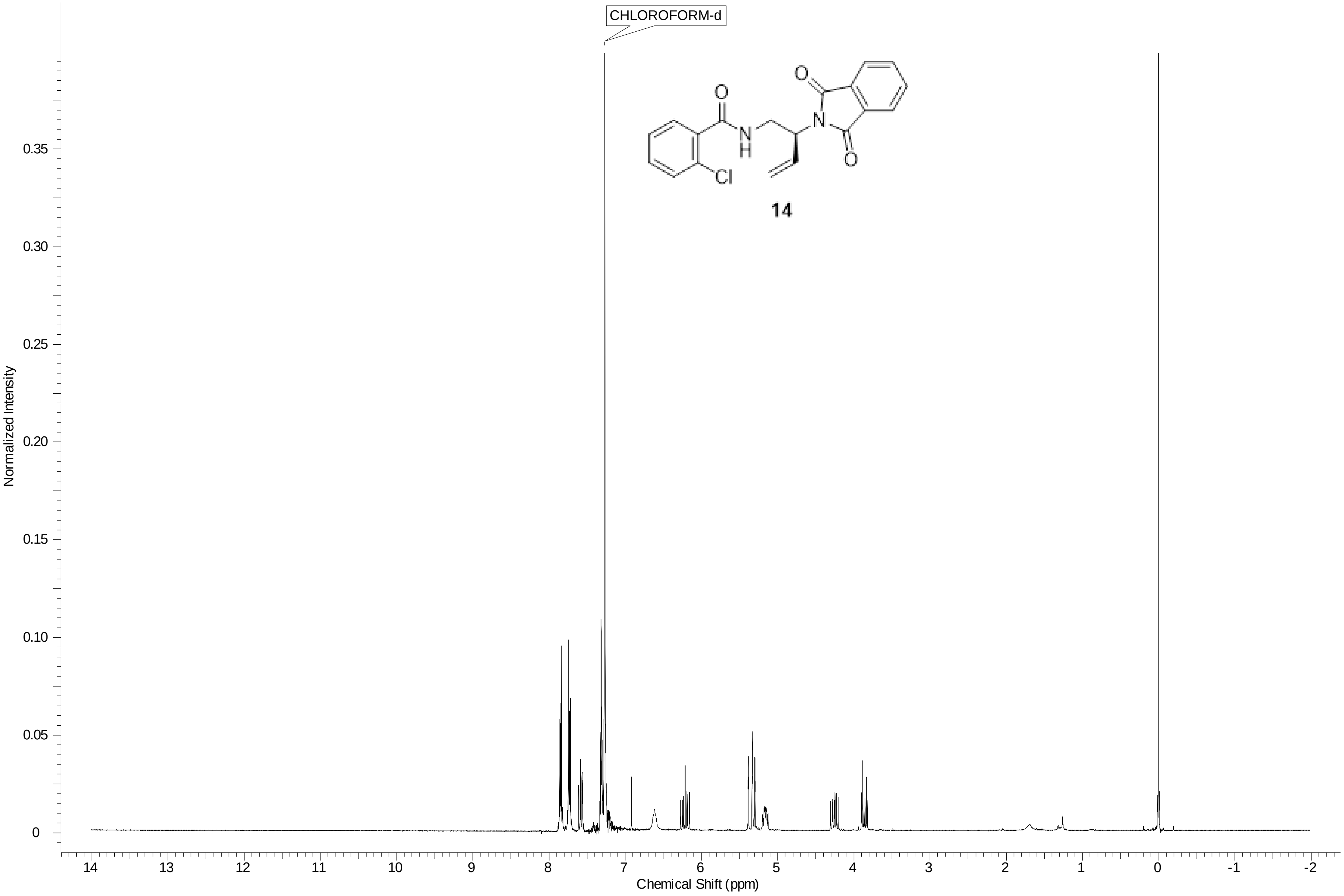

^{13}C NMR, $CDCl_3$, 75 MHz

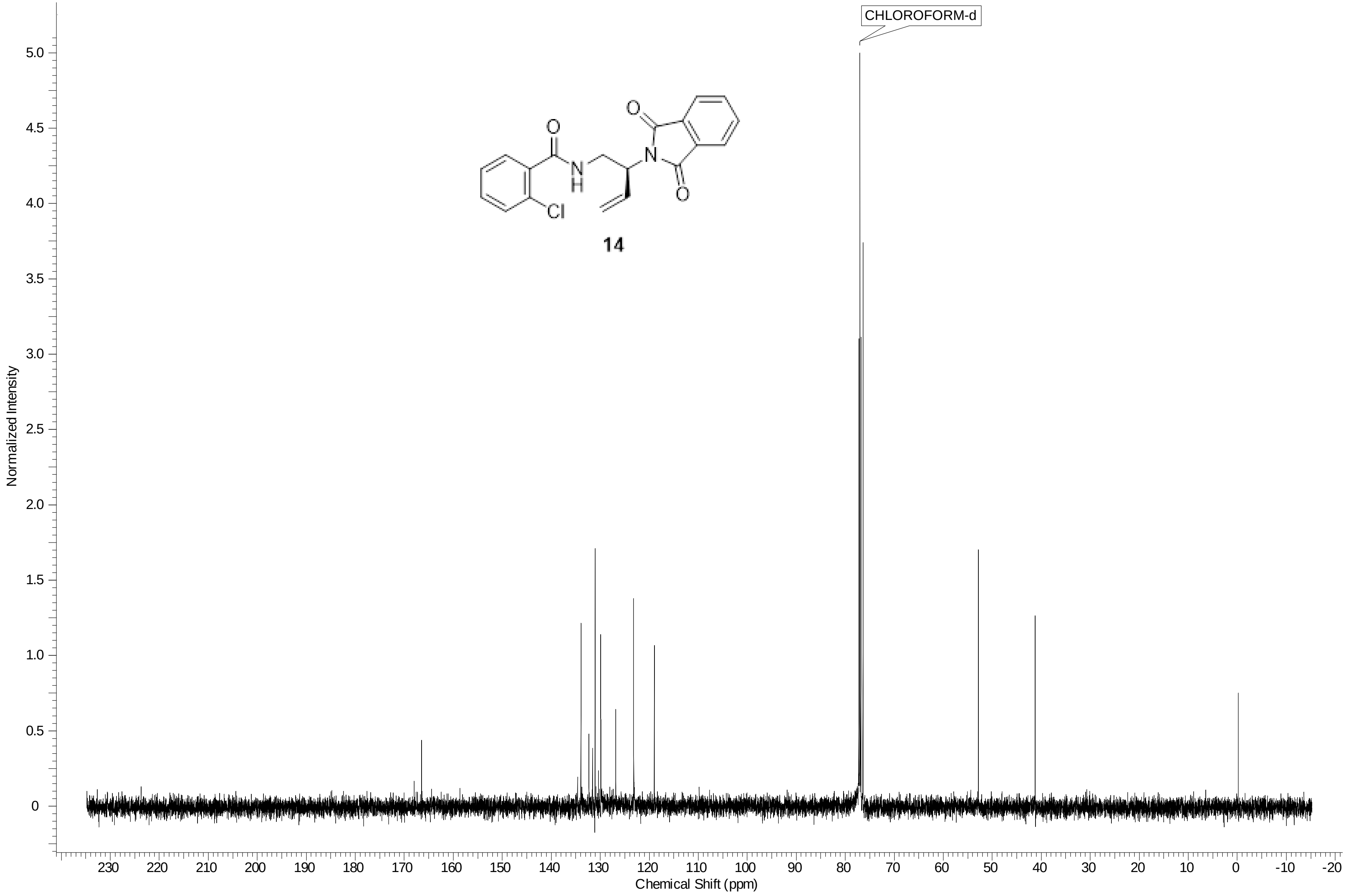

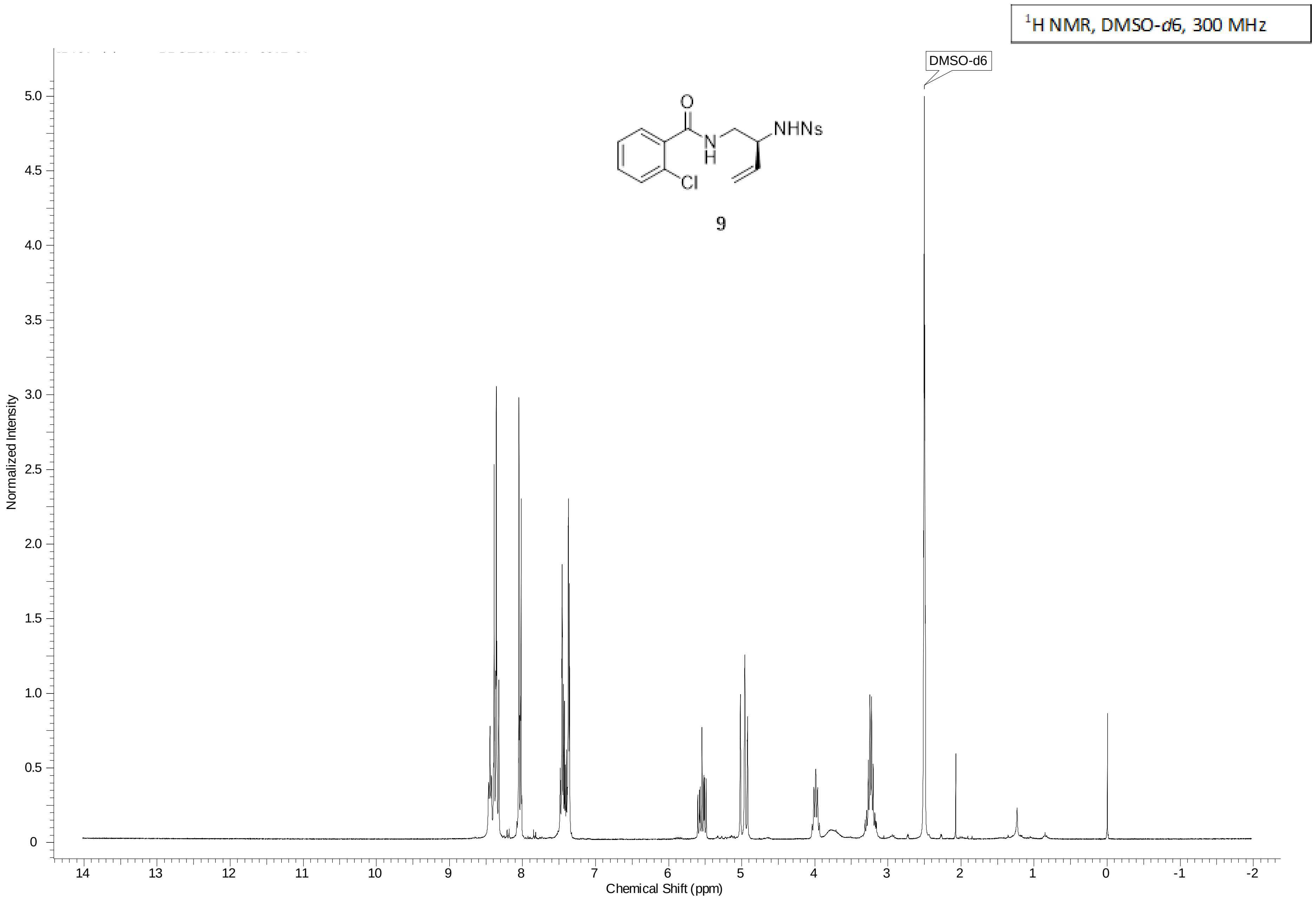

1H NMR, DMSO-d6, 300 MHz
DMSO-d6
NHNs
N
H
O
Cl
9
Normalized Intensity
Chemical Shift (ppm)

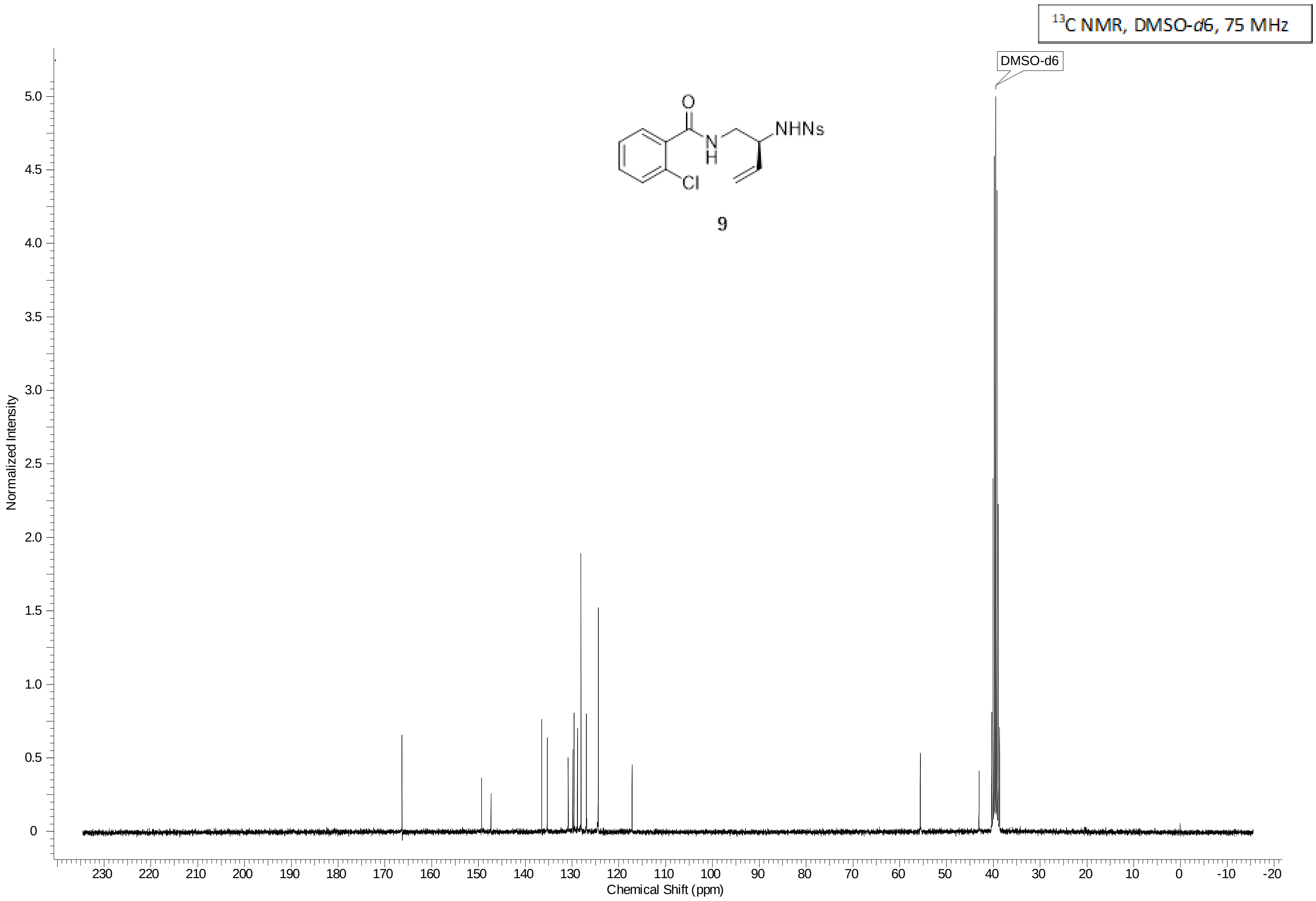

13C NMR, DMSO-d6, 75 MHz
DMSO-d6
NHNs
Cl
9
Normalized Intensity
Chemical Shift (ppm)

^{1}H NMR, $CDCl_3$, 300 MHz

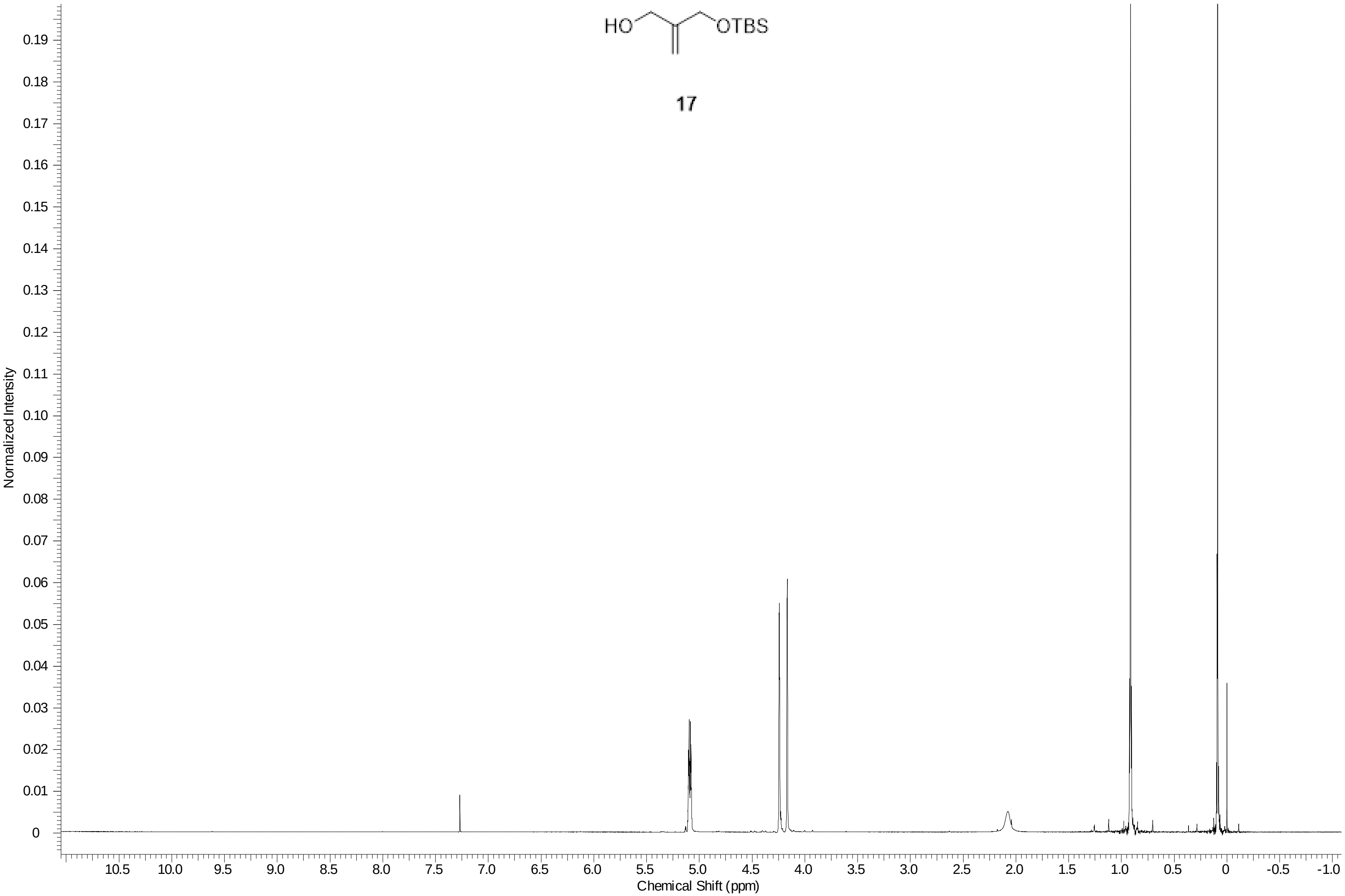

^{13}C NMR, $CDCl_3$, 75 MHz

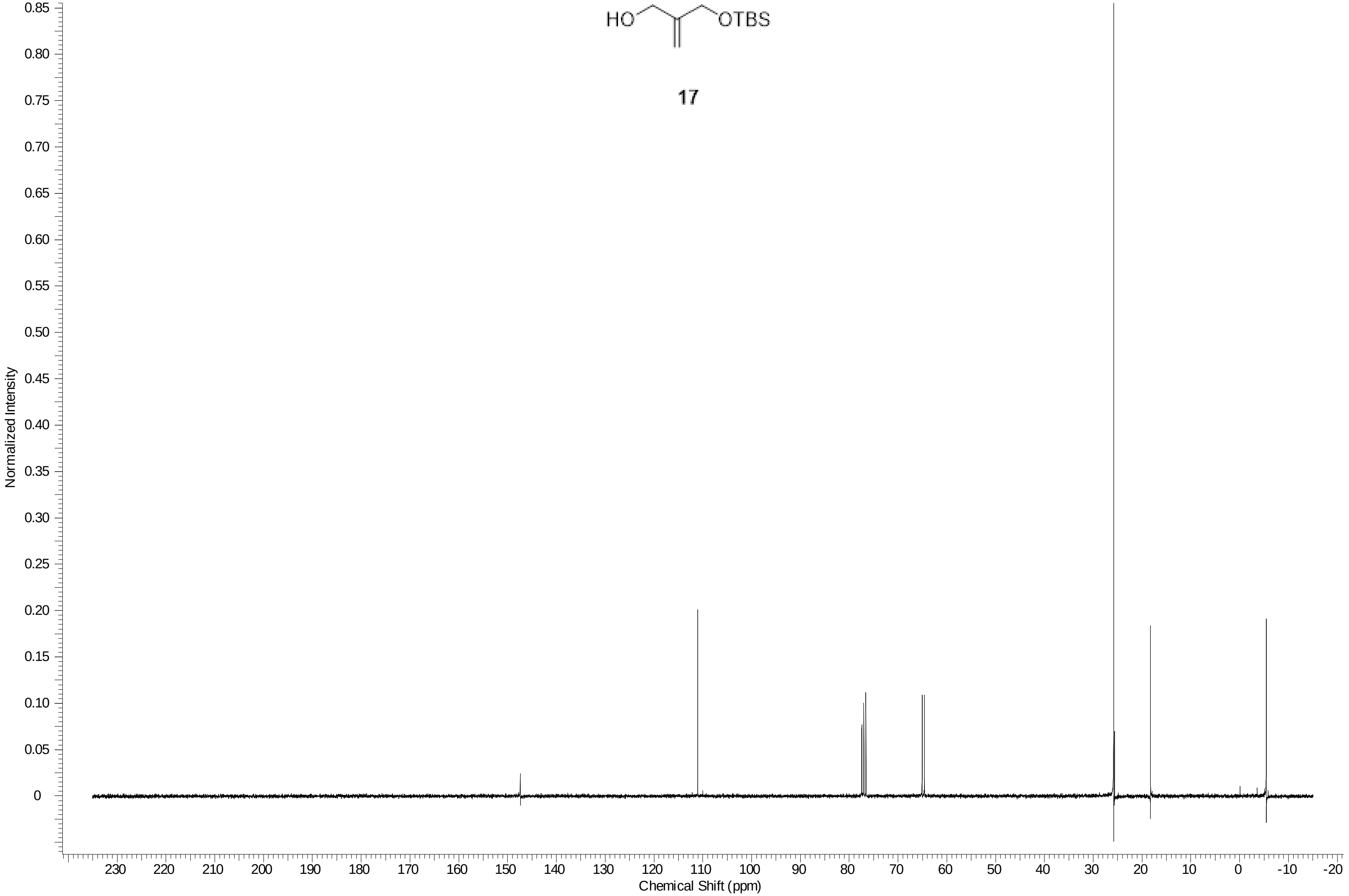

17

^{1}H NMR, $CDCl_3$, 300 MHz

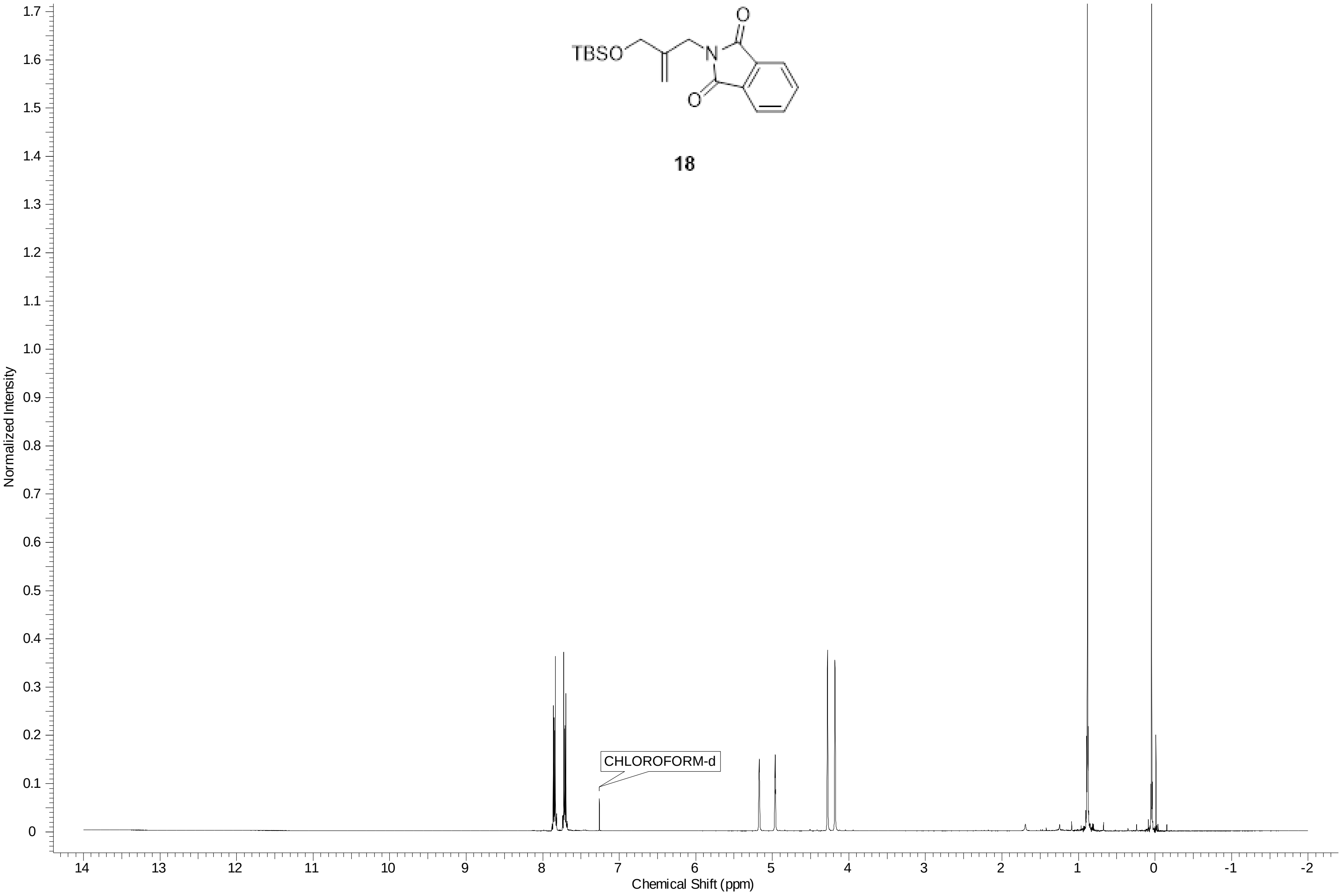

^{13}C NMR, $CDCl_3$, 75 MHz

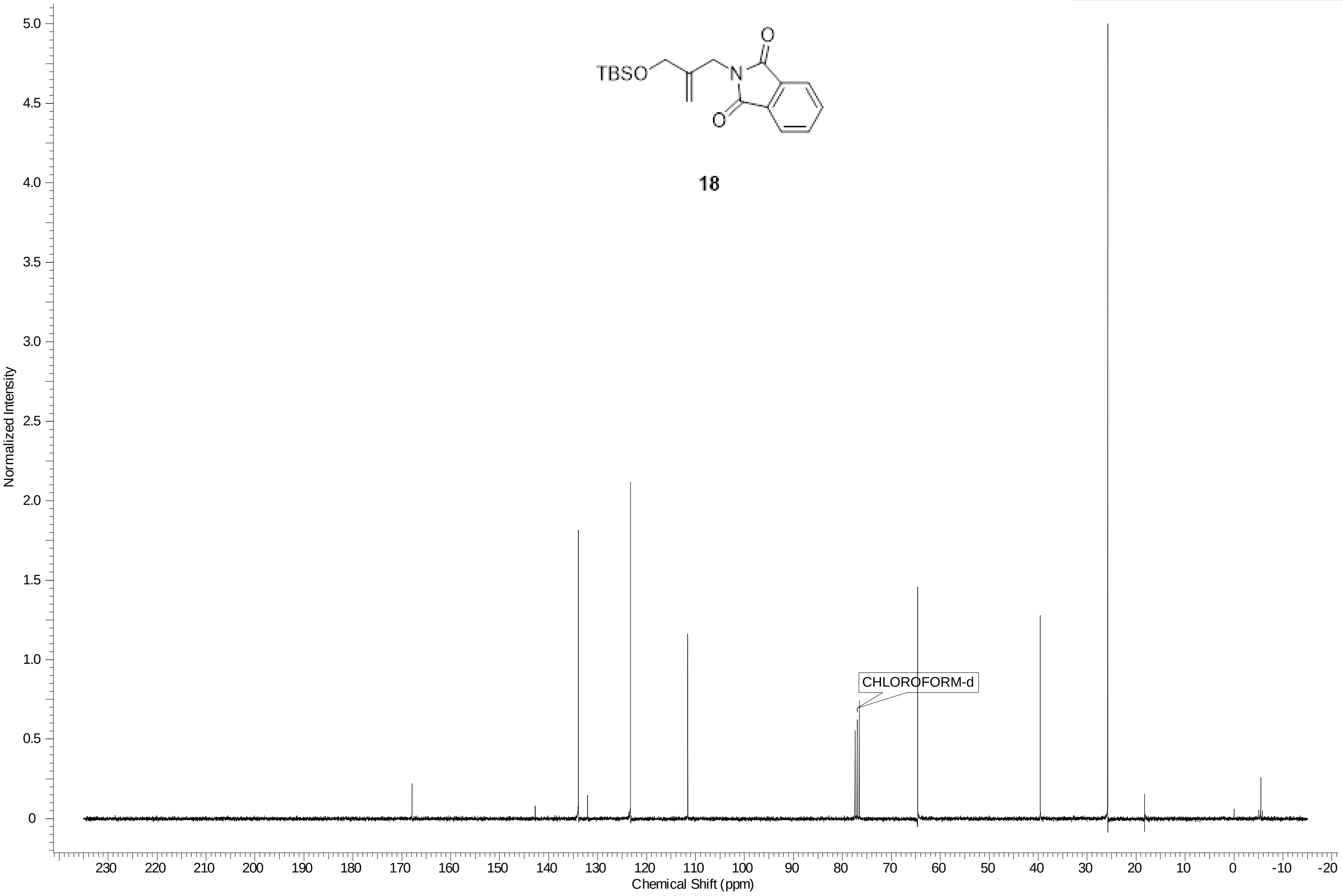

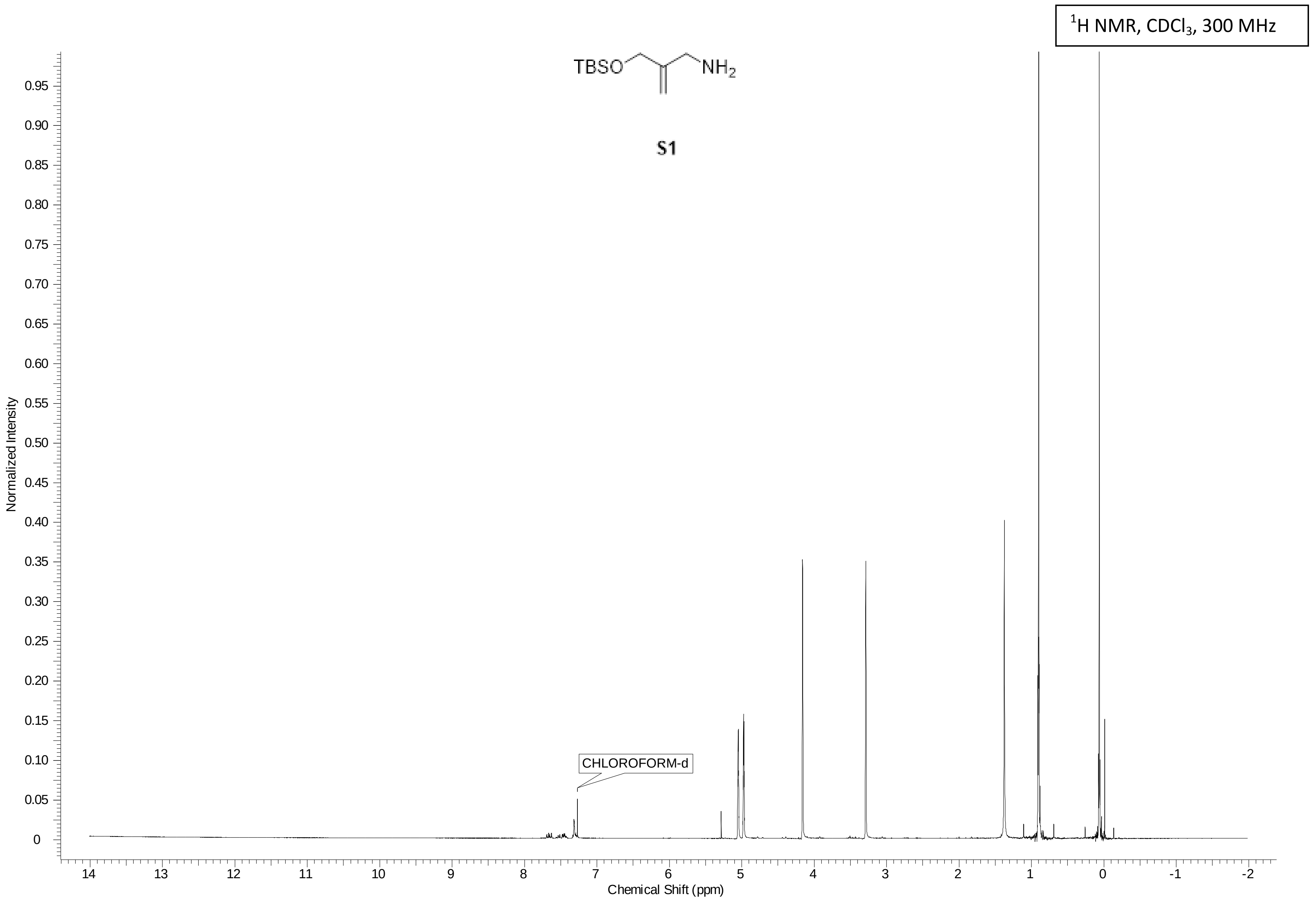

^{1}H NMR, CDCl$_3$, 300 MHz
TBSO
NH$_2$
S1
CHLOROFORM-d
Normalized Intensity
Chemical Shift (ppm)

^{13}C NMR, $CDCl_3$, 75 MHz

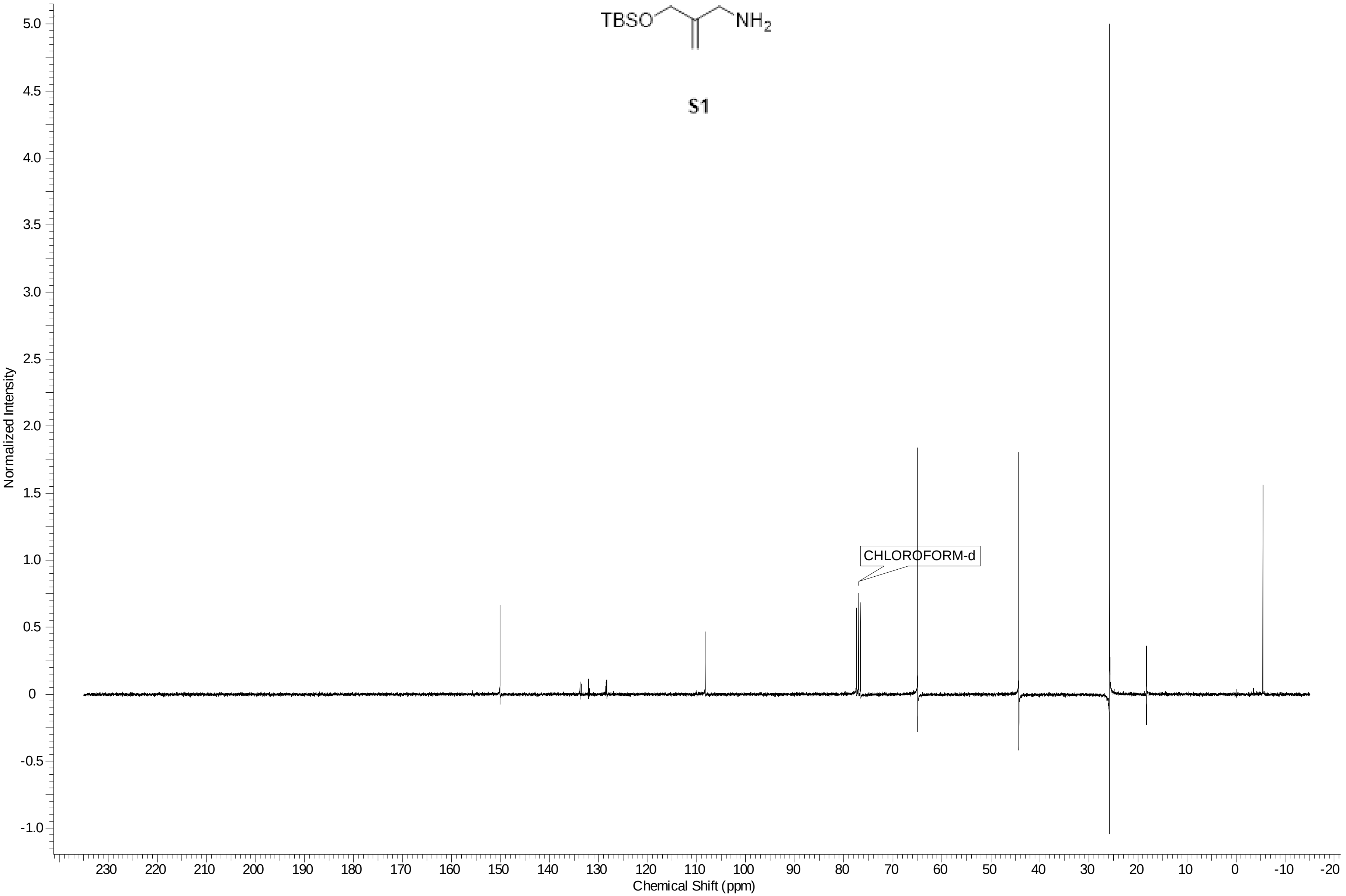

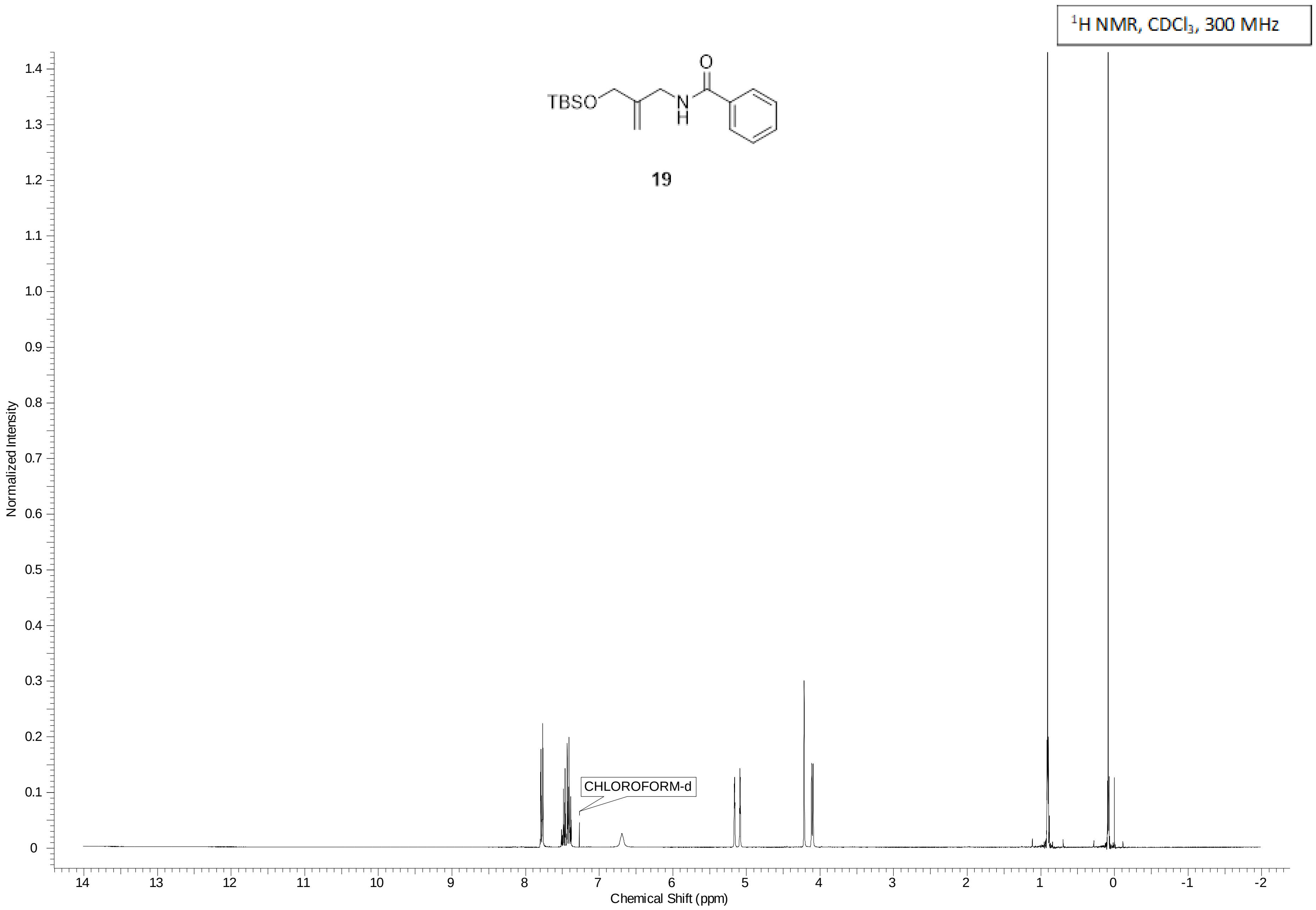

¹H NMR, CDCl₃, 300 MHz
TBSO
N
H
O
19
CHLOROFORM-d
Normalized Intensity
Chemical Shift (ppm)

^{13}C NMR, $CDCl_3$, 75 MHz

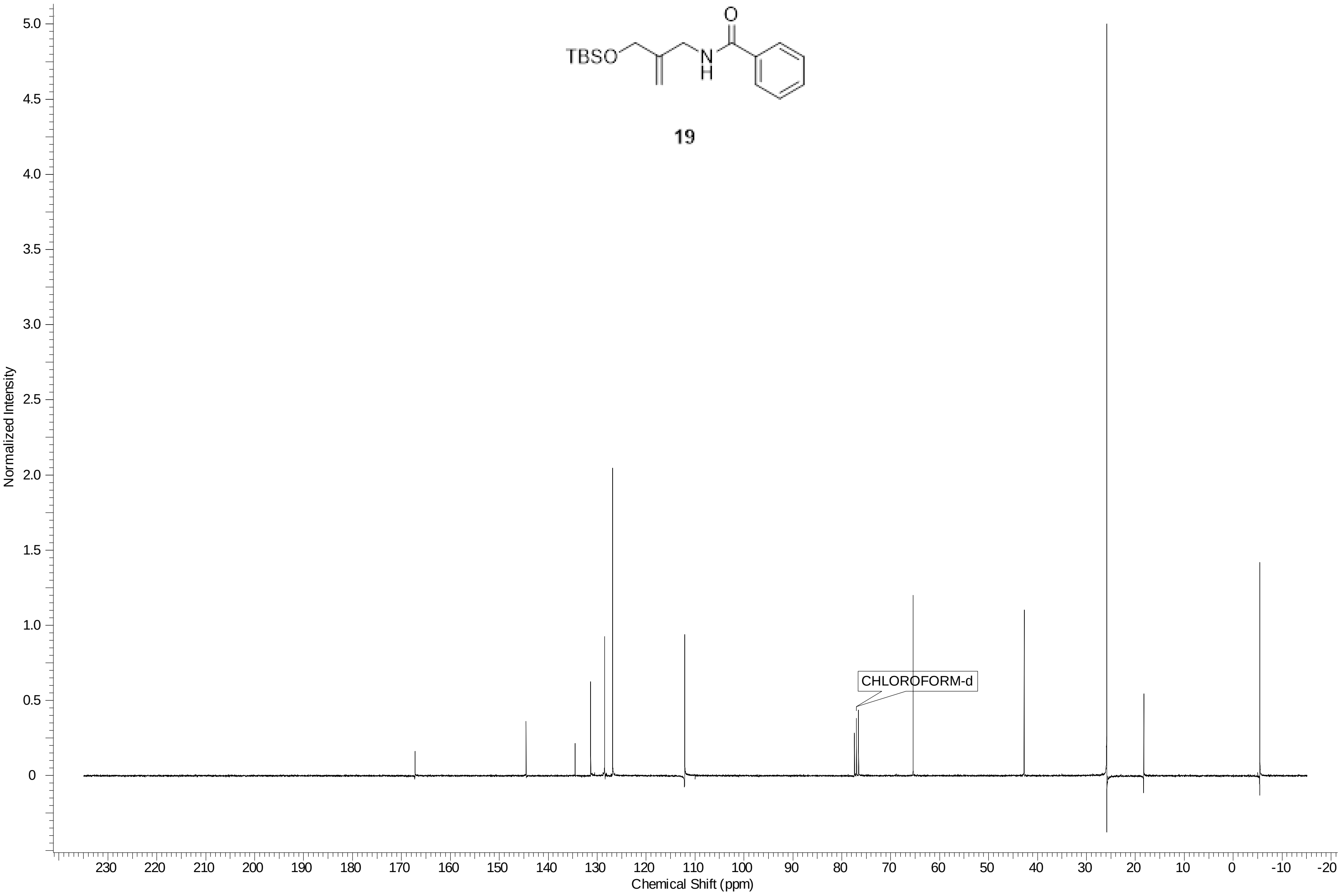

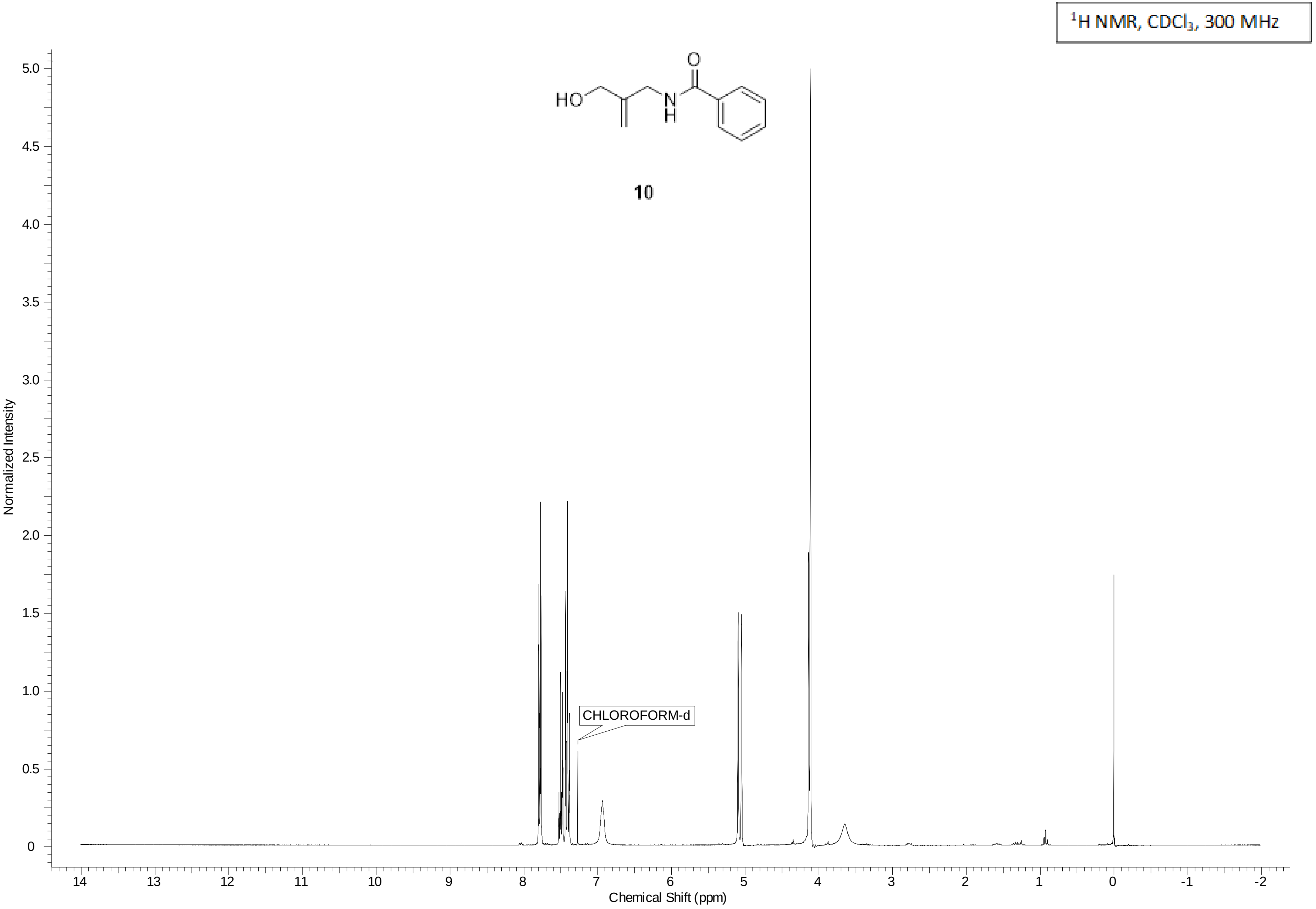

1H NMR, CDCl3, 300 MHz
HO
N
H
O
10
CHLOROFORM-d
Normalized Intensity
Chemical Shift (ppm)
5.0
4.5
4.0
3.5
3.0
2.5
2.0
1.5
1.0
0.5
0
14
13
12
11
10
9
8
7
6
5
4
3
2
1
0
-1
-2

^{13}C NMR, $CDCl_3$, 75 MHz

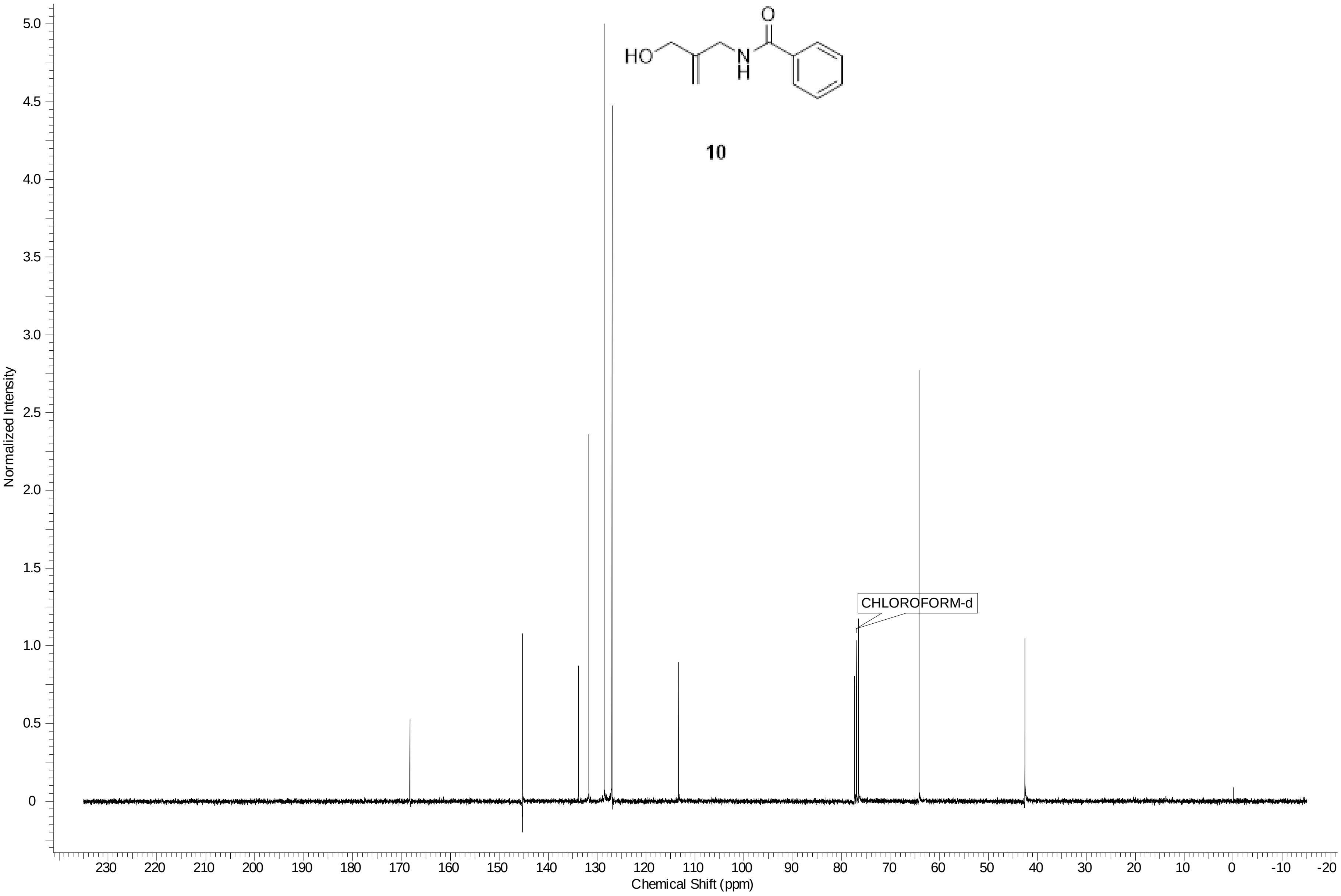

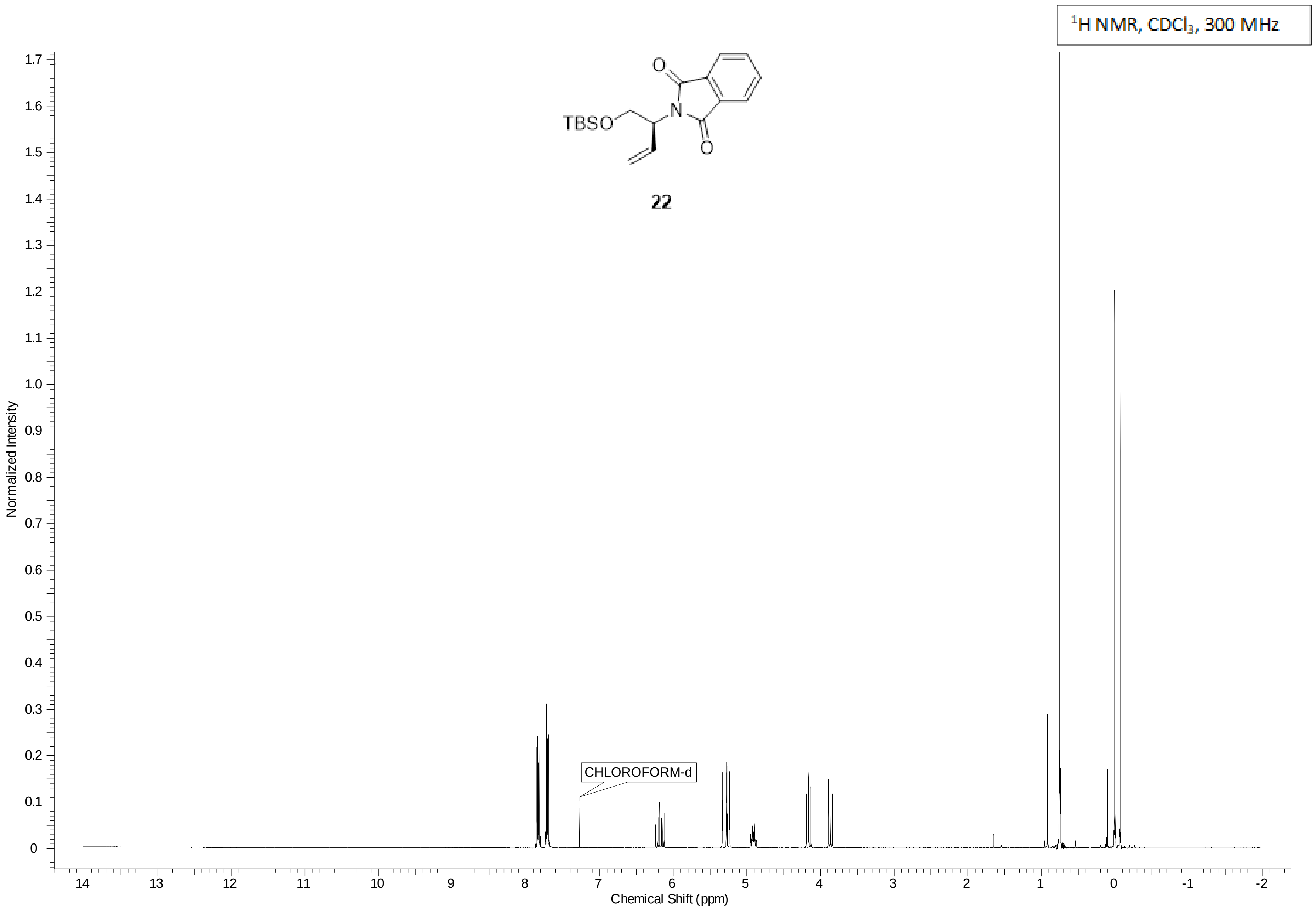

1H NMR, CDCl3, 300 MHz
TBSO
N
O
O
22
CHLOROFORM-d
Normalized Intensity
Chemical Shift (ppm)

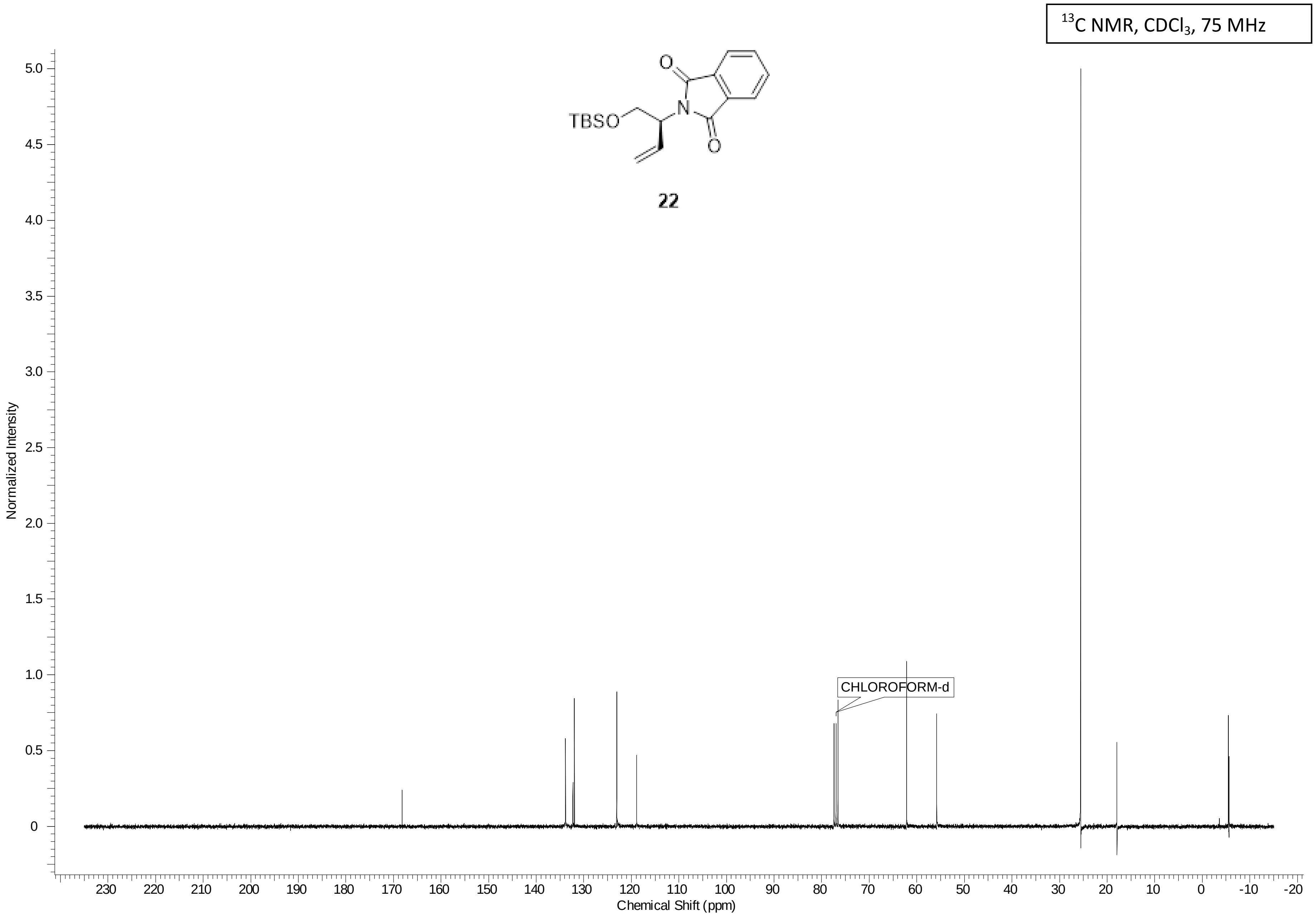
^{13}C NMR, $CDCl_3$, 75 MHz
TBSO
N
O
O
22
CHLOROFORM-d
Normalized Intensity
Chemical Shift (ppm)

1H NMR, CDCl3, 300 MHz

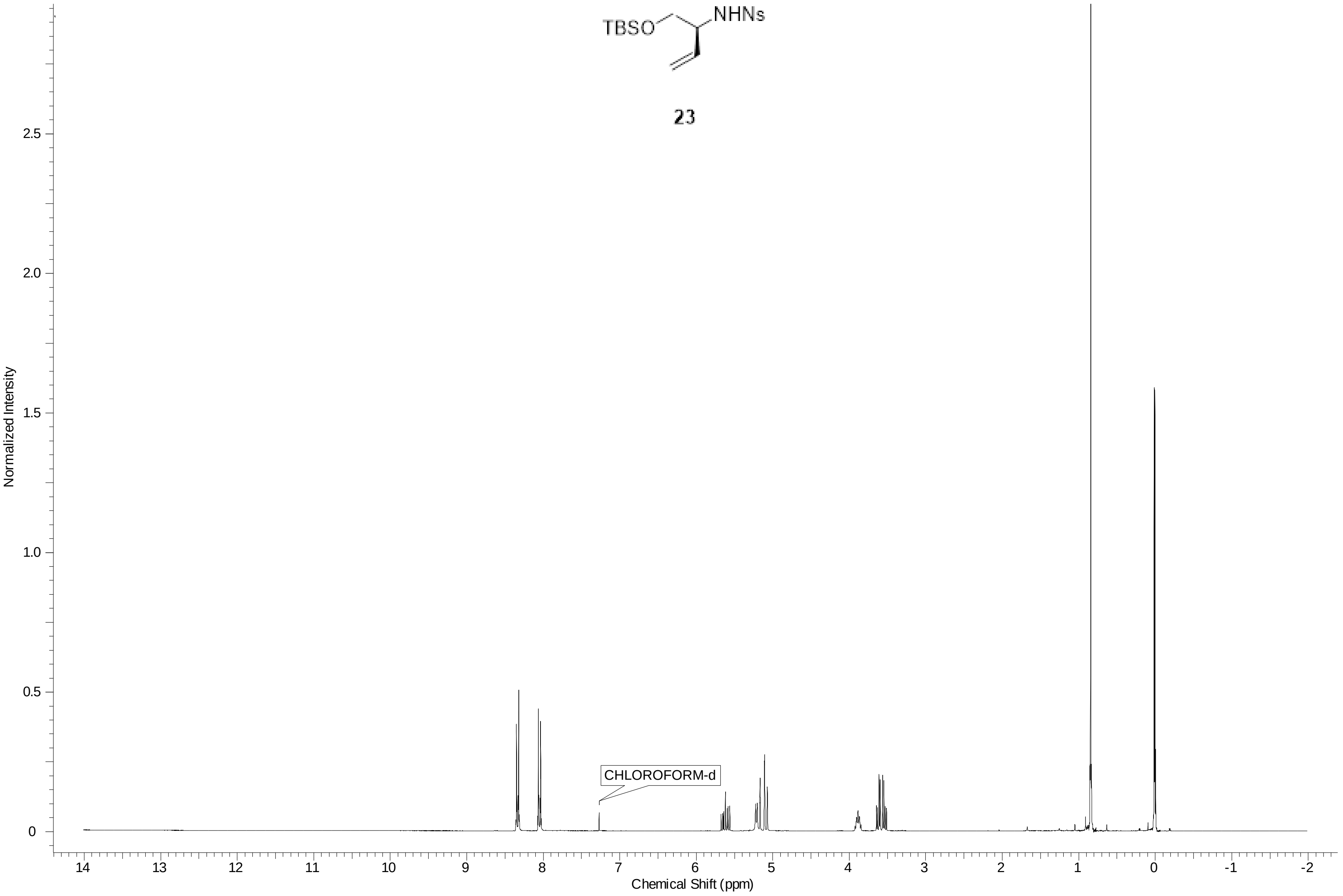
TBSO
NHNs
23
CHLOROFORM-d
Normalized Intensity
Chemical Shift (ppm)

^{13}C NMR, $CDCl_3$, 75 MHz

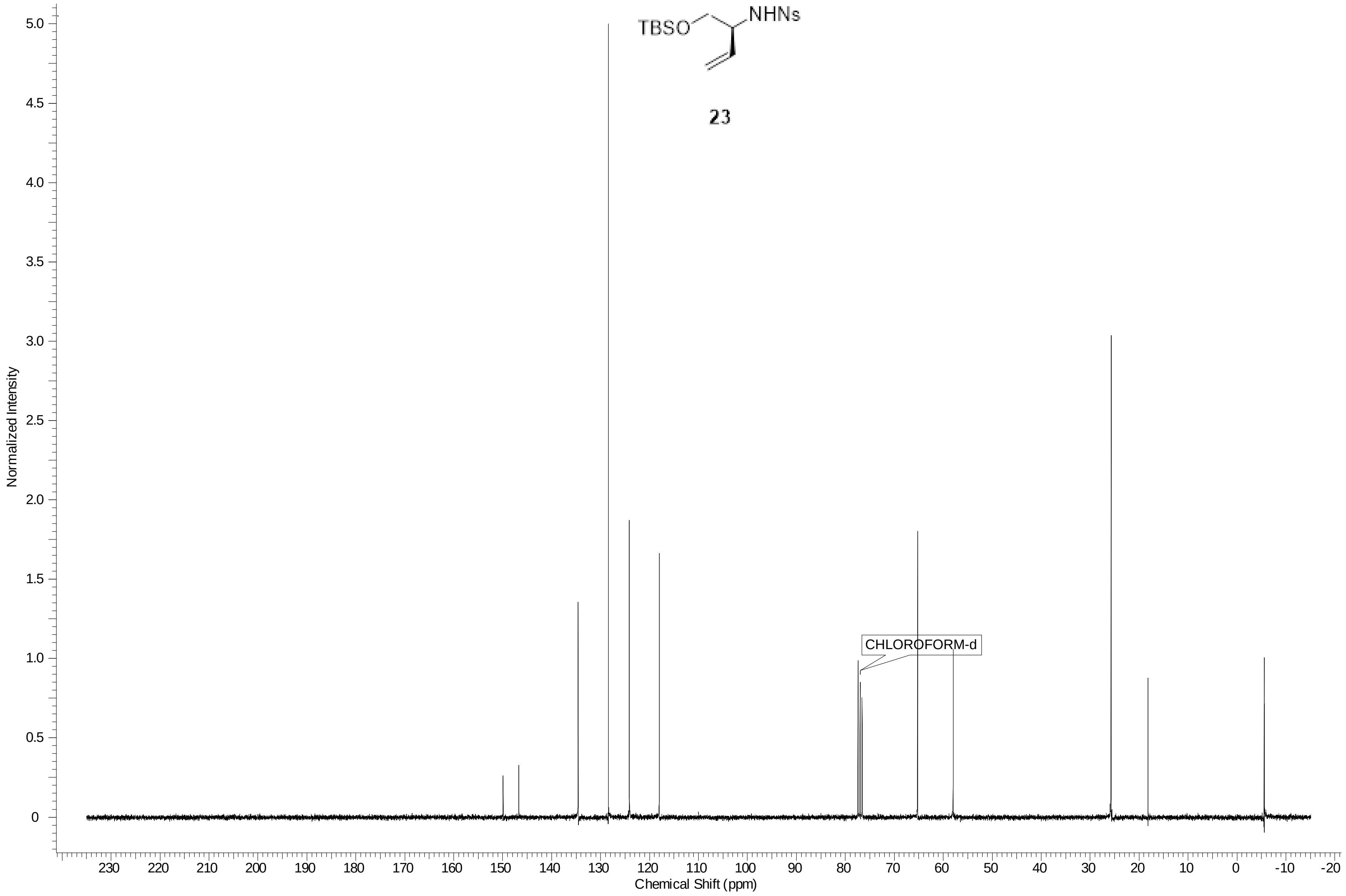

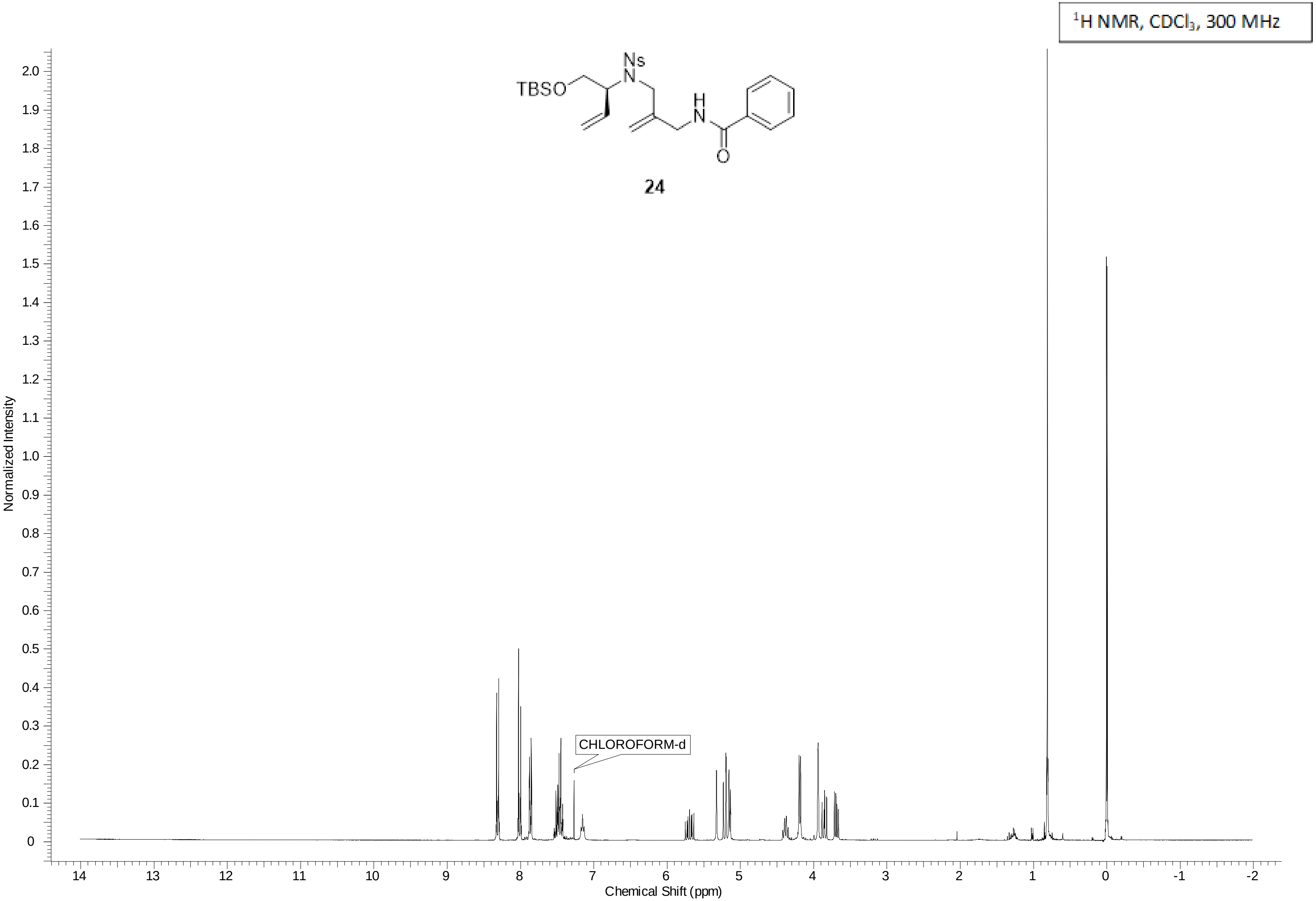
^{1}H NMR, $CDCl_3$, 300 MHz
TBSO
Ns
N
H
N
O
24
CHLOROFORM-d
Normalized Intensity
Chemical Shift (ppm)

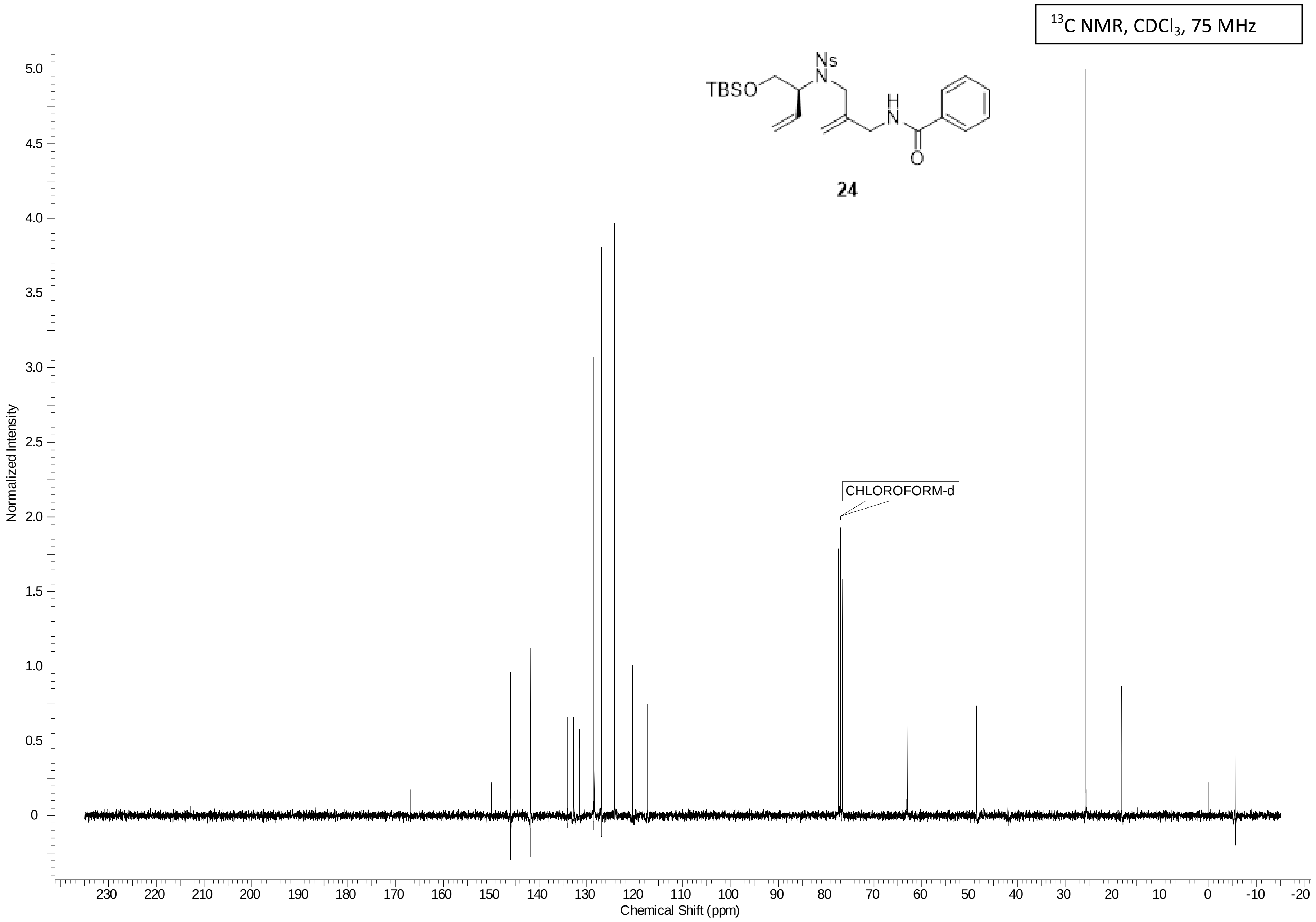
13C NMR, CDCl3, 75 MHz
TBSO
Ns
N
H
N
O
24
CHLOROFORM-d
Normalized Intensity
Chemical Shift (ppm)

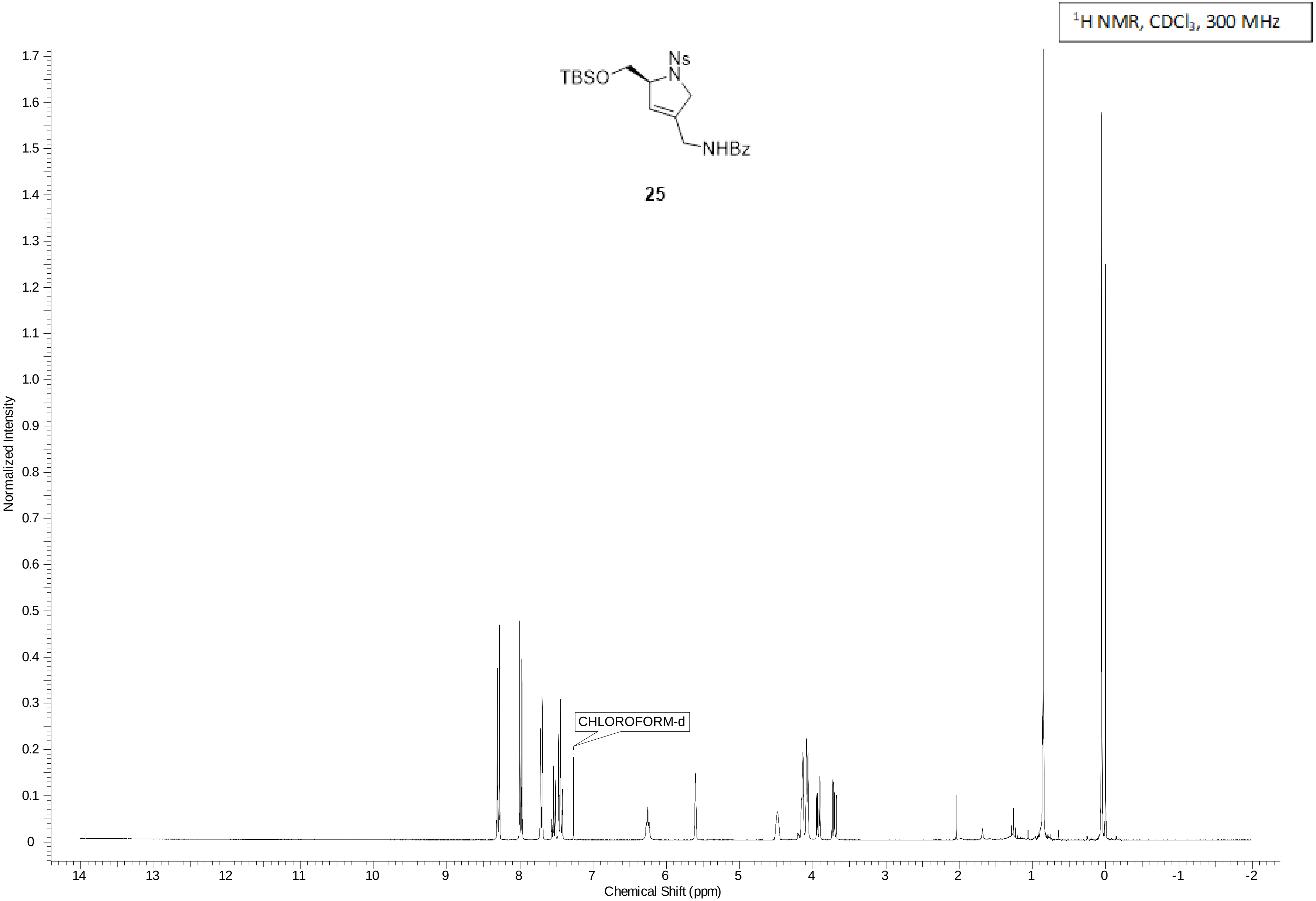

^{1}H NMR, CDCl$_3$, 300 MHz
TBSO
Ns
N
NHBz
25
CHLOROFORM-d
Normalized Intensity
Chemical Shift (ppm)

^{13}C NMR, $CDCl_3$, 75 MHz

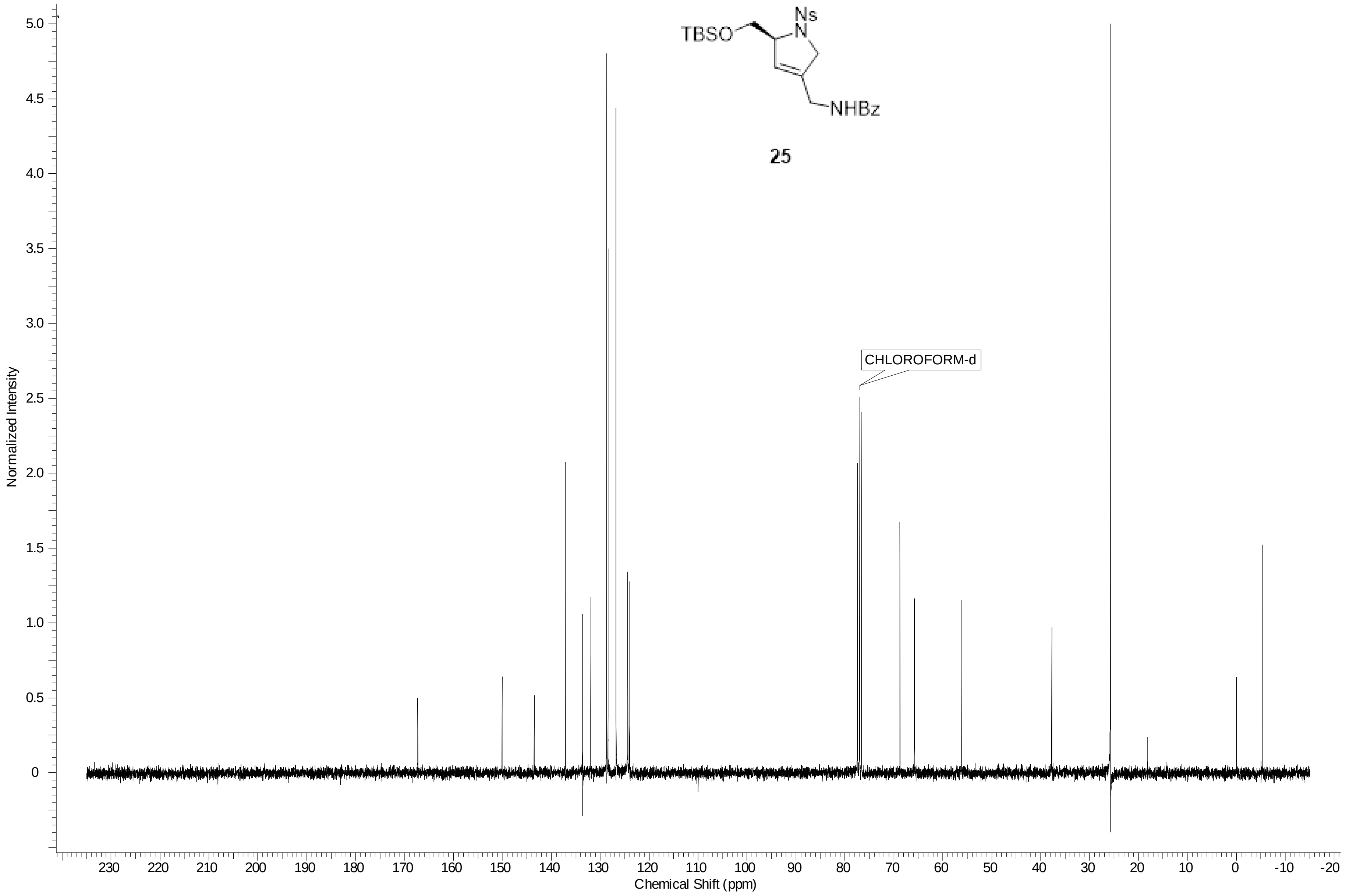

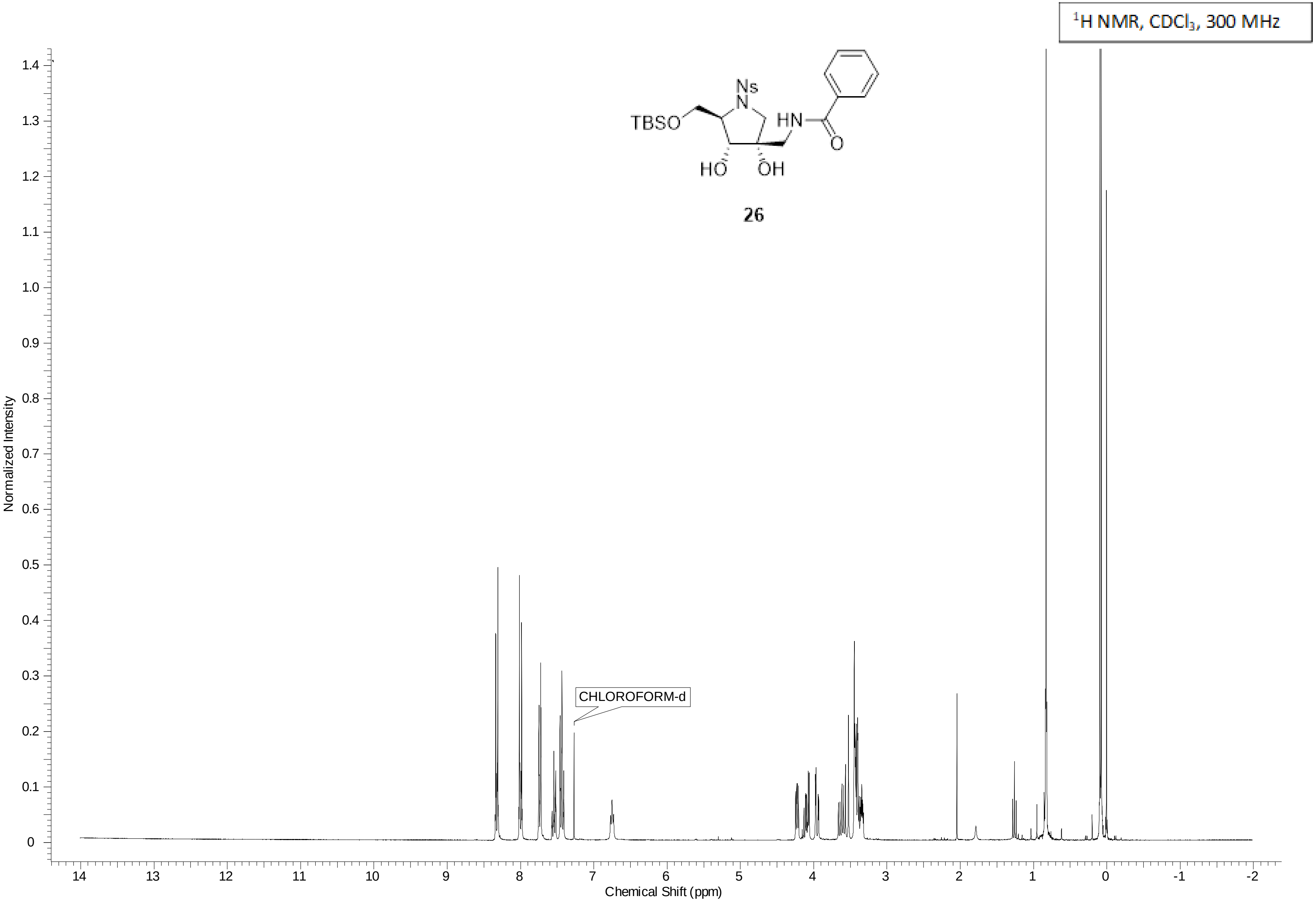

1H NMR, CDCl3, 300 MHz
Ns
N
TBSO
HN
O
HO
OH
26
CHLOROFORM-d
Normalized Intensity
Chemical Shift (ppm)

^{13}C NMR, $CDCl_3$, 75 MHz

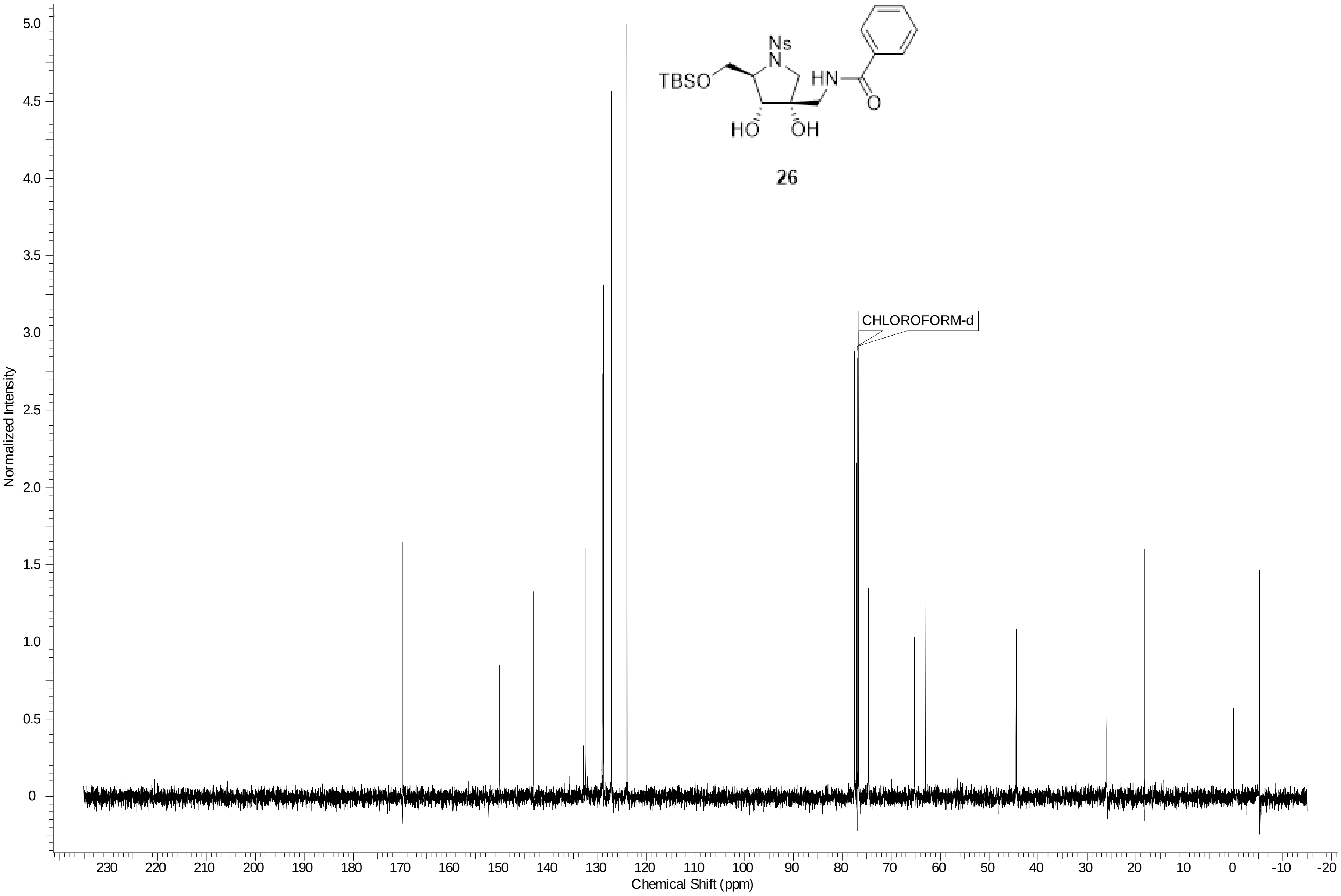

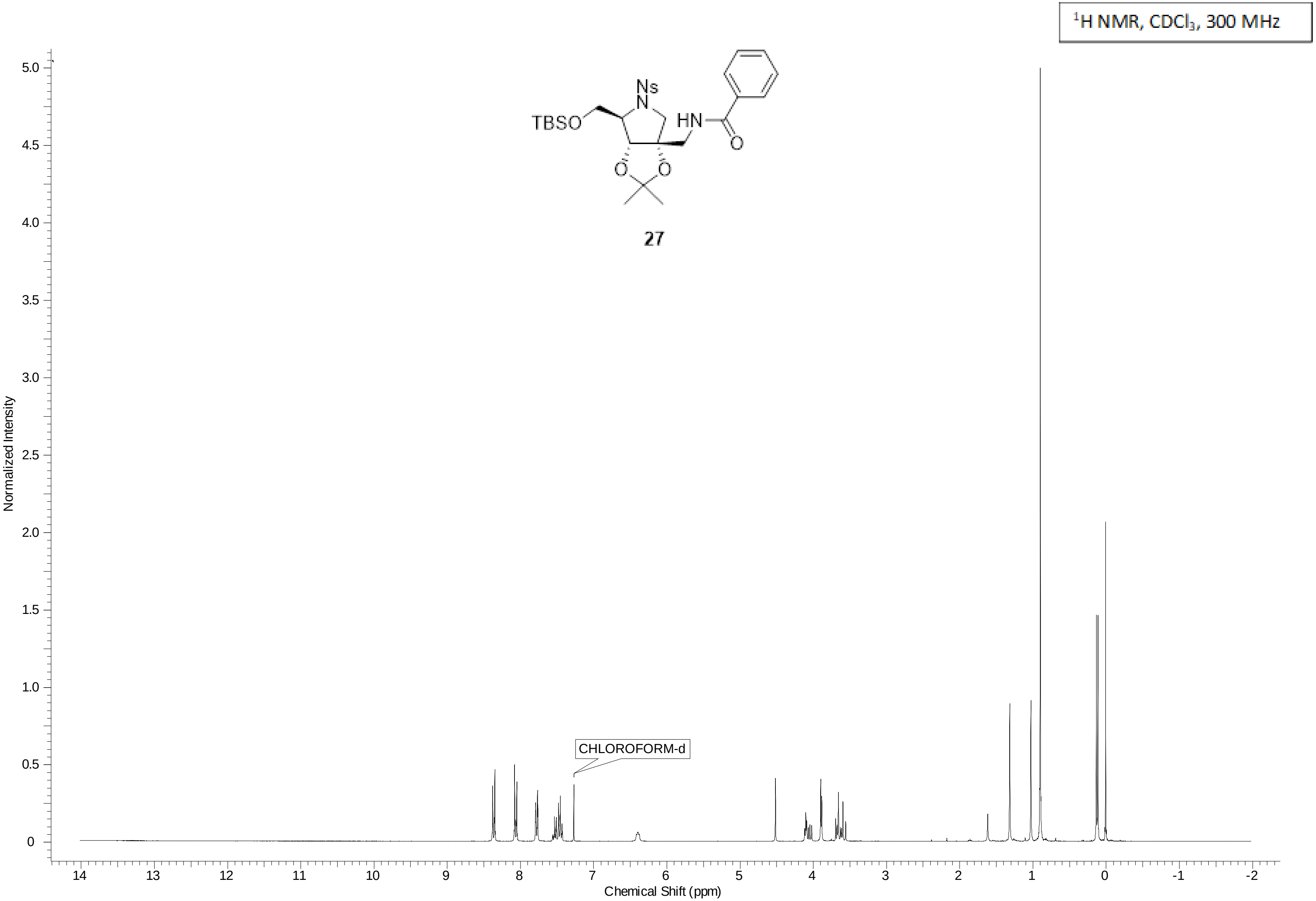

1H NMR, CDCl3, 300 MHz
TBSO
Ns
N
HN
O
27
CHLOROFORM-d
Normalized Intensity
Chemical Shift (ppm)

^{13}C NMR, $CDCl_3$, 75 MHz

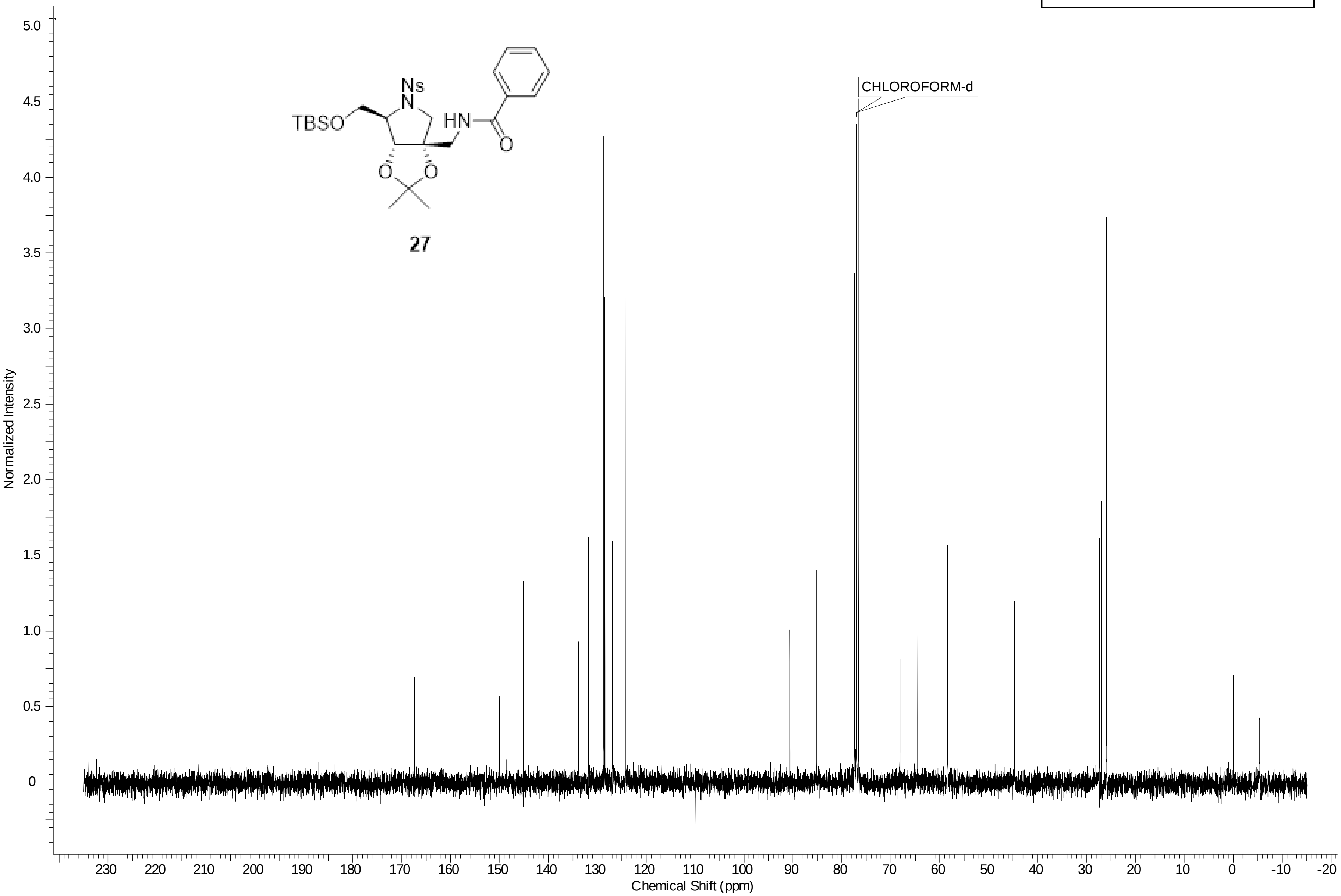

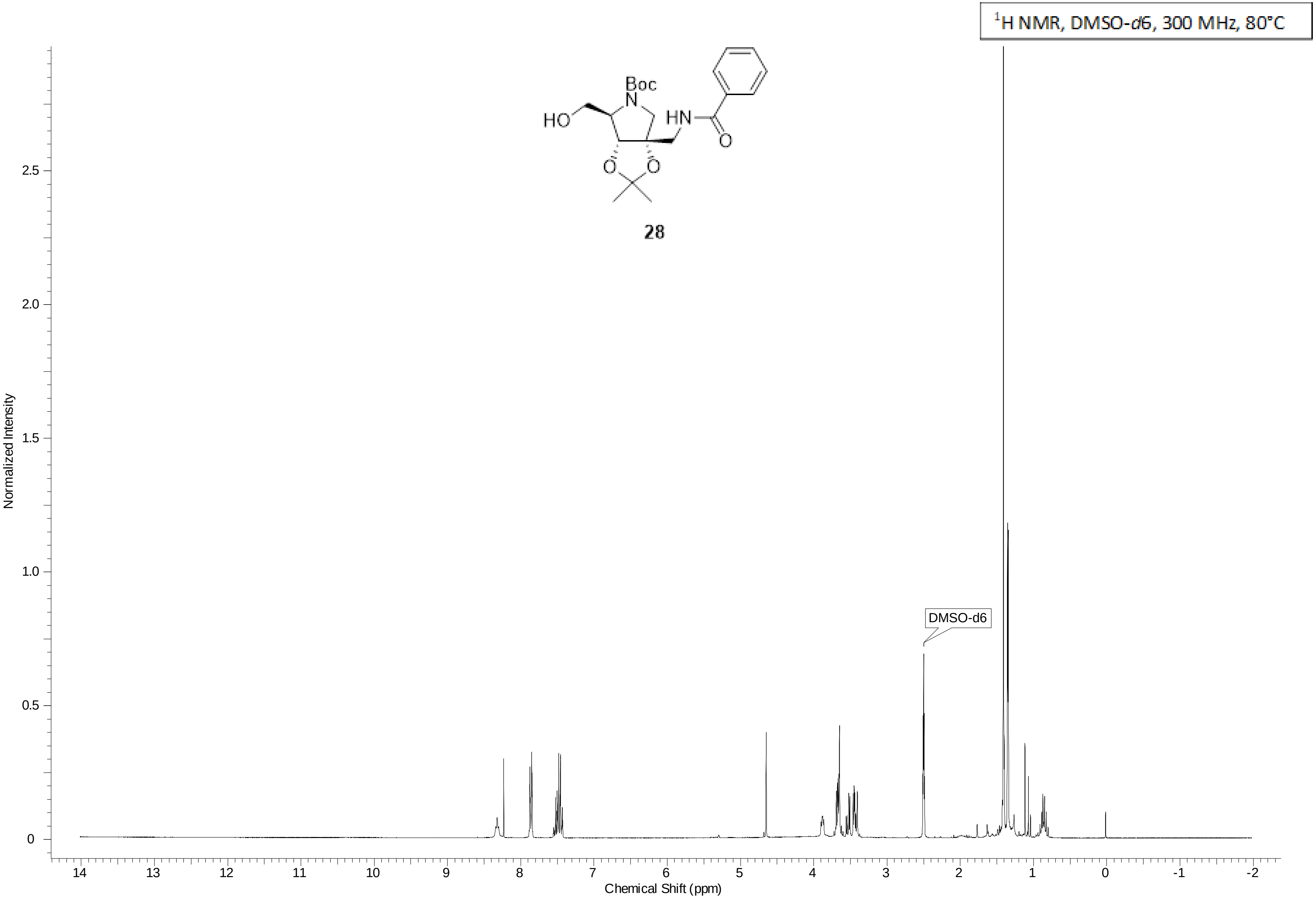

1H NMR, DMSO-d6, 300 MHz, 80°C
Boc
N
HO
HN
O
O
O
28
DMSO-d6
Normalized Intensity
Chemical Shift (ppm)

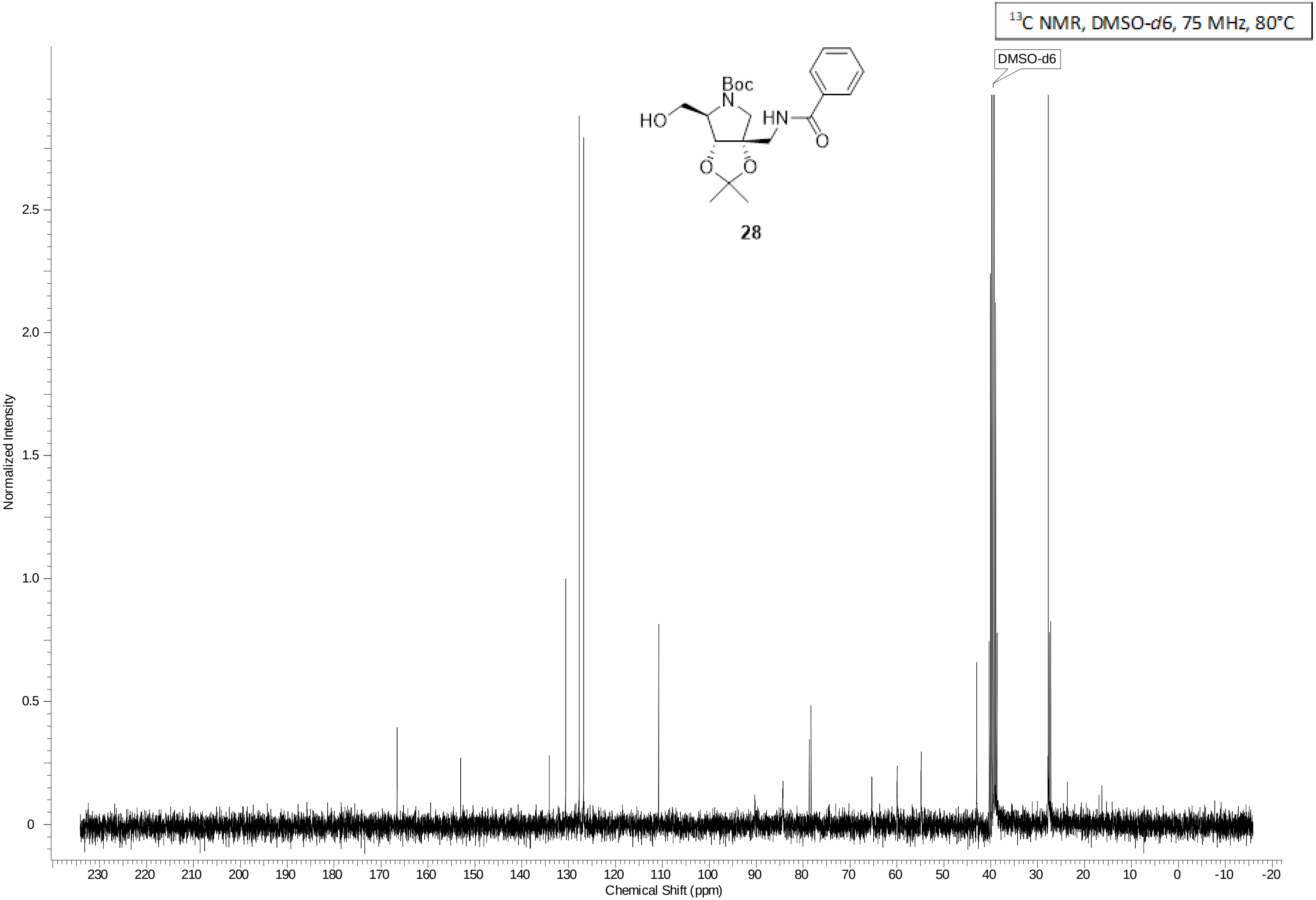

^{13}C NMR, DMSO-*d6*, 75 MHz, 80°C
DMSO-d6
Boc
N
HO
HN
O
O
O
28
Normalized Intensity
2.5
2.0
1.5
1.0
0.5
0
230 220 210 200 190 180 170 160 150 140 130 120 110 100 90 80 70 60 50 40 30 20 10 0 -10 -20
Chemical Shift (ppm)

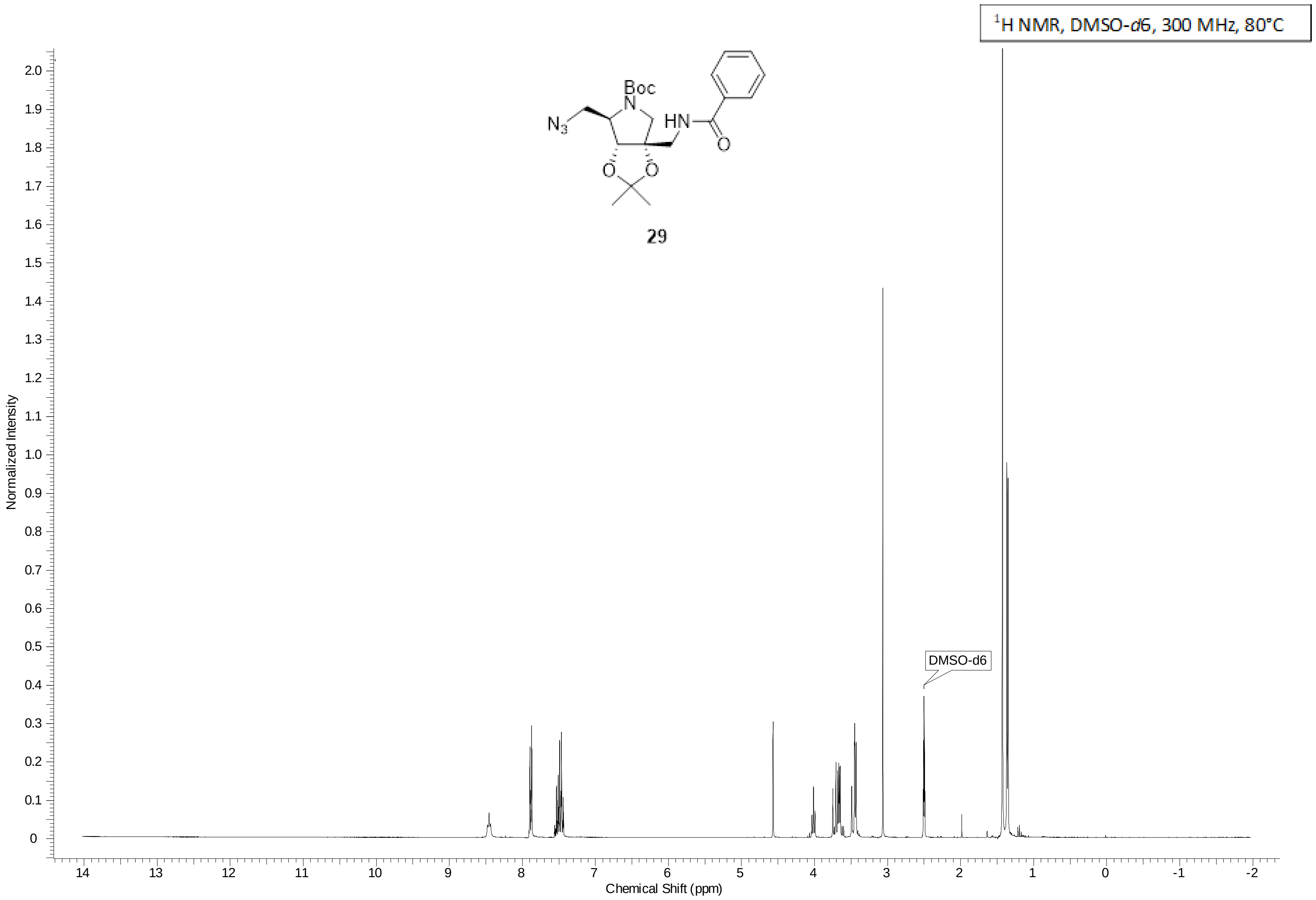
Boc
N
N3
HN
O
O
O
29
1H NMR, DMSO-d6, 300 MHz, 80°C
DMSO-d6
Normalized Intensity
Chemical Shift (ppm)

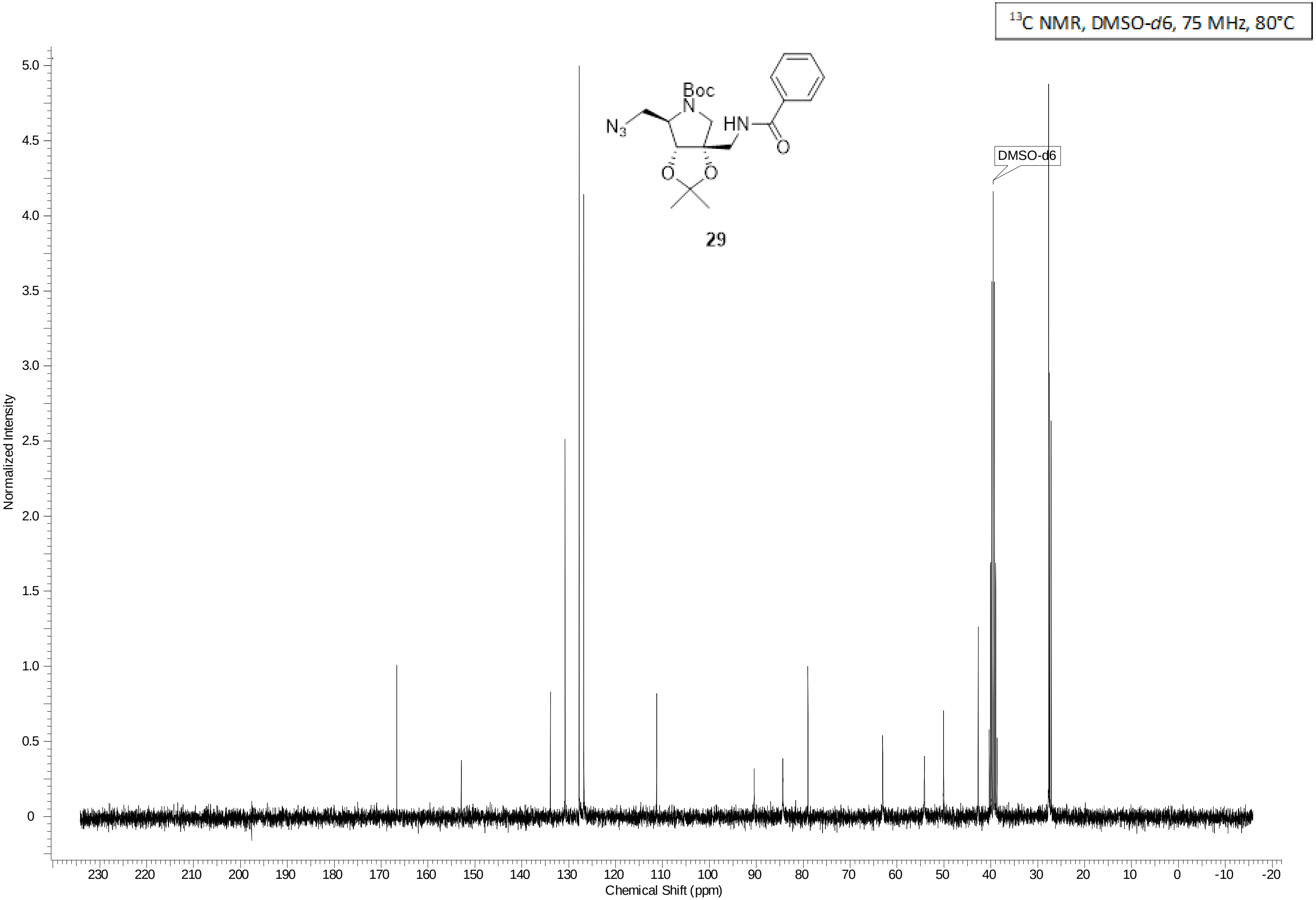

^{13}C NMR, DMSO-*d6*, 75 MHz, 80°C
Boc
N
N_3
HN
O
29
DMSO-d6
Normalized Intensity
Chemical Shift (ppm)

^{1}H NMR, CD_3OD, 300 MHz

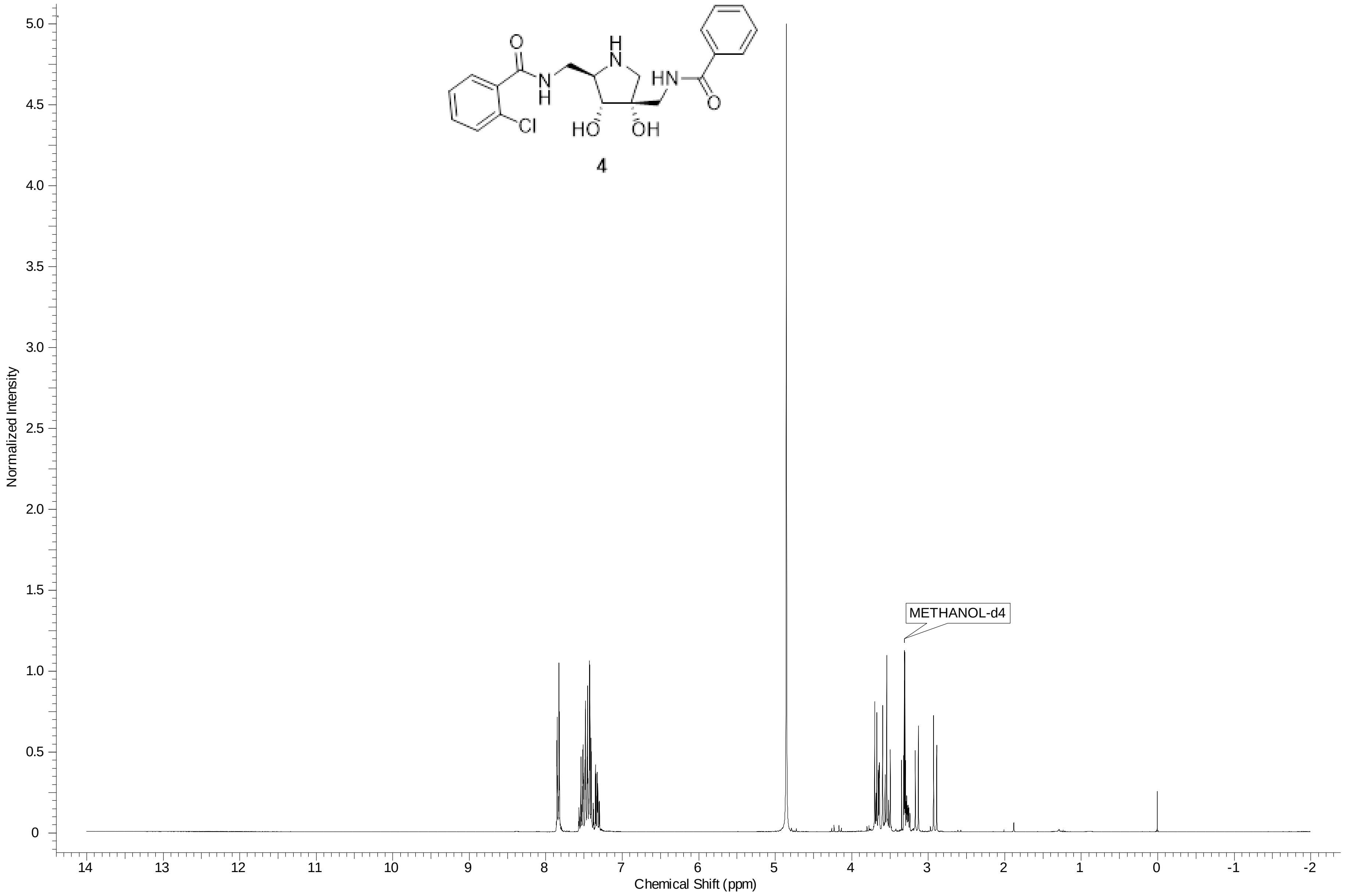

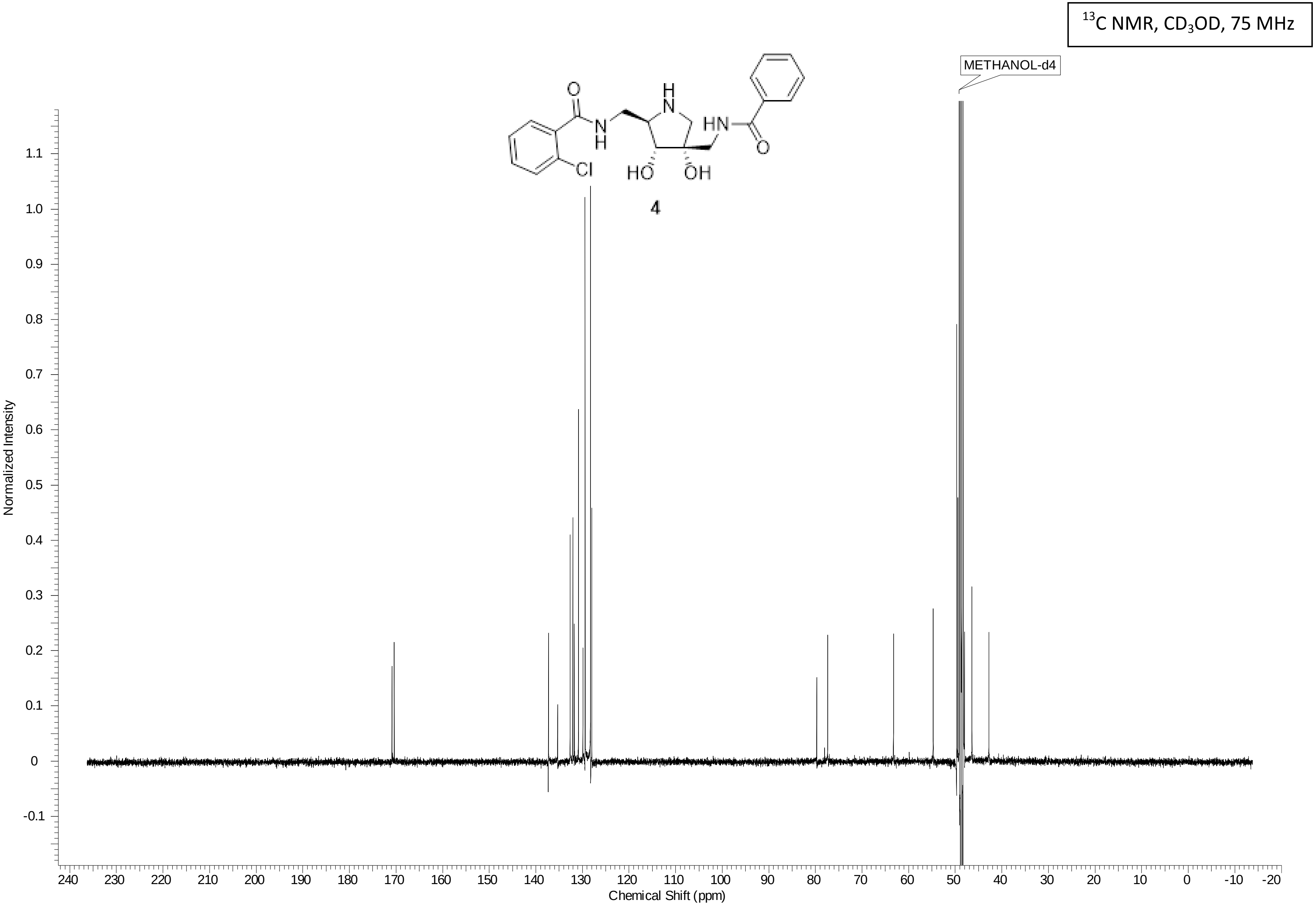

^{13}C NMR, CD_3OD, 75 MHz
METHANOL-d4
O
N
H
H
N
HN
O
Cl
HO
OH
4
Normalized Intensity
Chemical Shift (ppm)

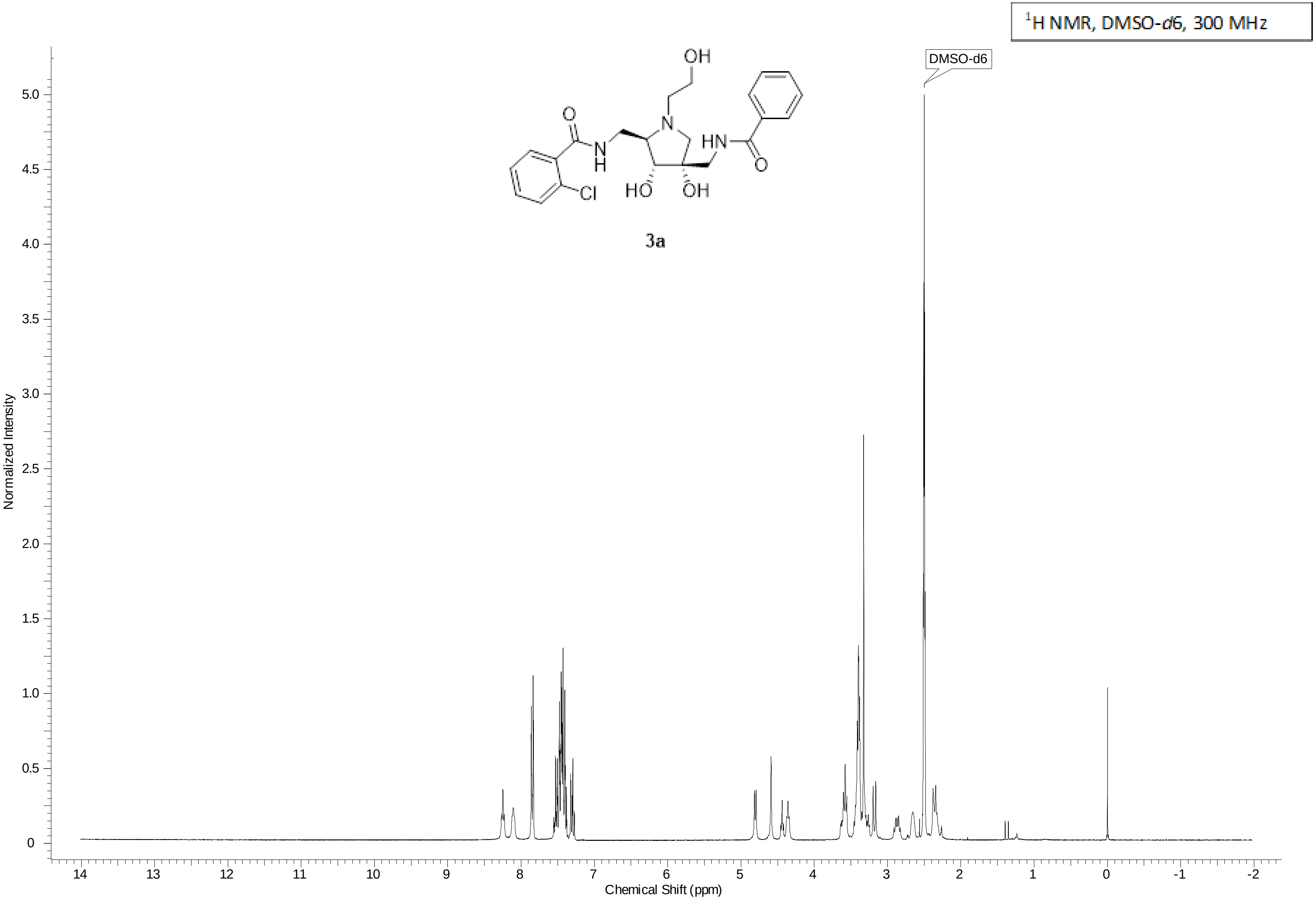

¹H NMR, DMSO-d6, 300 MHz
DMSO-d6
OH
N
HN
O
N
H
Cl
HO
OH
3a
Normalized Intensity
Chemical Shift (ppm)

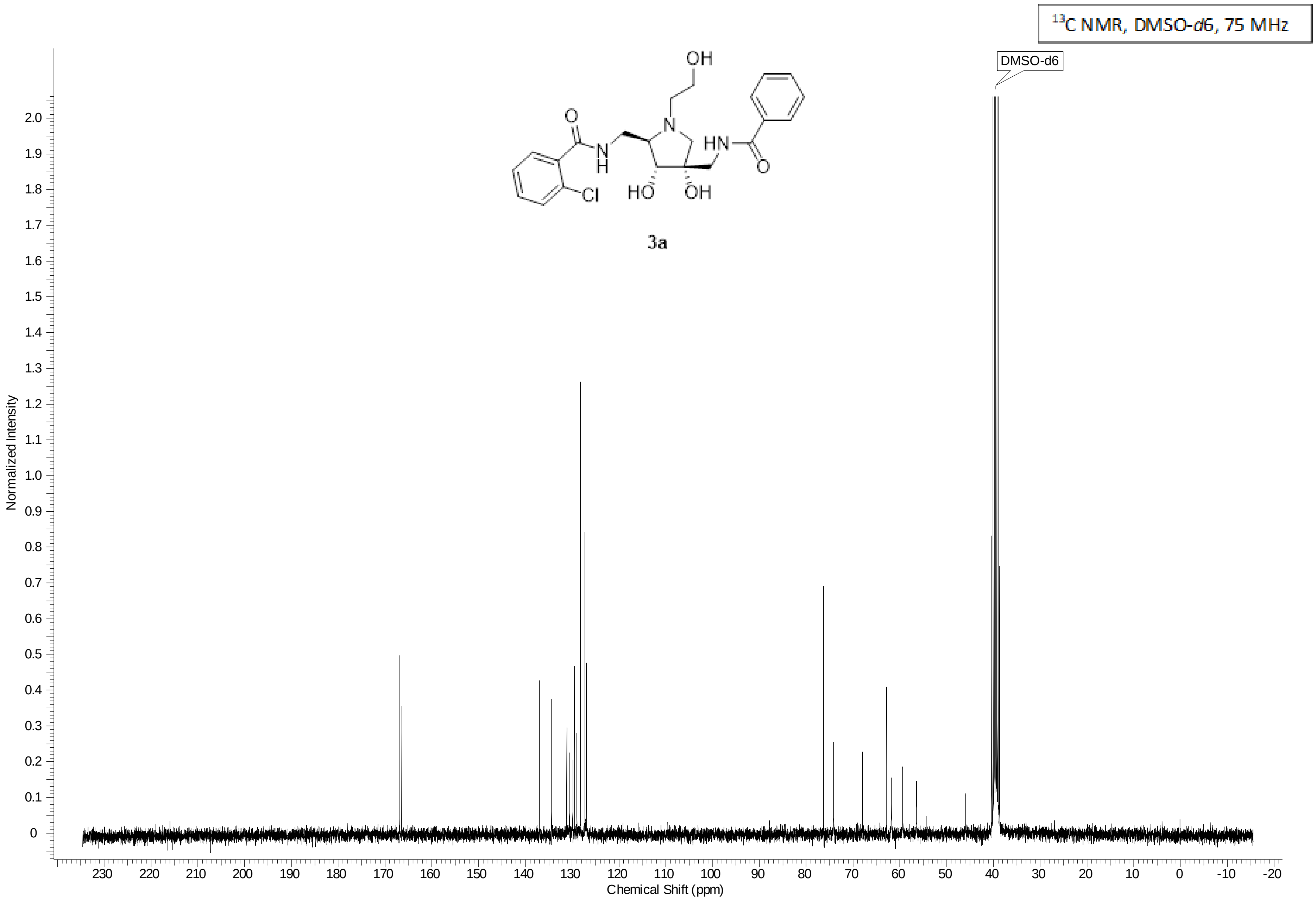
13C NMR, DMSO-d6, 75 MHz
DMSO-d6
3a
Normalized Intensity
Chemical Shift (ppm)

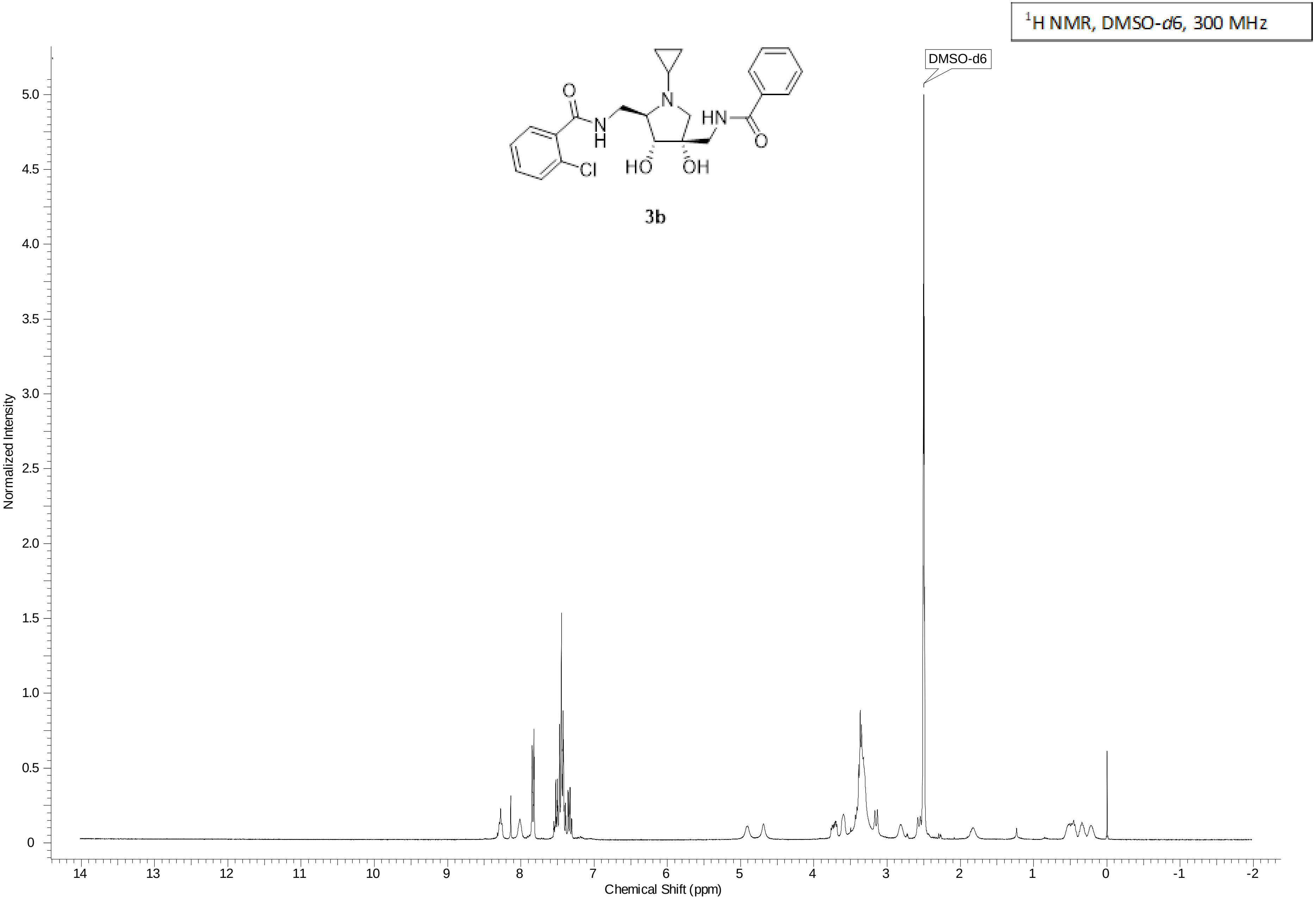

1H NMR, DMSO-d6, 300 MHz
DMSO-d6
O
N
H
Cl
N
HO
OH
HN
O
3b
Normalized Intensity
Chemical Shift (ppm)

^{13}C NMR, DMSO-$d6$, 75 MHz

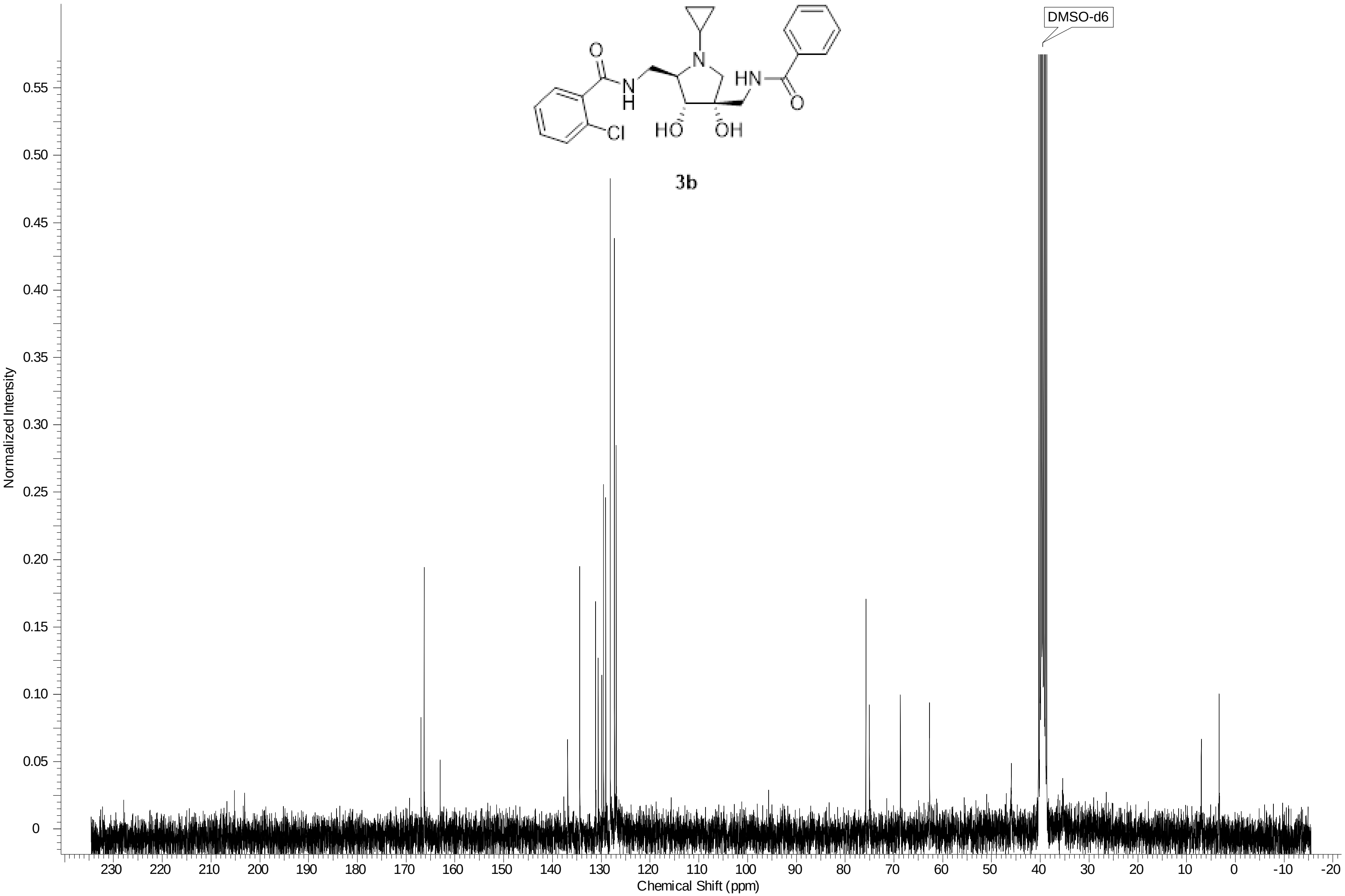

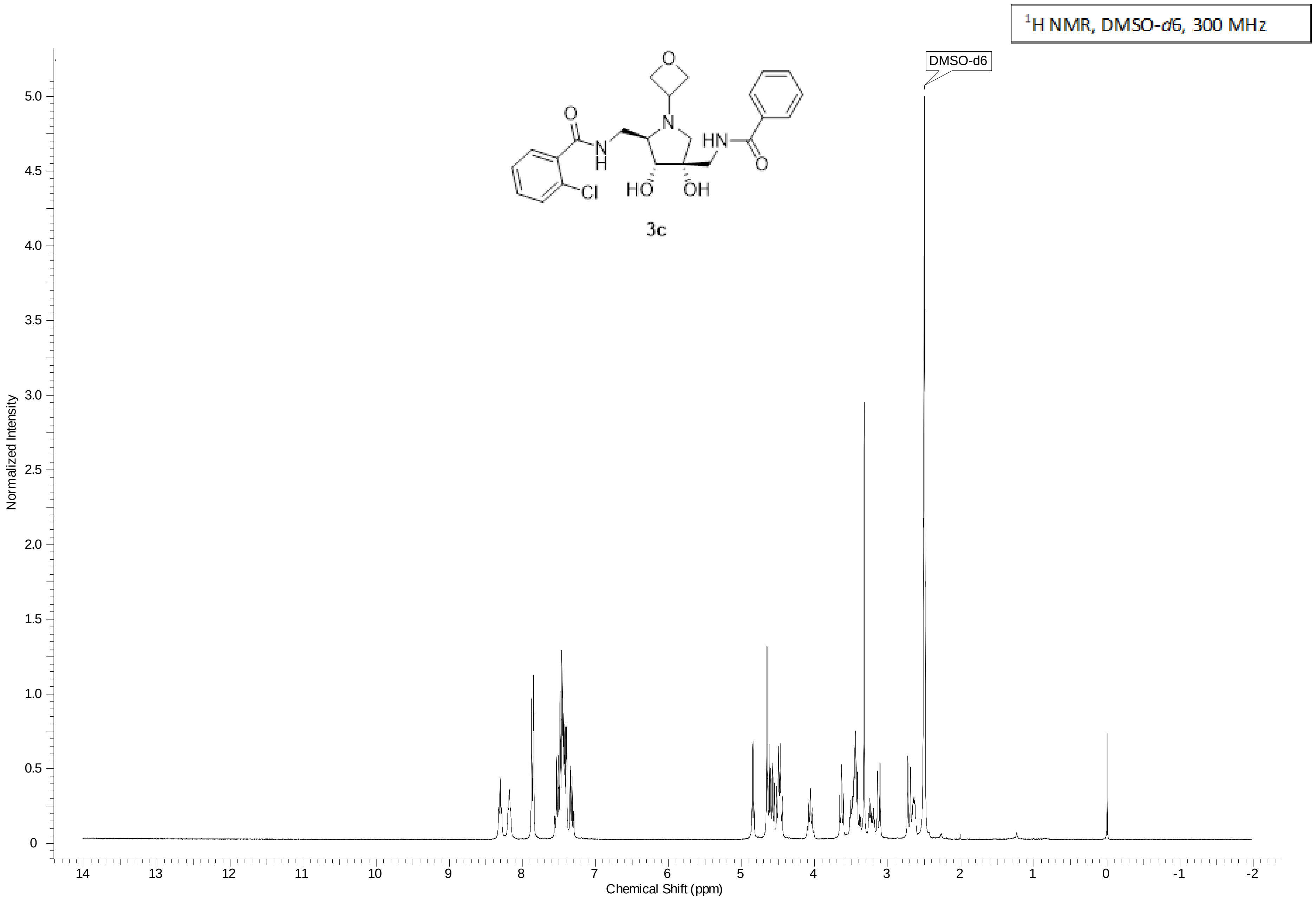

1H NMR, DMSO-d6, 300 MHz
DMSO-d6
3c
O
N
HN
O
H
Cl
HO
OH
Normalized Intensity
Chemical Shift (ppm)

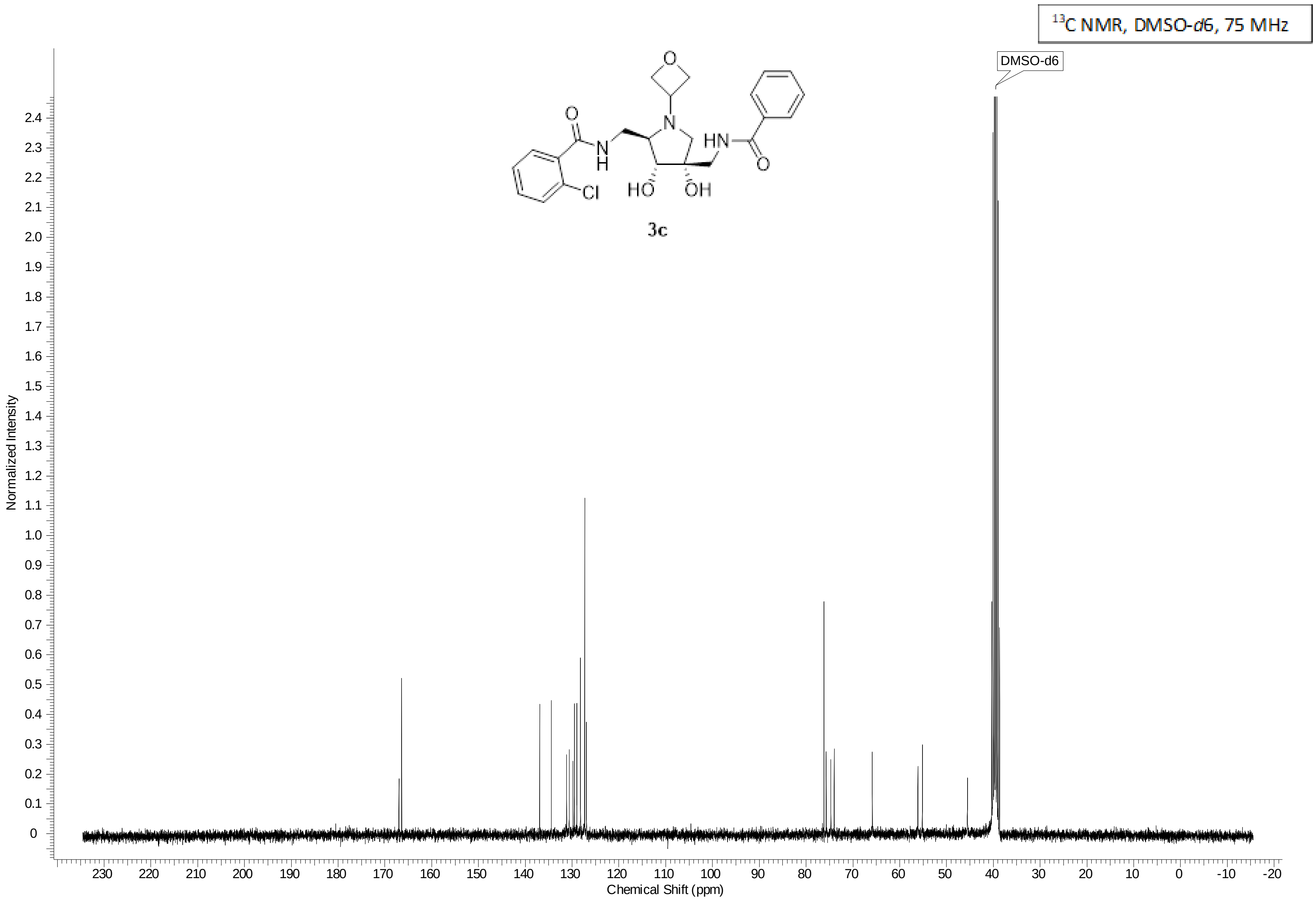
13C NMR, DMSO-d6, 75 MHz
DMSO-d6
3c
Normalized Intensity
Chemical Shift (ppm)

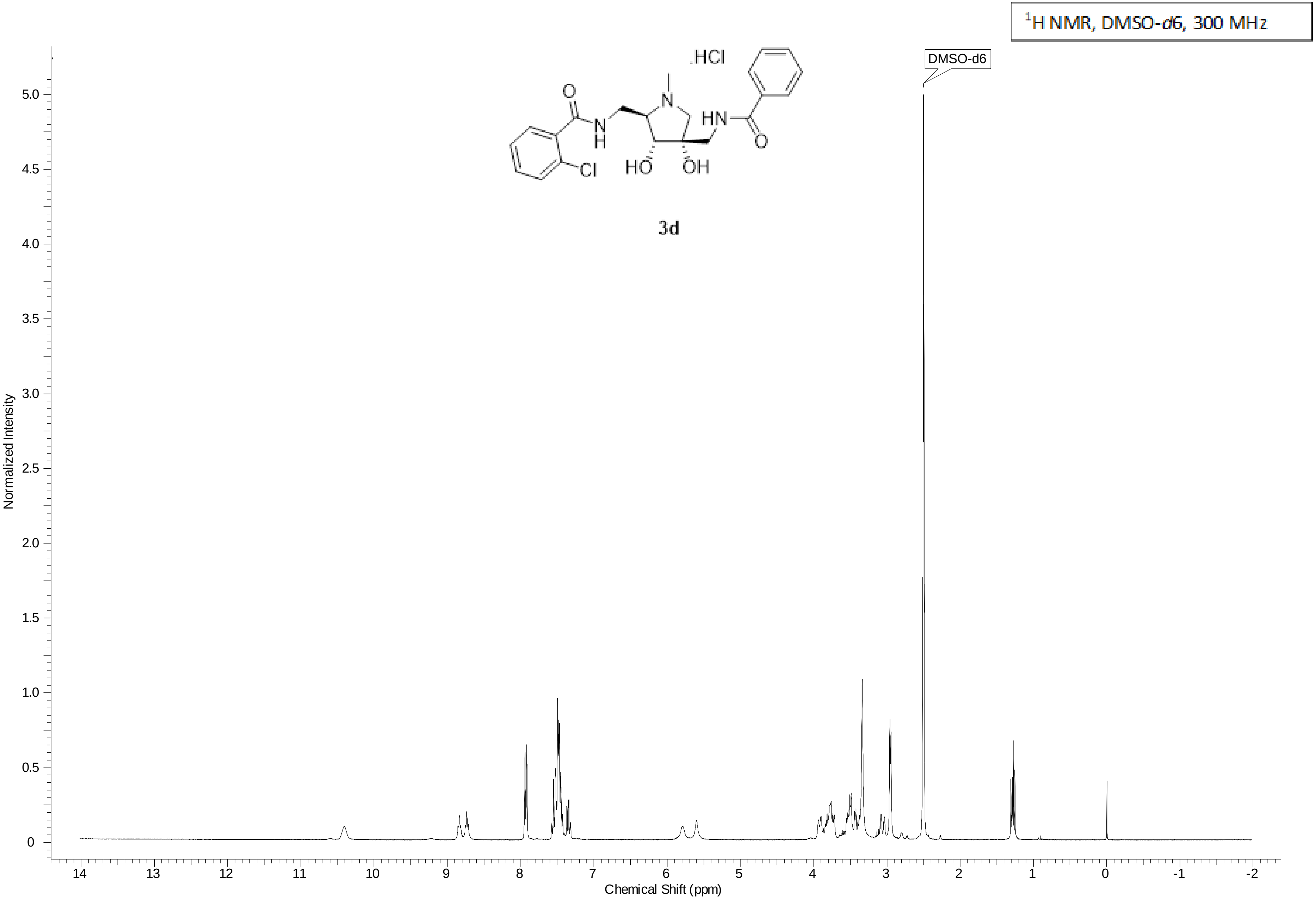

1H NMR, DMSO-d6, 300 MHz
DMSO-d6
.HCl
O
N
H
N
HN
O
Cl
HO
OH
3d
Normalized Intensity
5.0
4.5
4.0
3.5
3.0
2.5
2.0
1.5
1.0
0.5
0
14
13
12
11
10
9
8
7
6
5
4
3
2
1
0
-1
-2
Chemical Shift (ppm)

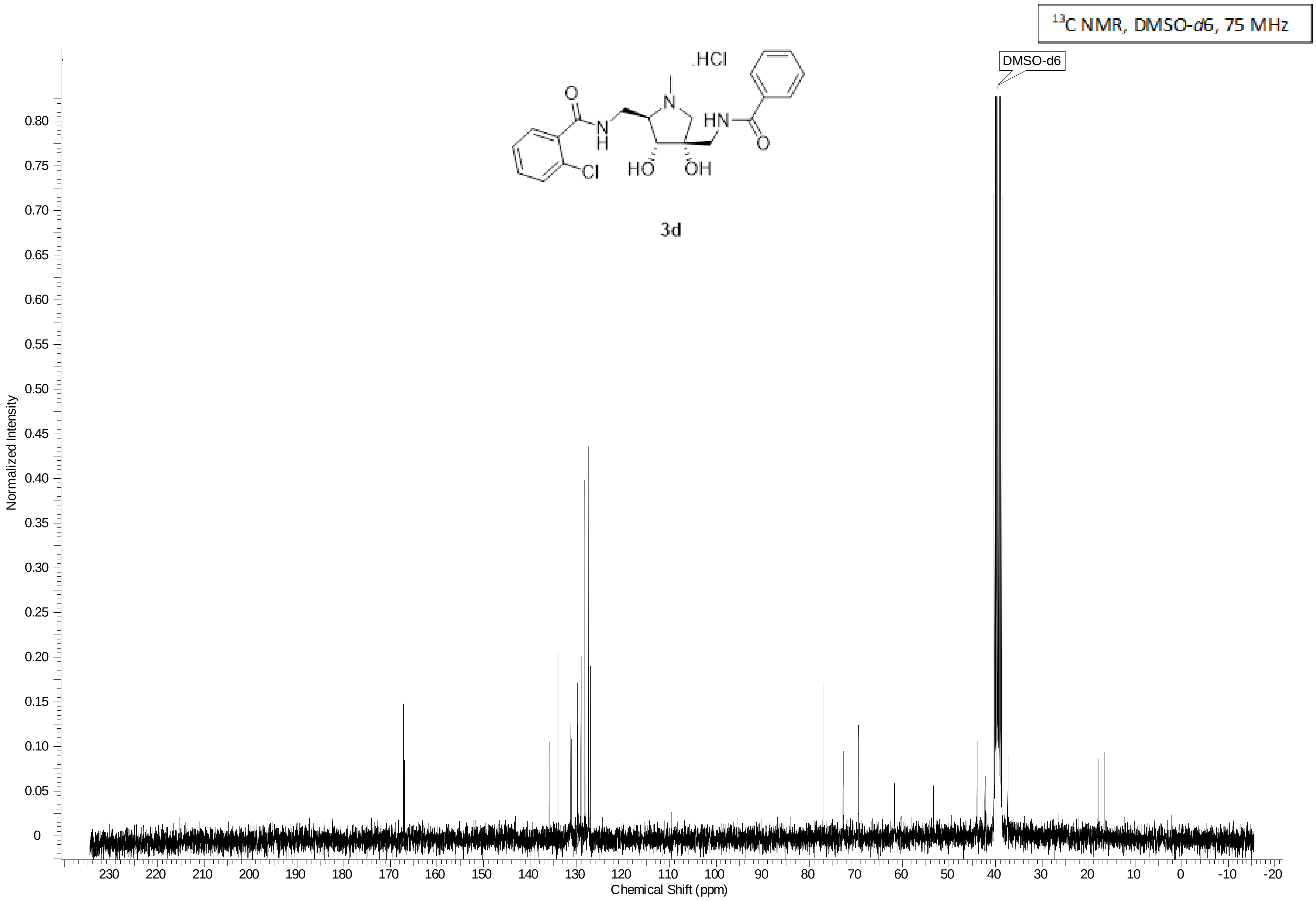

13C NMR, DMSO-d6, 75 MHz
DMSO-d6
.HCl
O
N
H
HN
O
Cl
HO
OH
3d
Normalized Intensity
Chemical Shift (ppm)

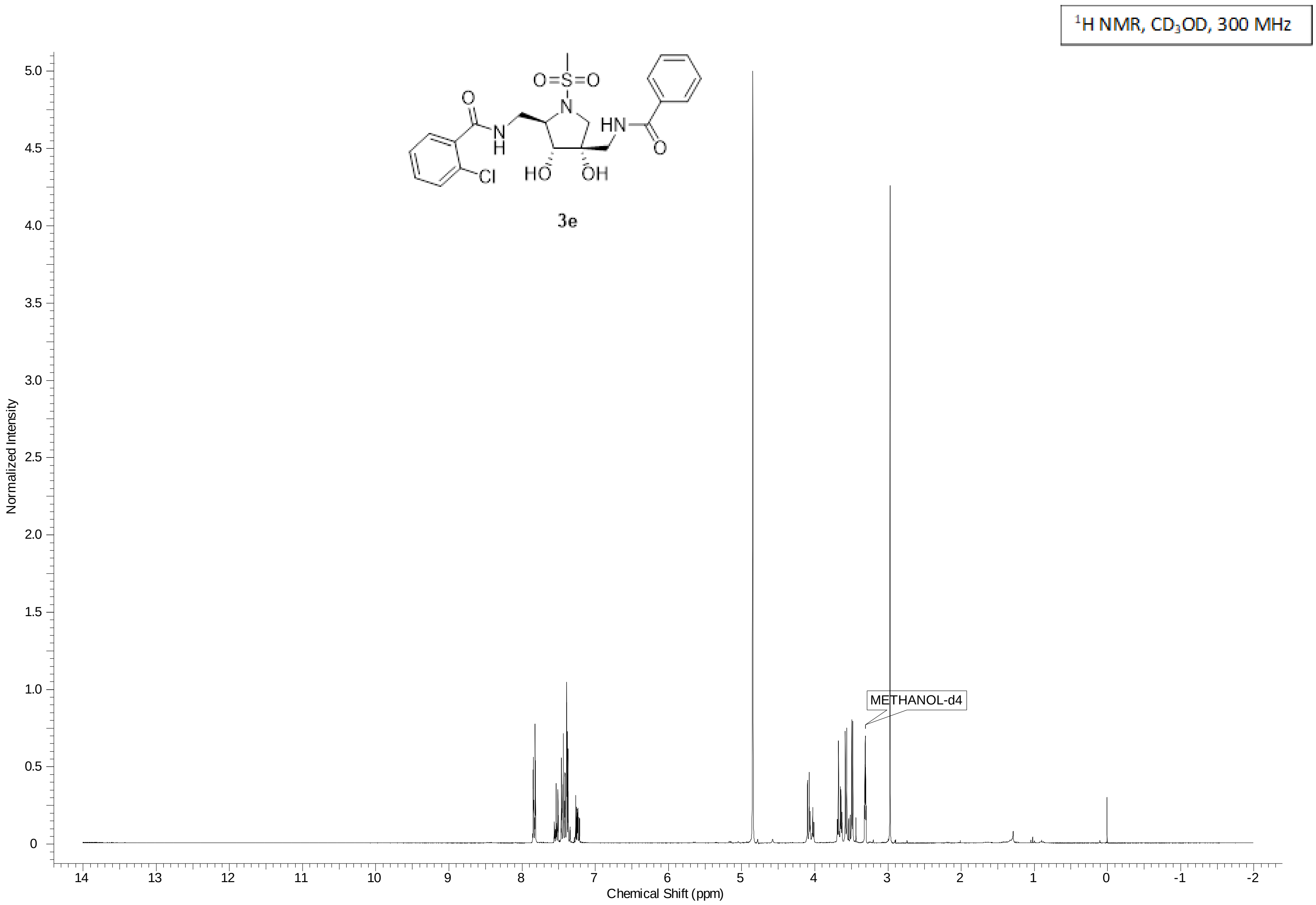

¹H NMR, CD₃OD, 300 MHz
3e
O
O=S=O
N
N
H
HN
O
Cl
HO
OH
METHANOL-d4
Normalized Intensity
Chemical Shift (ppm)

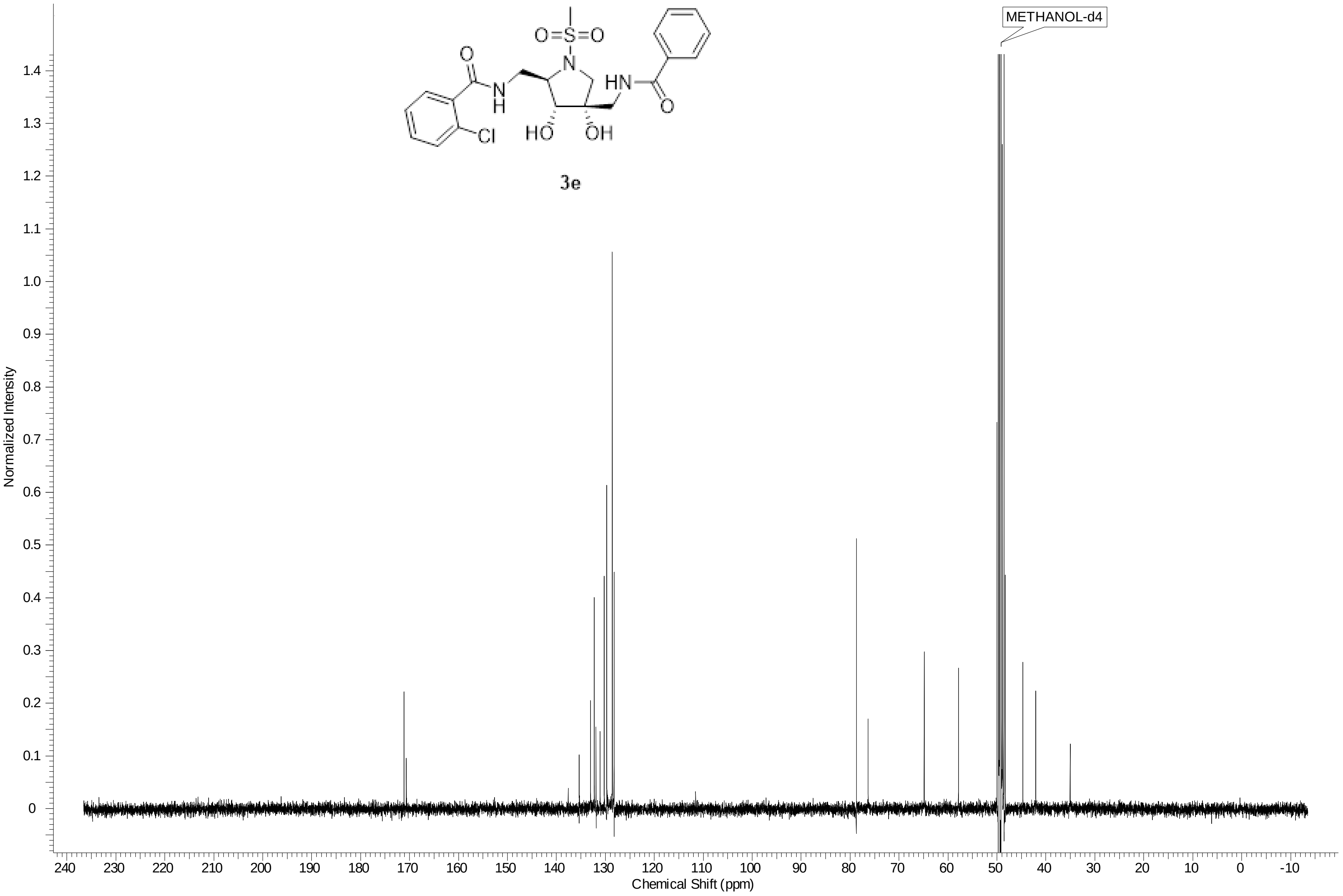
METHANOL-d4
Normalized Intensity
1.4
1.3
1.2
1.1
1.0
0.9
0.8
0.7
0.6
0.5
0.4
0.3
0.2
0.1
0
240 230 220 210 200 190 180 170 160 150 140 130 120 110 100 90 80 70 60 50 40 30 20 10 0 -10
Chemical Shift (ppm)
O=S=O
N
HN
O
N
H
Cl
HO
OH
3e

^{1}H NMR, DMSO-*d6*, 300 MHz, 80°C

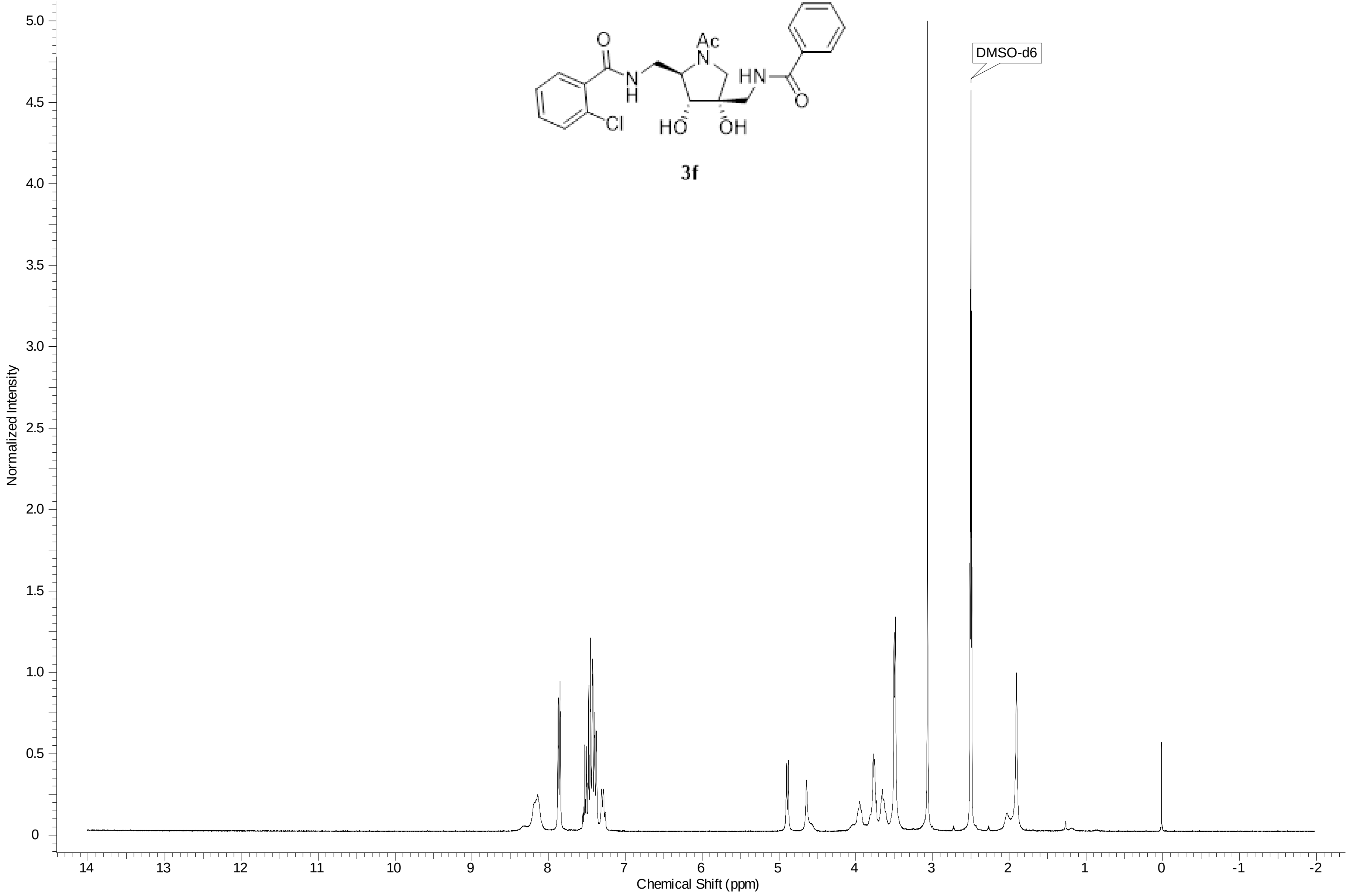

^{13}C NMR, DMSO-*d6*, 75 MHz, 80°C

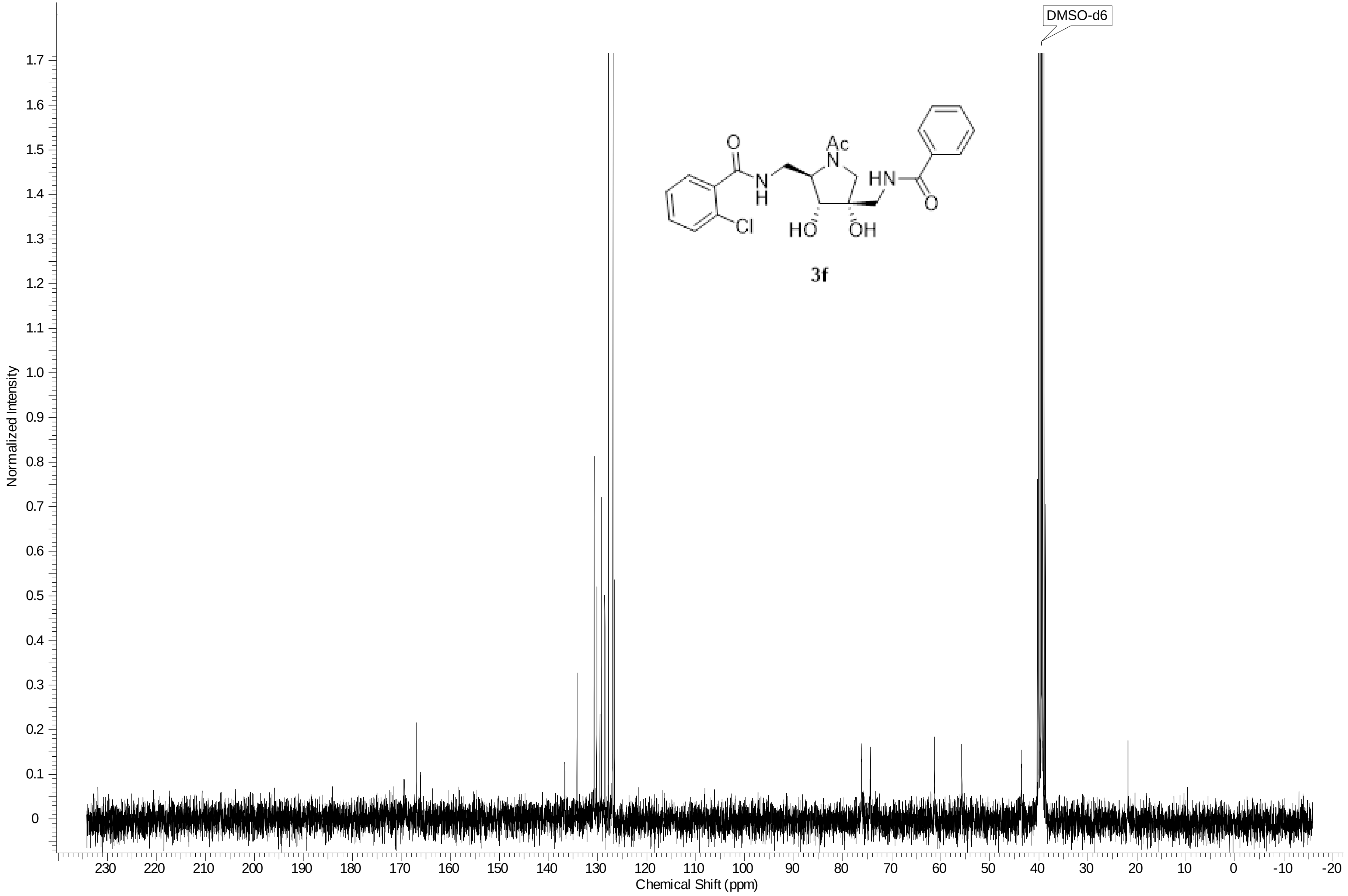

Supplement: File 1 — Experimental details. [file Beilstein_J_Org_Chem-14-2822-s001.pdf]
